# Modelling the Mediterranean Sea ecosystem at high spatial resolution to inform the ecosystem-based management in the region

**DOI:** 10.1038/s41598-022-18017-x

**Published:** 2022-11-16

**Authors:** Chiara Piroddi, Marta Coll, Diego Macias, Jeroen Steenbeek, Elisa Garcia-Gorriz, Alessandro Mannini, Daniel Vilas, Villy Christensen

**Affiliations:** 1 European Commission, Joint Research Centre, Directorate D-Sustainable Resources, Via E. Fermi, 21027 Ispra, VA Italy; 2 Institute of Marine Science (ICM-CSIC), Passeig Marítim de la Barceloneta, nº 39-45, 08003 Barcelona, Spain; 3 Ecopath International Initiative Research Association, Barcelona, Spain; 4 Nature Coast Biological Station, Institute of Food and Agricultural Sciences, University of Florida, Cedar Key, FL 32625 USA; 5 Fisheries and Aquatic Sciences Program, School of Forest Resources and Conservation, University of Florida, Gainesville, FL 32611 USA; 6 Institute for the Oceans and Fisheries, University of British Columbia, 2202 Main Mall, Vancouver, BC V6T 1Z4 Canada

**Keywords:** Ecological modelling, Ecological modelling, Environmental impact, Zoology, Climate sciences, Ecology, Ocean sciences

## Abstract

Cumulative pressures are rapidly expanding in the Mediterranean Sea with consequences for marine biodiversity and marine resources, and the services they provide. Policy makers urge for a marine ecosystem assessment of the region in space and time. This study evaluates how the whole Mediterranean food web may have responded to historical changes in the climate, environment and fisheries, through the use of an ecosystem modelling over a long time span (decades) at high spatial resolution (8 × 8 km), to inform regional and sub-regional management. Results indicate coastal and shelf areas to be the sites with highest marine biodiversity and marine resources biomass, which decrease towards the south-eastern regions. High levels of total catches and discards are predicted to be concentrated in the Western sub-basin and the Adriatic Sea. Mean spatial–temporal changes of total and commercial biomass show increases in offshore waters of the region, while biodiversity indicators show marginal changes. Total catches and discards increase greatly in offshore waters of the Western and Eastern sub-basins. Spatial patterns and temporal mean changes of marine biodiversity, community biomasses and trophic indices, assessed in this study, aim at identifying areas and food web components that show signs of deterioration with the overall goal of assisting policy makers in designing and implementing spatial management actions for the region.

## Introduction

Marine ecosystems provide essential services for people, such as food, energy and mineral resources, climate regulation and recreation, and they are crucial for local economies^1,2^. Yet, these ecosystems continue to be threatened by multiple pressures and impacts from human activities, such as fishing, seabed disturbance, pollution and global warming^3,4^. The ecosystem-based management (EBM) framework has been pursued as one of the most appropriate strategy in preserving marine ecosystems since it directly aims at managing the multiple human activities impacting these systems, and accounts for the direct and indirect pressure-impact effects when making management decisions^5,6^. In Europe, the adoption of EBM is embedded in four main policy instruments for marine governance:

- the Marine Strategy Framework Directive (MSFD; 2008/56/EC), which aims to achieve or maintain ‘Good Environmental Status’ (GES) of the European marine waters;
- the EU Biodiversity Strategy to 2030 (COM (2020) 380), which aims at halting the loss of biodiversity and ecosystem services by protecting at least 30% of the sea and establishing a network of marine protected areas (MPAs);
- the Common Fishery Policy (CFP, 1380/2013), which aims, among other targets, at moving from the traditional single stock management towards an Ecosystem-Based Fisheries Management (EBFM);
- and the Maritime Spatial Planning Directive (MSP, EC 2014/89/EU), which aims at promoting the sustainable growth of maritime economies, the sustainable development of marine areas and sustainable use of marine resources.

In addition, in recent years, the European Commission adopted the “Farm to Fork Strategy”^7^ and the Action Plan: "Towards a Zero Pollution for Air, Water and Soil"^8^, which aim at making food systems fair, healthy, and environmentally friendly (e.g., mitigating climate change and preserving environment/biodiversity) and at reducing pollution (e.g., air pollution, noise pollution, pesticides, plastics, waste) to levels no longer considered harmful to health and natural ecosystems.

All these policies constitute important milestones of the roadmap initiated by the European Commission to achieve the European Green Deal^9^, which aspires to “protect the health and well-being of citizens from environment-related risks and impacts” and establish a non-toxic and plastic-free environment, deliver healthy and sustainable diets, and protect biodiversity. Although the first legislation to move towards a holistic approach for managing the marine environment was established in 2008, the uptake of EBM in a policy context has been very slow^10^.

There are numerous reasons for this, including the complexity of assessing and managing the marine environment, the conflicting influence of short-term sub-national and national interests in the decision-making process, the lack of societal and political will to fund and enforce the needed measures, the low compliance to legislation, and the lack of involvement of other societal sectors (e.g., economic, private, shipping and energy sectors)^11^.

To counteract some of the issues related to the complexity of assessing the marine environment (e.g., considering its structure and functioning), policy makers increasingly endorse the use of marine ecosystem modelling tools in the decision making process^12^. Such modelling can be used, in fact, to explore the direct and indirect ecological responses occurring when alternative management scenarios are proposed^13,14^. To fully utilize these tools in an evidence-based policy context, marine ecosystem models are required to be spatially and temporally explicit to facilitate the communication between scientists and policy makers in identifying species/group of species and areas of major concern and planning targeted actions such as MPA site selections^15^, catch level reductions^16^ and nutrients control measures^14^. Spatial marine ecosystem modelling is, however, a challenging component of higher trophic level (HTL) ecosystem approaches (e.g., models that consider food web dynamics beyond phytoplankton and zooplankton and usually include main food web components)^14,17^ and, for this reason, its use in the policy making process has been limited. Main obstacles reside, for example, with the lack of spatial data for many species and ecosystems^18^, and with the difficulties of accounting for environmental effects, trophic interactions, and human activities (e.g., fishing) when predicting the distribution of marine species in space and time^19^.

In recent years, new developments and acquired knowledge have enabled advances in this sector. In particular, food web modelling approaches, like Ecopath with Ecosim (EwE)^20^, can now spatially drive in time^21,22^ marine species influenced by the cumulative effects of multiple physical, oceanographic, and environmental conditions, in conjunction with accounting for food-web interactions and anthropogenic stressors, e.g., fisheries, aquaculture, renewable energy, land modification and infrastructures^23–26^.

In this study, we applied the new developments of the EwE modelling approach to first quantify and analyse in space and time (1995–2016) past ecosystem dynamics using a set of ecological indicators and then evaluate spatial–temporal historical (1995–2016) responses of these indices to changes in environmental factors and fisheries in the Mediterranean Sea. This basin is a hotspot of marine diversity^27^ and yet it is one of the Large Marine Ecosystems in the world that is most threatened by multiple anthropogenic pressures^28,29^, with severe signs of degradations at species-, community- and ecosystem levels^27,30^. The spatial–temporal analyses planned in this study aim at identifying areas and ecosystem components that show signs of deterioration, with the overarching goal to identify past trajectories and support future marine policy actions in the region.

## Results

### Spatial-dynamic indicators: mean distribution

The spatial biomass distribution of all the functional groups in the model (from planktonic-habitat groups to invertebrates, pelagic and demersal fish, sea turtles, marine mammals and marine birds) was hindcasted, for the 1995–2016 period, considering their ecological niche and their trophic interactions with prey and predators. Estimated spatial patterns were heterogeneous among the different functional groups of the Mediterranean Sea (Supplementary Figs. S6–S10 in S6 of the Supporting Information): for example, top predators like cetaceans, tunas or swordfish, being highly mobile, were predicted to distribute widely across the basin as well as multi-species groups such as mesopelagic fish, mesopelagic cephalopods, bathydemersal fish and plankton (e.g., zooplankton and jellyfish). For the majority of the other functional groups, which included demersal and pelagic fish, invertebrates and habitat-formers (e.g., seagrass), the model predicted a distribution mainly along the Mediterranean coastline and continental shelf.

When spatially validating the predicted European hake biomass, mixed correlations were observed (Supplementary Fig. S11 in S6). In particular, a positive correlation was observed along the Italian (Western and Ionian) coasts, in few patches of the Western Mediterranean islands, the North Adriatic and North Aegean Seas. Negative correlation was observed in the Adriatic Sea and a few areas of the Spanish coast. The Eastern side only showed few data points with positive correlations. The correlation test performed for bottom trawlers and purse seiners effort showed a general positive correlation along the coasts and shelves of the Western and Ionian Seas and partly of the Adriatic and the Aegean/Levantine Seas; negative correlations were observed in the Adriatic Sea, along the coasts of the Greek Ionian Sea, in the North Aegean and Levantine Seas, and few coastal/shelf areas of the Western Mediterranean Sea (e.g., Sicilian Channel).

**Figure 1:**
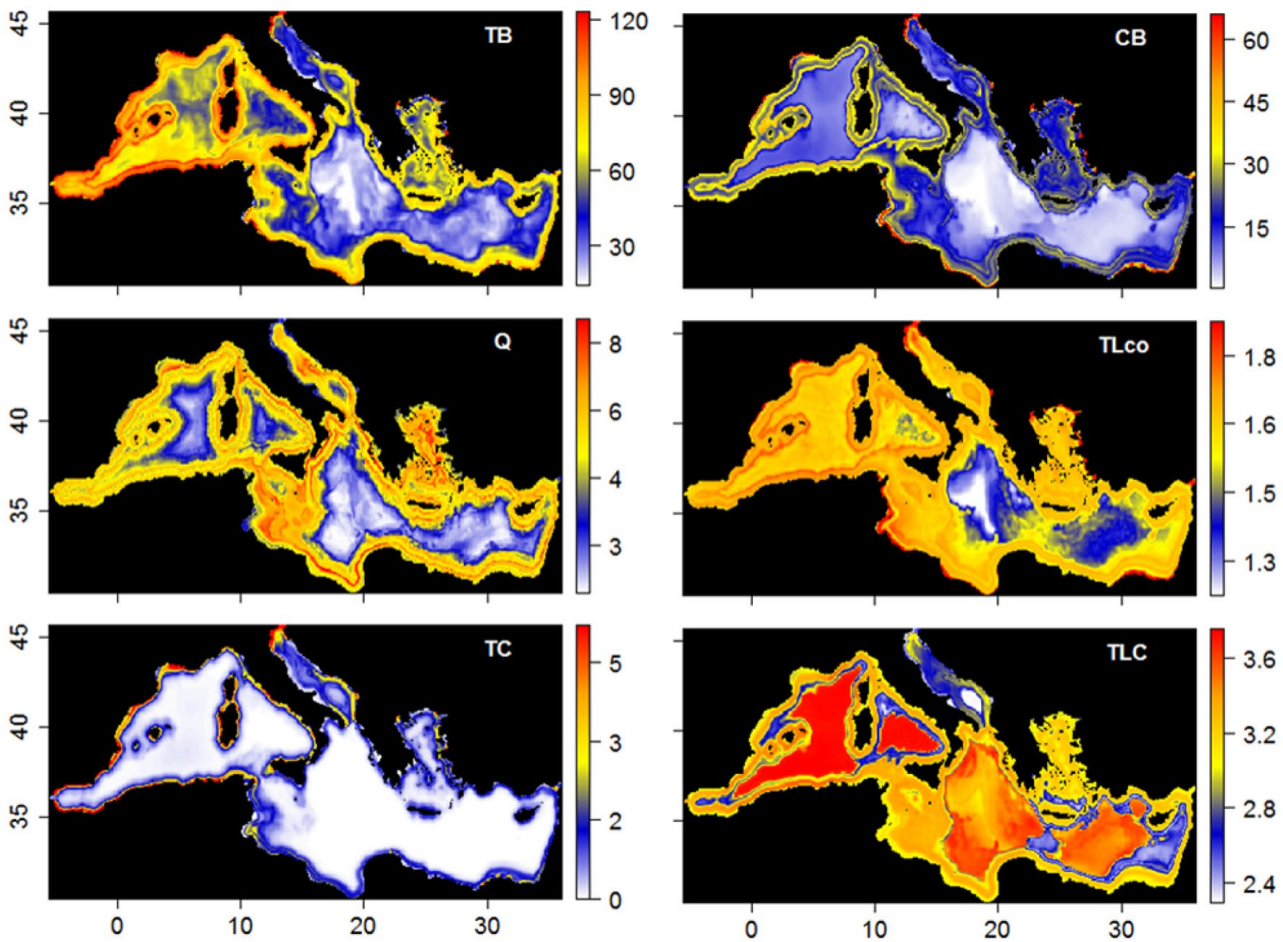
Mean (1995–2016) of selected modelled indicators. *TB* Total biomass (t/km^2^), *CB* commercial biomass (t/km^2^), *Q* Kempton index, *TL*_*co*_ Trophic level (TL) of species/Fg in the community, *TC* Total catch (t/km^2^/year), *TLC* Trophic level (TL) of the catch.

Looking at community indicators, total biomass (TB, Fig. 1) showed higher values in the Western Mediterranean Sea and along the coast and continental shelf and close to main estuaries of all the basin. A similar picture was observed for commercial biomass (CB, Fig. 1) but with less concentration in the Eastern side and in the Adriatic Sea. The two community ratios, Demersal/Pelagic fish biomass (D/P) and Invertebrates/Fish biomass (I/F) (Supplementary Fig. S12 in S6), showed more demersal fish and invertebrates’ biomasses than pelagic fish and total fish, respectively, and this biomass was generally higher along the coasts/shelves and, in the case of invertebrates, also in deeper and more offshore areas. The ecosystem-scale biodiversity indices (Kempton Q: Fig. 1Q; Shannon: Supplementary Fig. S12H) showed high biodiversity around all the Mediterranean coastline/shelf, with greater concentration in the Adriatic Sea, the Sicilian channel and the northern parts of the Aegean and the Western seas. Lower values were observed in deep and offshore areas of the basin. Conversely, differences were observed in trophic indices when looking at trophic levels of the communities, with or without the planktonic groups. In particular, TL_co_ (Fig. 1, which included phytoplankton groups) was higher close to main estuaries and around the continental shelf, and mainly in the Western part, while TL_co2_ and TL_co3.25_ (Supplementary Fig. S12 in S6) was greater in the Western Mediterranean Sea, both offshore and close to the coast, and in few patches of the Adriatic, Ionian Sea and Aegean/Levantine Seas. Lower values were observed in some deep areas of the Ionian Sea, the coastline in the southern Mediterranean Sea and, for TL_3.25_, also in the Adriatic Sea.

As for catch related indicators, the model predicted total catches (Fig. 1-TC) to be along the coasts and the shelves of the basin with increases mainly in the Western Mediterranean and the Adriatic Sea. Discards followed a similar pattern, yet showing greater concentration in the Adriatic Sea; trophic level of the catches were higher in deeper areas of the basin and along the continental shelf. The Adriatic Sea was the area with the lowest trophic level of the catch (TLC).

### Spatial-dynamic indicators: mean temporal change

**Figure 2:**
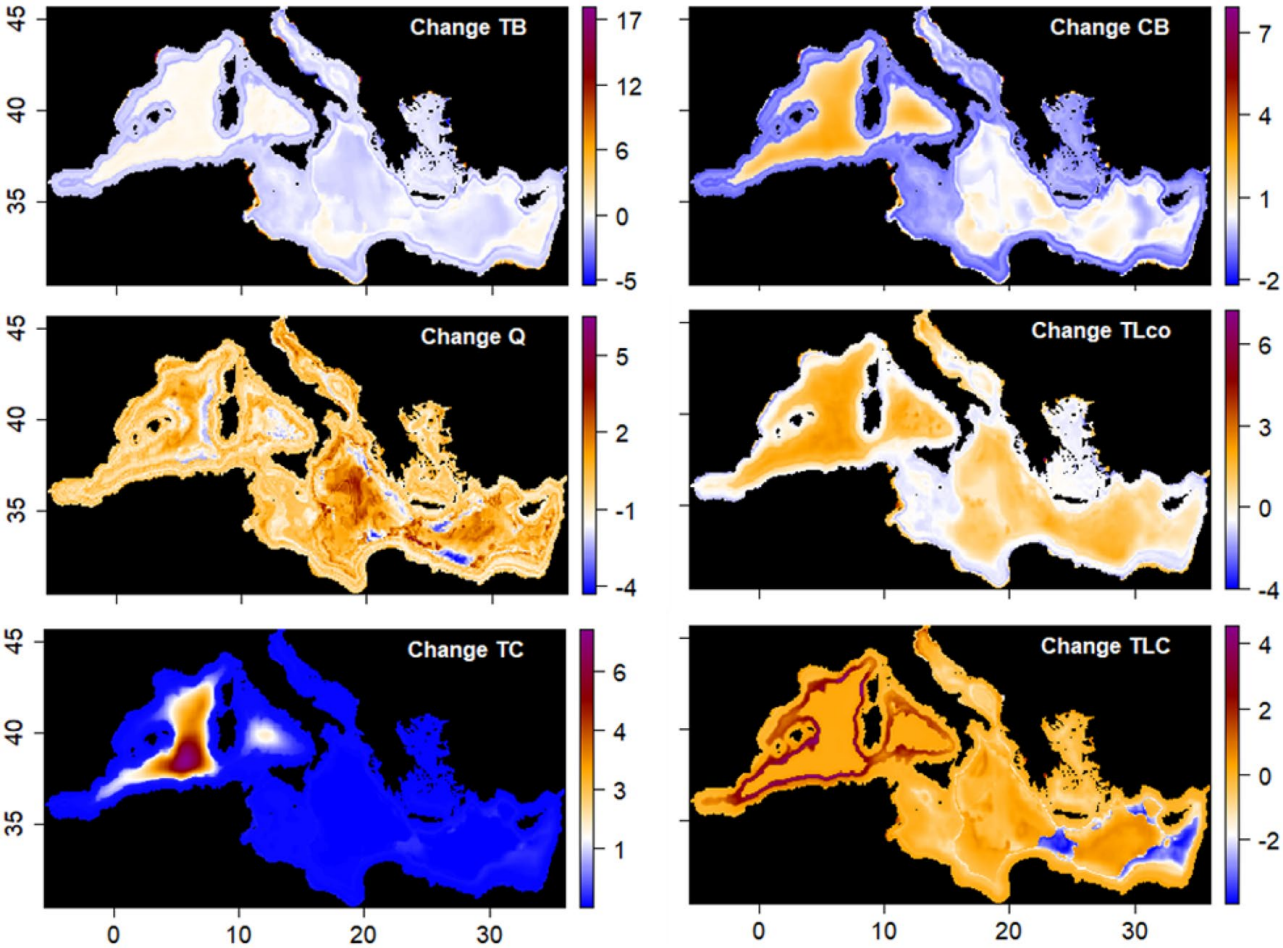
Mean (1995–2016) change (%) for selected modelled indicators. *TB* Total biomass, *CB* commercial biomass, *Q* Kempton index, *TLco* trophic level (TL) of species/Fg in the community, *TC* total catch, *TLC* trophic level (TL) of the catch. To facilitate the comparison, indicators were standardized (difference from the mean divided by the corresponding standard deviation).

The mean change computed for TB highlighted a marginal decrease (between − 1 and − 4%, Table 1) in biomass along the main coasts/shelves and slopes of the basin (Fig. 2 and Table 1). Slight increases (between 1 and 6%, Table 1) were, instead, observed around main estuaries (e.g., Po, Nile, Rhone, Majardah, Evros, see Fig. 4h) and in deeper areas of the region, mainly in the Western Mediterranean Sea (Table 1). CB followed a similar pattern but, contrarily to TB, it evidenced a more pronounced decrease in biomass along the coasts/shelf and slope areas of the Western Mediterranean Sea (~ − 10%) and of the shelf in the Ionian Sea (~ − 9%). Increases in biomass were shown in some coastal areas of the south Mediterranean Sea (Ionian [12%] and Eastern [8%] sub-regions) and of the Adriatic Sea (16%), and mostly in offshore deeper areas (between 10 and 69%, Table 1 and Fig. 2). Changes in D/P showed an overall increase in demersal fish in all the basin, particularly pronounced in coastal and bathyal areas of the basin, while shelf and slope areas of the Western Mediterranean Sea indicated a decline (Table 1 and Supplementary Fig. S13 in S6). A different picture was shown for the I/F where major reductions in invertebrate biomass were observed in the Western Mediterranean Sea (between − 14 and − 53%) and marginally (between − 2 and − 6%) in deeper areas of the Adriatic Sea and in shelf and bathyal areas of the Ionian Sea. Increases (Supplementary Fig. S13 in S6 and Table 1) were mainly observed in coastal and shelf areas of the Adriatic Sea (61% and 40% respectively) and in coastal and deeper areas of the Ionian (coast: 24%, slope: 13%) and Eastern- Levantine (coast: 18%; slope: 10%; bathyal: 26%) seas.

**Table 1:**
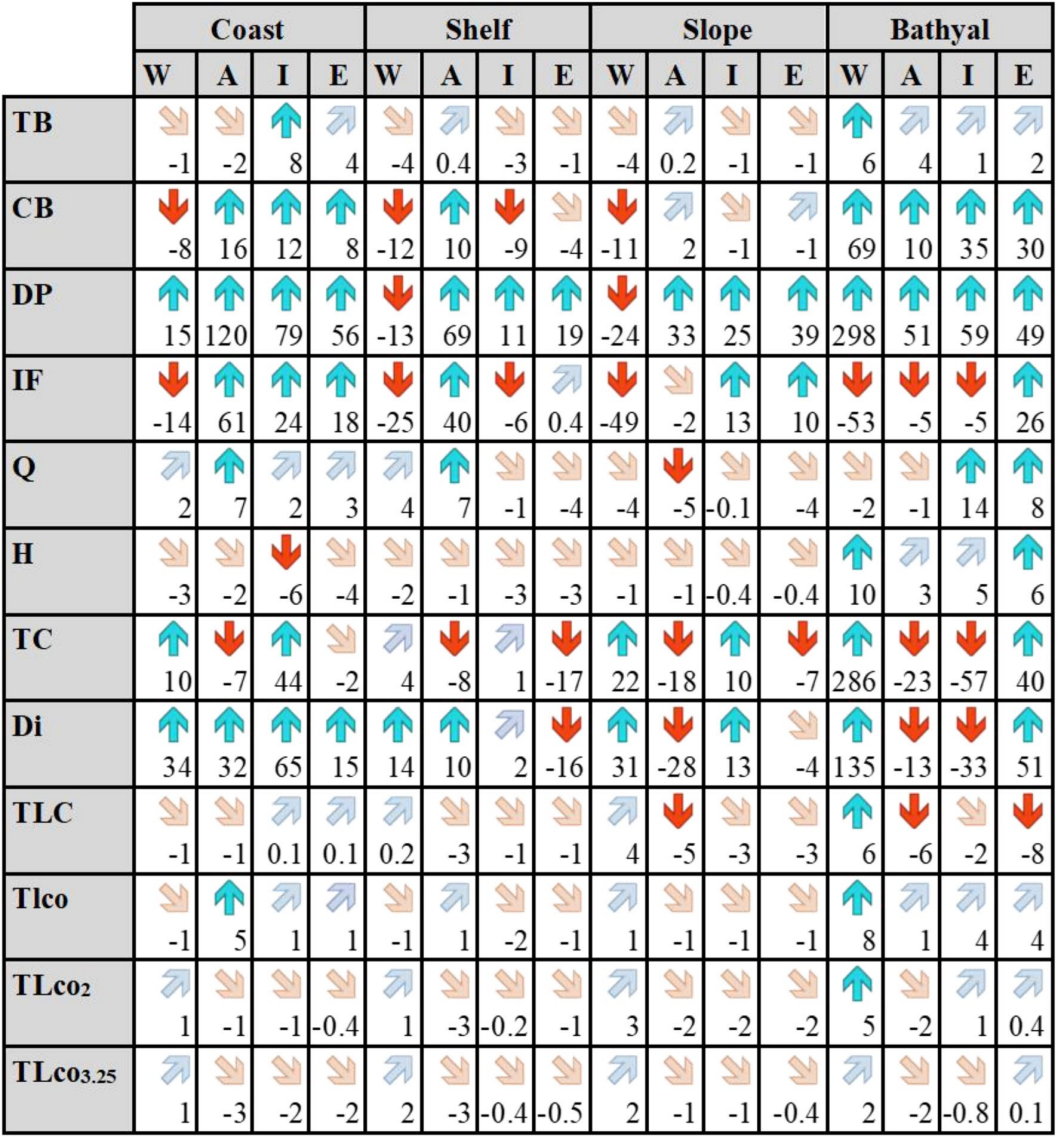
Relative mean change (%) per assessed indicator (*TB* Total biomass, *CB* commercial biomass, *DP* Demersal/Pelagic fish, *IF* Invertebrates/Fish, *Q* Kempton index, *H* Shannon index, *TC* total catch, *Di* discards, *TLC* trophic level (TL) of the catch, *TLco* trophic level (TL) of species/Fg in the community, *TLco2* trophic level (TL) in the community with TL ≥ 2; Trophic level (TL) in the community with TL ≥ 3.25) divided per MSFD sub-region (*W* Western, *A* Adriatic, *I* Ionian, *E* Eastern) and per bathymetric feature (Coast: 0–50 m; Shelf: 51–200 m; Slope: 201–800 m; Bathyal: > 800 m). Red arrow represents mean change < − 5%; pink arrow between − 5% and 0; blue arrow > 5%; light blue between 0 and 5%.

Regarding the two biodiversity indices, Kempton Q (Fig. 2) showed variability in the region with slope areas having marginal reductions (between − 1 and − 5%) and coastal waters slight increases (between 2 and 7%), while Shannon H evidenced a small reduction (between − 0.4 and − 6%) in coastal/shelves/slopes areas and increases in the deeper waters of the region (between 3 and 10%) (Supplementary Fig. S13 in S6 and Table 1).

As for the trophic level of the community (TL_co_, Fig. 2, Supplementary Fig. S13 in S6 and Table 1), results showed small decreases and increases (between − 1 and 5%) along the coasts/shelves/slopes of the basin, while deeper offshore waters, particularly in the Western Mediterranean, showed the highest increases (8%). The other two TL of the communities (TL_co2_ and TL_co3.25_, Supplementary Fig. S13 in S6, Table 1) displayed again marginal declines and increases (between − 3% and 5%) in the whole region.

Total catch (TC in Fig. 2, Supplementary Fig. S13 in S6 and Table 1) evidenced decreases (between − 7% and − 23%) in the Adriatic and Eastern sub-regions basin and in the bathyal areas of the Ionian Sea (− 57%), while an increase was observed only in the open and deeper waters of the Western Mediterranean Sea (+ 286%) and the Eastern-Levantine seas (40%). Discards (Di; Supplementary Fig. S13 in S6 and Table 1) followed a similar pattern but with increases also along the coastal areas of the basin. Small declines (between − 6% and 8%) of trophic level of the catch were observed in the whole basin, mostly in the Adriatic and Eastern-Levantine Sea, and in deeper offshore waters (TLC in Fig. 2 and Table 1).

## Discussion

A spatial–temporal EwE model has been developed for the Mediterranean Sea for the period 1995–2016 to identify spatial patterns and mean temporal changes of marine biodiversity, community biomasses and trophic indices in the region with the overarching goal to assist spatial management actions (e.g., by prioritizing specific areas of concern or specific environmental targets), and facilitate the communication between scientists and policy makers in the future. This study constitutes the first attempt to build a coupled spatial–temporal hydrodynamic-biogeochemical and full food-web model for the whole Mediterranean Sea, using multiple trophic levels (spanning from phytoplankton to marine mammals) over a long time-span (decades) and spatial scale (~ 8 × 8 km).

The model confirms the importance of coasts and shelves in supporting high marine biodiversity, total biomass of commercial and non-commercial species and important ecosystem services like fisheries in the Mediterranean Sea^27^. Coastal and shelf areas, due to high levels of primary production and specific physical (e.g., depth) and environmental (e.g., temperature, salinity) properties^31,32^, are sites where most of the marine biodiversity and species biomass are confined and where most of human interest and exploitation reside^33^.

Our model shows that the Western Mediterranean Sea is the area with the highest total biomass and trophic level of the communities (TL_co_), mostly due to high levels of production; decreasing gradients of biomass were instead observed towards the east side (which is mostly oligotrophic) of the basin, following the west to east pattern, as previously reported by other studies^27,34,35^. Once again, these patterns confirm the important role of bottom-up processes (primary and secondary production) in driving biomass distribution of HTL organisms in the Mediterranean Sea^36–38^.

The Adriatic Sea (mainly the central part) showed less total biomass and TL_co_ than expected. This might be associated with specific physical and chemical features, e.g., shallower waters toward the north, fresh water influence, high variations in temperature and salinity and restricted exchange with the Western basin^31^ and also to the long exploitation of the basin by humans^39,40^.

Another plausible reason for these results could be associated with some pitfalls of the hydrodynamic-biogeochemical model in predicting primary productivity. Macias et al.^41^, in fact, when comparing the satellite chlorophyll (chl a) estimates with the same output from GETM-MedERGOM noticed some discrepancies. In particular, in the Adriatic Sea (mainly the Italian side), the North Aegean Sea, part of the Tunisian coast and around the Nile’s estuary, the model underestimated the amount of chl a in the water in comparison to remote sensing data contrarily, anoverestimation was found along the coastal/shelves areas of some other regions. Such under- or overestimation of production was possibly due to an inappropriate consideration of riverine loads in the model or to an overestimation of chlorophyll values by remote sensing in these very coastal areas^41^ and can propagate through the food web with consequences on species/functional groups distribution and abundance.

This is also probably one of the reasons why the correlation test performed for European hake did not show overall good results. Other possible explanations might be due to the fact that: (1) the survey data of the MEDITS campaign are collected during the summer months^42^, while model outputs are annual, and the sampling months are not consistent across years and/or member States (MS); (2) the model has a coarser resolution (~ 8 × 8 km) compared to the observations (Supplementary Fig. S5 in S6), which might reduce the possibility to capture full species dynamics, particularly along the coast^14^; and (3) the globally averaged AquaMaps environmental response curves are not always representative for the Mediterranean Sea, which could lead to errors in predicted spatial distributions^27^. Other projects, that focused on specific Mediterranean sub-areas, have corrected AquaMaps response curves with the aid of local experts or specific models^26,43^ which is an undertaking that should be repeated for the entire Mediterranean basin in future work. Additionally, for many regions and species in the region, there are not enough spatial–temporal survey data to validate our model with. Therefore, more tests and analyses should be conducted to reduce such uncertainties when data is available at a regional scale.

Mixed correlations were also observed for trawlers and purse seiners effort. This might be related to the lower productivity hindcasted by the GETM-MedERGOM model, which reduces the feeding attractiveness to marine animals targeted by these fishing gears. Another factor that may contribute to lower the correlation is that our model does not explicitly discern between small and large fishing vessels. Meanwhile, AIS data captures mainly larger vessels (52–85% coverage for vessels larger than 24 m; 14–19% percent for vessels between 12 and 24 m and less than 1% for vessels under 12 m; GBW, 2020), excluding therefore the majority of small vessels, which are very abundant in the Mediterranean Sea^44^. While a few studies exist in the literature on the spatial dynamics of trawlers in the region^45,46^, much less effort has been developed to capture the dynamics of the other gears particularly of small-scale fisheries.

As for catches and discards, the Western Mediterranean and the Adriatic Seas were the two sub-regions with the highest level of marine resources exploitation and removals, particularly along the coasts and the continental shelf. The patterns observed in this study are consistent with previous studies^36,38,47,48^ that identified these areas as sites where human activities, especially fisheries, should be better managed due to excessive fishing pressure and/or overexploitation of the marine resources in the region. Trophic level of the catches evidenced catches of higher TL (above 3.5) species (e.g., tunas) in offshore waters and catches with a lower TL (below 3.5) along the coasts and the continental shelf. Since the quantification and distribution of offshore pelagic species biomass and the associated fishing pressure at sea are still difficult to monitor and assess^49^, modelling outputs like ours may become valuable in increasing overall knowledge and in assessing possible spatial management scenarios of these water bodies e.g., through the protection/management of aggregation sites and migration corridors^50^.

As shown in previous studies^30,38^, the Mediterranean biodiversity and its marine resources continue to show signs of deterioration. In particular, coasts (except if close to estuaries) and shelves seem to be the most negatively affected areas by changes in climate (e.g., temperature, salinity), environment (e.g., nutrients, primary production) and fishing pressure as compared to offshore and deeper waters. Coastal areas, being at the interface between land and sea, are subjected to a variety of anthropogenic pressures, e.g., eutrophication, fishing pressure, pollutants^3,4,51^ that, if acting synergistically, may worsen the decrease in marine resources/biodiversity due to the spatial heterogeneity of these ecosystems^52^. Certain estuaries seem to play an important role in maintaining high level of biomass and biodiversity in the surrounding areas. In areas with limited water movements closed to the discharge sites, as the Adriatic/Po and the Aegean/Evros areas, inorganic nutrients from rivers run-off are responsible for a large fraction of the pelagic primary production^53^, possibly impacting also the biomass levels of HTL marine organisms. In the Adriatic Sea, and particularly in areas closed to the Po river, a recent study by Piroddi, et al.^14^ showed, in fact, that nutrients would affect negatively the levels of fish biomass and diversity, if nutrient control measures were implemented to halt eutrophication.

Conversely, patterns of biomass increase in offshore-deeper waters were observed for composite biomass indicators, like the commercial biomass (CB), demersal/pelagic biomass (D/P), and slightly for total biomass (TB). These could be traced back to increases in the biomass of the commercially important bluefin tuna (for CB and TB), and also to increases in biomass of non-commercially important groups like the mesopelagic (for TB) and bathy-demersal fishes (for D/P and TB). Evidence that showed an increase in the biomasses of these functional groups has been observed in other studies, which pointed to changes in environmental conditions and a reduction in fishing effort for bluefin tuna^54,55^, and to environmental conditions, low fishing pressure, removals of predators and/or competitors for mesopelagic and bathy-demersal fishes^56,57^. However, caution should be taken with these results as most of the information (e.g., distribution and biomass) available for these groups at regional and spatial and temporal scales comes from modelling applications. The validation of these outputs is not trivial and more effort should be put in place to reduce this knowledge gap.

Different patterns of biodiversity were observed using the two indices, with the Shannon indicator displaying more reductions in coastal/shelves slope areas in contrast to the Kempton's Q index. The differences observed between the two indices may be due to the fact that in the EwE modelling tool the Shannon index is a measure of group evenness while Kempton tracks changes in both evenness and richness (details in Supplementary Table S5 in S5)^58^. Many indices exist to measure biodiversity, but to date there is no consensus about which indices are most appropriate and informative, so caution should be taken in their selections and the interpretation of results^59^ and a combination of different metrics is suggested^60^.

The increases in offshore catches and discards, mainly in the Western Mediterranean and Eastern Seas, can be associated with an increase in top predators biomass (e.g., bluefin tuna), as previously explained, but also a general movement/distribution of fisheries in offshore waters to counteract the decrease in resources along the coast and shelf areas^61^. This phenomenon has been observed worldwide and also in the Mediterranean Sea, for both industrial and small-scale fisheries^61–63^.

The decline in TLC observed particularly in the Adriatic and the Ionian-Eastern sub-basins agree with recent studies^30,64^ that show a slow decrease rate (~ 0.03) per decade in these areas and pointing at contracted fisheries and shrinking marine food webs as the cause of such decline.

Policy makers often take their decisions having limited and uncertain ecological information, particularly of spatial–temporal data. The biomass distribution of species and communities and even less the ecological processes within and between ecosystems are often not available or sufficiently know to adequately identify and prioritize effective spatial management actions e.g., MPAs implementation^65^, fishing and nutrient control measures^14,66^. Spatial ecosystem modelling tools and their predictions, like the one used in this study, contribute to filling this gap, strengthening the decision making processes under the EBM framework required by several European marine policies. Another important aspect of the usefulness of these type of models for management decisions is based on the engagement and involvement of stakeholders during the development/implementation/scenarios of modelling process to ensure better credibility and utility of these tools^67^. In this context, the coupled models (GETM-MedERGOM-EwE) used in this study, which have been developed at the European Commission by its science Directorate-General (DG), the Joint Research Centre (JRC)^68^, have been designed in collaboration with stakeholders to simulate changes in the state of European marine ecosystems in response to anthropogenic pressures and policy measures, and with the overall goal of providing explicit support to the decision-making process e.g., for the MSFD^13,14^.

## Conclusions

As marine ecosystems are impacted by simultaneous cumulative anthropogenic pressures (e.g., overfishing, aquaculture, human-induced climate change, chemical pollutants/plastics, tourism, shipping/energy), which are expanding apace throughout the world ^3,4,69^, there is an urgent need to evaluate their synergistic effects on the environment^70^ at local and regional scales to better respond and act, with appropriate measures, to fast-changing environments^18^. This study advances the scientific ability to assess the historical cumulative effects of climate change, environment and fisheries at fine spatial scales across the entire Mediterranean basin, and across the entire Mediterranean food web.

Spatial management options, such as MPAs, which currently have a total coverage of only 6% in the whole Mediterranean region^71^, should be considered for management actions and prior to it, their potential effects should be tested, including also the abovementioned multiple pressures, in a scenario analysis using a modelling framework such as the one presented here. As regional ecosystem models are limited in spatial scope, the effort to model the whole Mediterranean Sea with a temporal-hindcast analysis (1995–2016) is an essential asset to investigate current and future ecosystem state of the Mediterranean Sea under the impact of cumulative stressors and to test the outcomes of selected policy scenarios in order to aid local and regional ecosystem-based management.

## Methods

### The initial food web model

**Figure 3:**
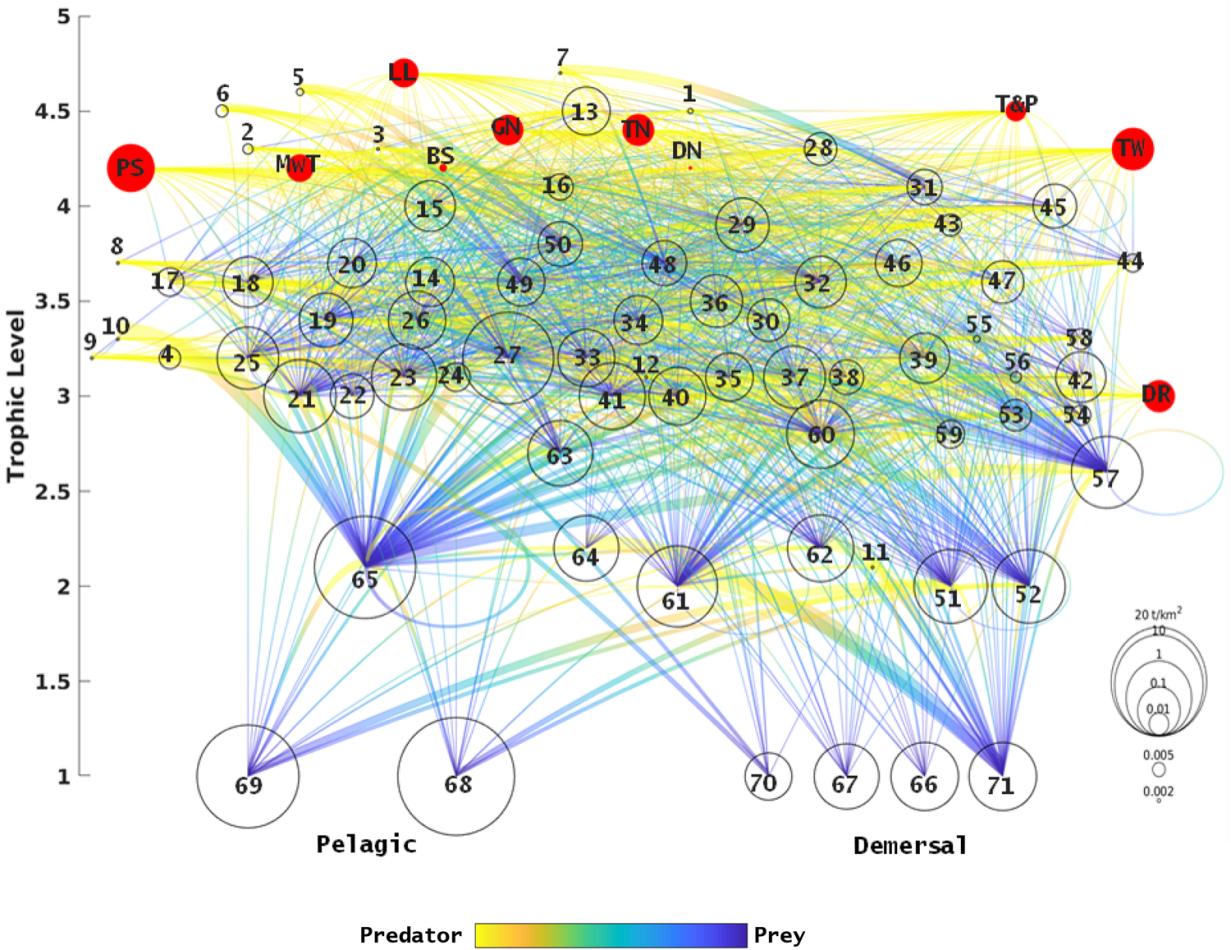
Flow diagram of the Mediterranean Sea ecosystem (1995 period). Each functional group is shown as a bubble and its size is proportional to the log of its biomass. The functional groups are ordered by their trophic levels (y-axis) and their preferred habitat (Pelagic vs Demersal). Predator–prey relationships are expressed with a gradient yellow (predator)-to blue (prey) line. Red bubbles represent fishing fleets (*LL* Longline, *PS* Purse Seine, *MwT* Midwater Trawl, *BS* Beach Seine, *GN* Gillnet, *TN* Trammel net, *DN* Driftnet, *T&P* Traps and pods, *TW* Trawler, *DR* Dredge). Numbers refer to functional groups (Supplementary Table S2 in S2 for the full name list). Map was created by the authors using MATLAB software vR2014b (https://it.mathworks.com/products/matlab/matlab-graphics.html).

**Figure 4:**
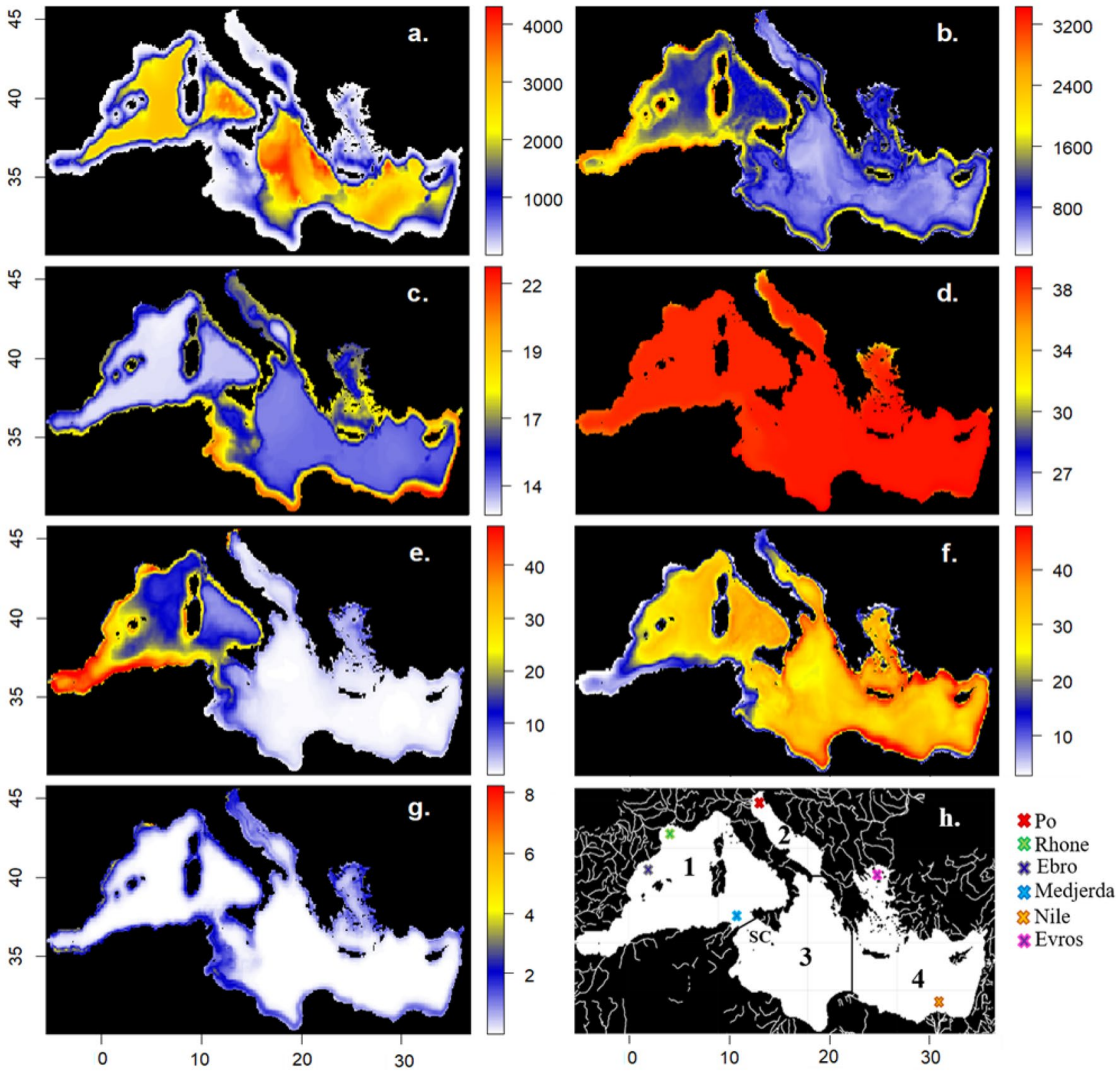
The hydrodynamic-biogeochemical model outputs: (**a**) bathymetry (m); (**b**) primary production (PP, t/km^2^/year); (**c**) temperature (°C); (**d**) salinity (psu); (**e**) large phytoplankton biomass (t/km^2^); (**f**) small phytoplankton biomass (t/km^2^); (**g**) detritus biomass (t/km^2^) used to drive the Mediterranean Sea Ecospace module. Maps 2b–2g are presented as mean for the period 1995–2016 and for the entire water column. Map 4h shows the river loads (white lines and colored crosses) included in GETM-MedERGOM, the Sicilian Channel (SC), and the four main divisions of the Mediterranean Sea accordingly to the European MSFD (Western Mediterranean Sea (1); Adriatic Sea (2); Ionian and Central Mediterranean Sea (3); Aegean and Levantine Sea (4)) to facilitate the visualization of main rivers and areas mentioned in text.

We modified a previously developed EwE food web model coupled with an hydrodynamic-biogeochemical model^38,72^ which represented the entire Mediterranean marine ecosystem and its dynamics for the 1950–2011 period, focusing now on the 1995–2016 period to include the most up-to-date data on e.g., ecological traits, diets, biomasses, fish catches and fishing effort. To best utilize this ecosystem model in support of marine policies, and thanks to the availability of more and better data, we refined the functional groups in the model, originally clustered in bigger groups, either at individual or at family level (e.g., Bottlenose dolphin, *Tursiops truncatus* was pulled out from the group ‘Piscivorous cetaceans’ or Sparidae family from the group ‘Medium demersal’). For highly commercially important species (European pilchard *Sardina pilchardus*, European anchovy *Engraulis encrasicolus*, European hake *Merluccius merluccius*, and Red mullet *Mullus barbatus*), we created multistanza groups by splitting the younger (juvenile individuals) from the older life-stage (adult individuals) and modelling the dynamics of such groups with age-structured models. We also improved the fishing fleet categories, expanding the number of included fishing gears to more accurately represent related catches (landings and discards) and efforts (kW fishing days), using up- to-date United Nation’s Food and Agriculture Organization (FAO) (http://data.fao.org/database) and European Commission (EC) (https://stecf.jrc.ec.europa.eu/reports/dcf-dcr) databases. The new baseline model represents the entire Mediterranean basin for the 1995 period and consists of 71 functional groups, ranging from phytoplankton and invertebrates to top predator species, and up to 10 different types of fishing gears divided in the main Mediterranean MSFD divisions (Western Mediterranean Sea (W); Adriatic Sea (A); Ionian and Central Mediterranean Sea (I); and Aegean Sea and Levantine Sea (E)) (Figs. 3, 4h).

Details on the modelling approach used and the new model set up (input parameters, and references) can be found in S1, S2 and S3 (uploaded separately as "Supplementary Table S3") in the Supporting information. The new model configuration was re-calibrated and validated for the 1995–2016 period. Details on these procedures can be found in S1 and Piroddi et al.^38^.

### Ecospace, the spatial–temporal food web model

The spatial–temporal dynamic module of EwE, Ecospace, represents biomass (B) dynamics of marine species/functional groups over a two-dimensional space grid map^73,74^. The Mediterranean Ecospace model is coupled (one-way; no feedback between the two models) with a three-dimensional hydrodynamic biogeochemical model GETM-MedERGOM^37,41^, having a spatial resolution of 0.083° (8 km) and covering the whole Mediterranean Sea for the same time period (1995–2016). The Ecospace gridded map follows the same spatial resolution as GETM-MedERGOM.

In the spatial model, marine species/functional groups and fleets move between water cells, while cell-specific habitat and environmental attributes determine the cell suitability, or habitat capacity (HCM)^22^. In the HCM, the suitability affects the capacity of species/functional group to forage in each cell and time step based on the assumption that changes in habitat capacity will affect the cell-specific foraging arena available to a given group^22^.

To parameterize the HCM, we linked annual distributions of environmental variable outputs from GETM-MedERGOM to environmental preferences (or “environmental responses”) for specific groups. These outputs include: the bathymetry, yearly averaged salinity and temperature, and yearly integrated primary production (PP) (Fig. 4a–d). As Ecospace does not explicitly consider dynamics in the water column^74^, salinity, temperature and PP were extracted for four different depth ranges: (1) surface (0–10 m); euphotic zone (0–150 m); entire water column (0–2000 m); and bottom (maximum depth) to implicitly incorporate the depth ranges that specific groups utilize, and the influence/importance of related environmental drivers.

Environmental responses for select functional groups were obtained from AquaMaps (www.aquamaps.org)^75^, and were applied either using a trapezoidal shape (e.g., for the bathymetry) to reflect tolerance ranges, or were corrected to binomial shapes (e.g., for temperature) to reflect optimum preferred responses^76^ (Supplementary Fig. S2–S4 in S4). The model further incorporated dispersal rates of organisms and other behavioural parameters; details are provided in Supplementary Table S4 in S4.

Fisheries were distributed according to a fishing effort gravity model^74,77^, in which bottom trawlers are restricted to areas with less than 1000 m of depth in order to comply to the fishing ban imposed by General Fisheries Commission for the Mediterranean (GFCM) (Recommendation GFCM/29/2005/118). Ecospace food web was driven with GETM-MedERGOM derived annual biomass distributions of primary producers and detritus (Fig. 4e–g).

### Spatial–temporal food web model derived indicators

Spatial derived indicators, such as composite biomass and catch indices, ecosystem-scale biodiversity, e.g., Shannon's entropy^78^, Kempton's Q^79^, and trophic-based indices (e.g., mean trophic level of landings TLC, and mean trophic level of the community TL_co_) were extracted from Ecospace outputs using the EcoInd plug-in^80^ and were analysed over space and time. The full list of assessed indices with definition and references is provided in S5, Supplementary Tables S5 and S6. To analyse general patterns and assess overall changes, the yearly modelled indicators (I) are presented as mean for the period 1995–2016 and as the relative mean change, calculated as:

1 $$\left(\frac{{Iyr}_{f}-{Iyr}_{i}}{{Iyr}_{i}}\right)\times 100,$$

where *yr*_*f*_ and *yr*_*i*_ represent the mean of the last and first 5 years respectively.

To evaluate the spatial temporal simulations, the predicted annual mean (1995–2016) biomass of European hake was compared with the annual mean (1995–2016) biomass of the same species caught by the MEDITS trawl survey occurring during spring–summer in many Mediterranean shelf and upper slope areas^42^. European hake was selected because of its commercial importance and its continuous occurrence in the trawl survey across regions and years. Where there were more than one survey data point within a spatial cell (Supplementary Fig. S5 in S6), the mean survey biomass was used. The observed (MEDITS) and the predicted (Ecospace) biomasses were then standardized (difference from the mean divided by the corresponding standard deviation) to facilitate the comparison. The Spearman’s spatial correlations was computed with the raster package function “corLocal” (computes this measure for two spatial objects using a focal neighbourhood) of the R software. Following the same rationale, the modelled mean effort (2015–2016) for bottom trawlers and purse seiners were compared with the available mean effort (2015–2016) for the same fishing fleets from the automatic identification system (AIS, using only fishing hours) of the Global Fishing Watch (GFW)^81^.

## Supplementary Information

Supplementary Information 1.

Supplementary Information 2.

## Data Availability

The authors confirm that the data supporting the findings of this study are available within its supplementary materials.
