# Supplementary material for: Modelling the Mediterranean Sea ecosystem at high spatial resolution to inform the ecosystem-based management in the region
Source: Sci Rep. 2022 Nov 16;12:19680. doi: 10.1038/s41598-022-18017-x (PMC9669036; doi:10.1038/s41598-022-18017-x)
Supplement: Supplementary file 2 — Supplementary Information 2. [file 41598_2022_18017_MOESM2_ESM.docx]

**Modelling the Mediterranean Sea ecosystem at high spatial resolution to inform the ecosystem-based management in the region**

**Chiara Piroddi, Marta Coll, Diego Macias Moy, Jeroen Steenbeek, Elisa Garcia-Gorriz, Alessandro Mannini, Daniel Vilas, Villy Christensen**

**S1.**

**Ecopath baseline model**

Ecopath is a mass-balanced model based on the assumption that the production of one functional group is equal to the sum of all predation, non-predatory losses, exports, biomass accumulations, and catches, as expressed by the following equation:

$$P/{B_{i}}\cdot B_{i}=P/{B_{i}}\cdot B_{i}\cdot\left( 1-{EE}_{i} \right)+\sum_{j} ({Q/B)}_{ji}\cdot B_{i}\cdot{DC}_{ji}+Y_{i}+{NM}_{i}+{BA}_{i}$$

where *B_i_* is the biomass, (*P/B)_i_* is the production rate, (*Q/B)_i_* is the consumption rate, *DC_ji_* is the fraction of prey *i* included in the diet of predator *j*, *NM_i_* is the net migration of prey *i*, *BA_i_* is the biomass accumulation of prey *i*, *Y_i_* is the catch of prey *i*, and *EE_i_* is the ecotrophic efficiency of prey *i*, that is, the proportion of production used in the system.

In an Ecopath model, the energy input and output of all functional groups must be balanced under some ecological and thermodynamic rules: (1) EE < 1.0; (2) P/Q [production/consumption rate or gross efficiency (GE)] ranges from 0.1 to 0.3 with the exception of fast-growing groups such as bacteria; (3) R/A (respiration/food assimilation) < 1; (4) R/B (respiration/biomass) ranges from 1 to 10 for fishes and higher values for small organisms; (5) NE (net efficiency of food conversion) > GE and (6) P/R (production/respiration) < 1 ^1,2^. To balance the Mediterranean model, we applied a manual mass-balanced procedure following a top-down approach modifying appropriate input parameters (starting from the functional groups with higher trophic levels) and following the best practice guidelines provided in the literature ^2-4^. Appendix S2 shows the balanced model and its main input parameters.

**Ecosim, temporal model**

Ecosim model describes the temporal dynamics of species biomass and flows over time by accounting for changes in predation, consumption rate, fishing, and the environment ^1,5^. Ecosim uses a set of differential equations to describe biomass dynamics:

$$\frac{dB_{i}}{dt}=\left( \frac{P}{Q} \right)_{i}\cdot\sum Q_{ji}-\sum Q_{ji}+I_{i}-\left( M_{i}+F_{i}-e_{i} \right)\cdot B_{i}$$

where *dB_i_/dt* is the growth rate of group *i* during time *t* in terms of its biomass *B_i_*; (P/Q)_i_ is the net growth efficiency of group *i*; *Q_ij_* is the consumption rate; *M_i_* is the non-predation mortality rate; *F_i_* is the fishing mortality rate; *e_i_* is the emigration; and *I_i_* is the immigration rate ^1^.

Consumption rates (*Q_ij_*) in Ecosim are calculated based on the “foraging arena” theory, which divides the biomass of the prey into a vulnerable and a non-vulnerable fraction and the transfer rate or vulnerability between the two fractions determines the trophic flow between the predator and the prey [6]. The vulnerability concept incorporates density-dependence processes and expresses how far a group is from its carrying capacity ^1^. Default values of vulnerability (v = 2) represent a mixed trophic flow, a low value (v < 2) indicates ‘bottom-up’ flow control and a situation closer to carrying capacity, while a high value (v > 2) indicates ‘top-down’ flow control and a situation further away from carrying capacity ^6^. For each predator-prey interaction, consumption rates are calculated as:

$Q_{ij}=\frac{a_{ij}\cdot v_{ij}\cdot B_{i}\cdot B_{j}\cdot T_{i}\cdot T_{j}\cdot{M_{ij}}/{D_{j}}}{v_{ij}+v_{ij}\cdot T_{i}\cdot M_{ij}+a_{ij}\cdot M_{ij}\cdot B_{j}\cdot{T_{j}}/{D_{j}}}\cdot f({Env}_{function},t)$,

where *a_ij_* is the rate of effective search for *i* by *j*; *T_i_* represents prey relative feeding time; *T_j_* the predator relative feeding time; *M_ij_* is the mediation forcing effects; *v_ij_* is the vulnerability parameter; *D_j_* represents the effects of handling time as a limit to consumption rate; and *f(Env_function_,t)* is the environmental response function that restricts the size of the foraging arena (*C_rcj_*) to account for external environmental drivers changing over time, such as temperature ^6,7^.

The Ecosim approach was used here to fit the model to observed time-series of data using the sum of squares (SS) ratio between predicted and observed data (survey biomasses and catches for those functional groups with available information) as a metric for assessing model performance. Fishing effort, primary production (PP) of large and small phytoplankton groups and temperature over time were used as main forcing time series to drive the model.

The fitting procedure followed same approach as the one described and applied by Mackinson ^8^. This method uses the Akaike Information criterion (AIC) ^9,10^:

*AIC = nlog (minSS/n) + 2k*

where n is number of observations, minSS is the minimum sum of squares resulting from the comparison of predicted with observed datasets, and k is the number of parameters, to test statistical hypotheses related to changes in predator-prey dynamics; changes in productivity; impact of fishing and possible combinations of the above-mentioned factors. The AIC is a tool used for model selection that penalizes for fitting too many parameters, and which is used to choose the “best” model (the one yielding the lowest AIC) considering a good fit and the least number of estimated parameters to do so. In this study, we used the second-order Akaike Information Criterion (AICc) calculated as follow:

*AICc = AIC + 2k(k-1)/(n-k-1)*

to account for small sample sizes (n of observations) in the dataset. The most statistically significant results in our model fitting exercise were obtained when trophic interactions, fishing and environmental changes (PP and temperature) were included together in the model run (Table 1).

**Table S1**. Results of the temporally dynamic fitting procedure in Ecosim. Years= number of years in the model; n= number of observation; minSS= minimum sum of squares; K= number of parameters; AICc= second-order Akaike Information Criterion; %IF= improved fit compared to the baseline.

| **AIC test** | **no. of years** | **n (number of observations)** | **K** | **minSS** | **AICc** | **%IF** |
| --- | --- | --- | --- | --- | --- | --- |
| Baseline | 22 | 1804 | 0 | 883.65 | -1287.5 |  |
| Fishing + Trophic interactions + Environment | 22 | 1804 | 43 | 605.00 | -1882.9 | **46.24** |

After the temporal dynamic fitting procedure, with the “best” fitted model we run the Monte Carlo routine built into EwE to assess sensitivity of Ecosim’s output to the basic Ecopath input parameters (B, P/B, Q/B, EE, diet). This routine draw input parameters from a normal distribution centered on the base Ecopath value and using a defined coefficient of variation, in this case set to 0.1 ^4^. Here, we run 1000 iterations, and the range of outputs (the 5th and 95th percentile) were plotted for the fitted results (in our case time series of biomasses). Figure 1 shows the modelling fitting results for the functional groups biomasses of the Mediterranean Sea for the 1995-2016 period.


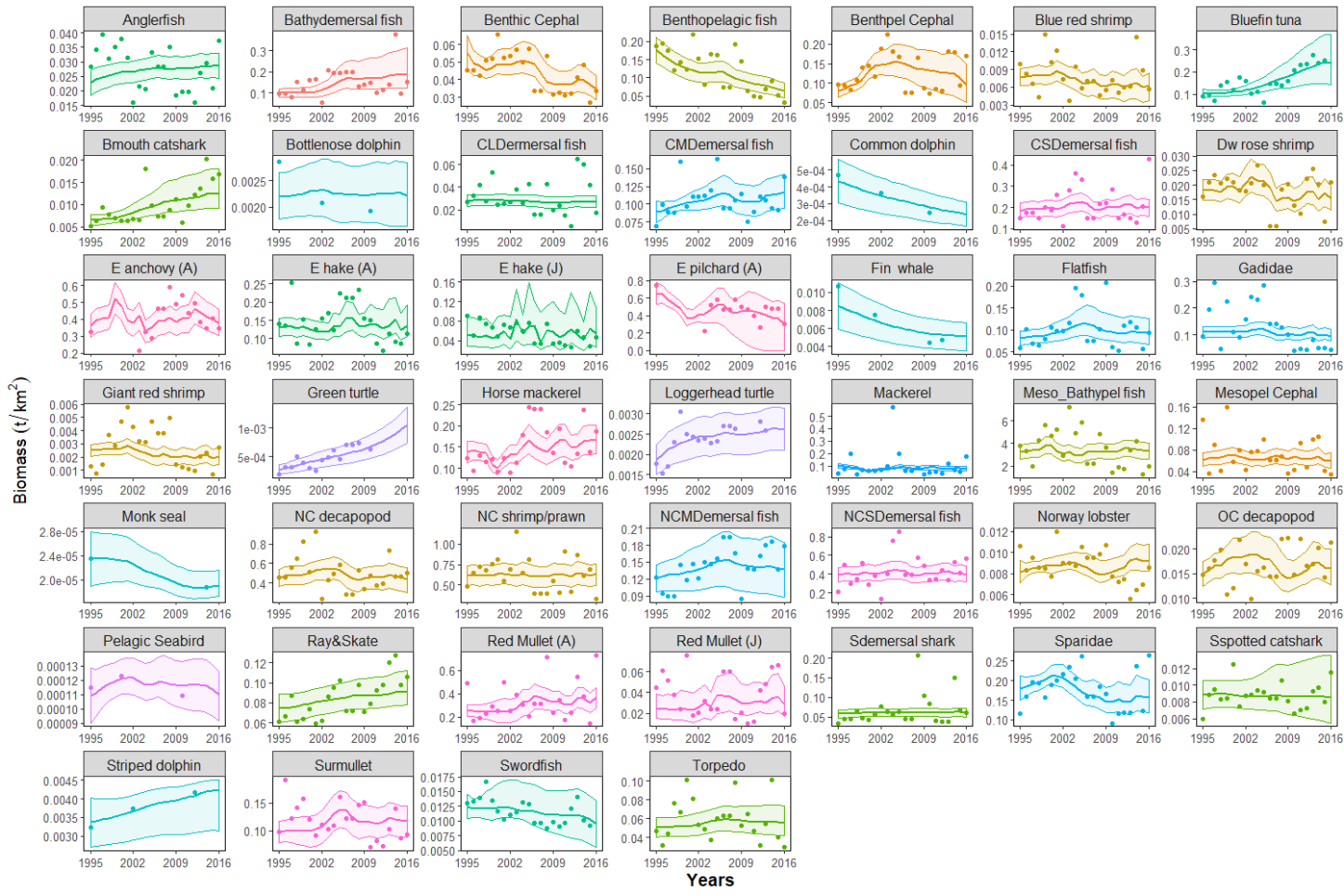


**Fig S1.** Representation of modelling fitting results for some functional groups occurring in the Mediterranean Sea for the period 1995–2016.

**References**

1 Christensen, V. & Walters, C. J. Ecopath with Ecosim: methods, capabilities and limitations. *Ecological modelling* **172**, 109-139 (2004).

2 Heymans, J. J. *et al.* Best practice in Ecopath with Ecosim food-web models for ecosystem-based management. *Ecological Modelling* **331**, 173-184 (2016).

3 Link, J. S. Adding rigor to ecological network models by evaluating a set of pre-balance diagnostics: a plea for PREBAL. *Ecological Modelling* **221**, 1580-1591 (2010).

4 Piroddi, C. *et al.* Historical changes of the Mediterranean Sea ecosystem: modelling the role and impact of primary productivity and fisheries changes over time. *Scientific reports* **7**, 44491 (2017).

5 Walters, C., Christensen, V. & Pauly, D. Structuring dynamic models of exploited ecosystems from trophic mass-balance assessments. *Reviews in fish biology and fisheries* **7**, 139-172 (1997).

6 Ahrens, R. N., Walters, C. J. & Christensen, V. Foraging arena theory. *Fish and Fisheries* **13**, 41-59 (2012).

7 Christensen, V. *et al.* Representing variable habitat quality in a spatial food web model. *Ecosystems* **17**, 1397-1412 (2014).

8 Mackinson, S. Combined analyses reveal environmentally driven changes in the North Sea ecosystem and raise questions regarding what makes an ecosystem model’s performance credible? *Canadian Journal of Fisheries and Aquatic Sciences* **71**, 31-46 (2014).

9 Akaike, H. A new look at the statistical model identification. *Automatic Control, IEEE Transactions on* **19**, 716-723 (1974).

10 Burnham, K. P. & Anderson, D. R. *Model selection and multimodel inference: a practical information-theoretic approach*. (Springer Science & Business Media, 2003).

**Modelling the Mediterranean Sea ecosystem at high spatial resolution to inform the ecosystem-based management in the region**

**Chiara Piroddi, Marta Coll, Diego Macias Moy, Jeroen Steenbeek, Elisa Garcia-Gorriz, Alessandro Mannini, Daniel Vilas, Villy Christensen**

**S2.**

| **Table S.2** Mediterranean functional groups species composition, methods, and references used to estimate the basic input parameters (Biomass (B), Production over Biomass (P/B), Consumption over Biomass (Q/B), Diet (D), By-Catch (B-C), Catch (C), Discard (Di) of the 1990s Ecopath model. Biomass references include also time series data incorporated in Ecosim (1990-2014) module. Abbreviations: E (energy required); W (weight); a (coefficient); z (total mortality); F (fishing mortality); M (natural mortality); T (water temperature); A (aspect ratio caudal fin); h (dummy variable= 1 for herbivores and 0 for carnivores and detritivores); d (dummy variable= 1 for detritivores and 0 for herbivores and carnivores); K (growth coefficient); BT (bottom temperature); D (water depth); Me (dummy variable= 1 for motile epifauna and 0 for others); Car (dummy variable= 1 for carnivorous and 0 for others); Mol (dummy variable= 1 for mollusca and 0 for others); Crus (dummy variable= 1 for crustaceans and 0 for others); Pol (dummy variable= 1 for polychaeta and 0 for others); Echi (dummy variable= 1 for echinodermata and 0 for others) | | |
| --- | --- | --- |
| **Input parameters** | **Method** | **Source** |
| **1. Bottlenose dolphin:** *Tursiops truncatus* | | |
| **B** |  | 1-27 |
| **P/B** | Life history table | 28 |
| **Q/B** | From modified energy requirement equation: E =aW^0.714^ | 29-33 |
| **D** |  | 33-37 |
| **B-C** |  | 38-46 |
| **2. Striped dolphin:** *Stenella coeruleoalba* | | |
| **B** |  | 6,8,10,18,20,21,27,47-57 |
| **P/B** | Life history table | 28 |
| **Q/B** | From modified energy requirement equation: E =aW^0.714^ | 29-31,58,59 |
| **D** |  | 34,37,50,60-66 |
| **B-C** |  | 38-43,67-70 |
| **3. Short-beaked common dolphin:** *Delphinus delphis* | | |
| **B** |  | 6,21,27,52,71,72 |
| **P/B** | Life history table | 28 |
| **Q/B** | From modified energy requirement equation: E =aW^0.714^ | 29-31,73 |
| **D** |  | 37,71,74,75 |
| **B-C** |  | 38-42,68,69 |
| **4.** **Fin whale:** *Balaenoptera physalus* | | |
| **B** |  | 10,47,49-51,56,76-82 |
| **P/B** | Life history table | 28 |
| **Q/B** | From modified energy requirement equation: E =aW^0.714^ | 29-31 |
| **D** |  | 50,83-86 |
| **B-C** |  | 38-41 |
| **5.** **Sperm whale:** *Physeter macrocephalus* | | |
| **B** |  | 10,50,87-94 |
| **P/B** | Life history table | 28 |
| **Q/B** | From modified energy requirement equation: E =aW^0.714^ | 29-31 |
| **D** |  | 50,93,95-97 |
| **B-C** |  | 38-41,68,98 |
| **6.** **Deep sea-cetacean feeder:** *Globicephala melas, Grampus griseus, Ziphius cavirostris* | | |
| **B** |  | 6,8,10,18,20,50,87,88,99-107 |
| **P/B** | Life history table | 28 |
| **Q/B** | From modified energy requirement equation: E =aW^0.714^ | 29-31 |
| **D** |  | 34,37,50,96,99,108-112 |
| **B-C** |  | 38-41,67,68 |
| **7.** **Monk seal:** *Monachus monachus* | | |
| **B** |  | 113-123 |
| **P/B** | Life history table | 28 |
| **Q/B** | From modified energy requirement equation: E =aW^0.75^ | 29,30,124 |
| **D** |  | 125-128 |
| **B-C** |  | 38,40,41,129 |
| **8.** **Endangered and pelagic seabird:** *Calonectris diomedea, Hydrobates pelagicus melitensis, Puffinus yelkouan, Puffinus mauretanicus* | | |
| **B** |  | 130-161 |
| **P/B** | Mortality (z)= -ln(Survival rate) | 162 |
| **Q/B** | Yearly food intake (including breeding and no breeding seasons)/B | 163,164 |
| **D** |  | 130,136,138,155,158,159,165-168 |
| **B-C** |  | 38,41,140,142,143,153,169,170 |
| **9.** **Gull and cormorant:** *Larus audouinii, Larus genei, Larus melanocephalus, Larus michahellis, Larus ridibundus, Phalacrocorax aristotelis, Phalacrocorax carbo* | | |
| **B** |  | 130,134,141,142,146-148,151-156,158,160,161,168,171-206 |
| **P/B** | Mortality (z)= -ln(Survival rate) | 162,207,208 |
| **Q/B** | Yearly food intake (including breeding and no breeding seasons)/B | 163,164 |
| **D** |  | 130,146,155,158,168,173,197,202,209-216 |
| **B-C** |  | 38,41,142,153,170 |
| **10.** **Tern:** *Sterna albifrons, Sterna caspia, Sterna hirundo, Sterna nilotica, Sterna sandvicensis* | | |
| **B** |  | 130,146-148,151,154,156,158,160,180,188-190,198,199,201,205,206,217-222 |
| **P/B** | Mortality (z)= -ln(Survival rate) | 162 |
| **Q/B** | Yearly food intake (including breeding and no breeding seasons)/B | 163,164 |
| **D** |  | 130,155,168,217 |
| **B-C** |  | 38,41,153 |
| **11.** **Loggerhead turtle:** *Caretta caretta* | | |
| **B** |  | 223-238 |
| **P/B** | Mortality (z)= -ln(Survival rate) | 162,236,239 |
| **Q/B** | Yearly food intake/B | 240-242 |
| **D** |  | 243-245 |
| **B-C** |  | 41,67,69,70,236,246-251 |
| **12.** **Green turtle:** *Chelonia mydas* | | |
| **B** |  | 223,224,230-232,236,252-254 |
| **P/B** | Mortality (z)= -ln(Survival rate) | 162,236 |
| **Q/B** | Yearly food intake/B | 241,242,255,256 |
| **D** |  | 255,257 |
| **B-C** |  | 41,236,249,251 |
| **13.** **Pelagic shark:** *Alopias vulpinus, Carcharhinus plumbeus, Carcharia taurus, Carcharodon carcharias, Galeorhinus galeus, Isurus oxyrinchus, Lamna nasus, Prionace glauca* | | |
| **B** |  | International Bottom Trawl Survey in the Mediterranean (Medits), IUCN Red List, 258,259-262 |
| **P/B** | Mortality (z)= F+M | 263 |
| **Q/B** | log (𝑄/𝐵) = 7.964 − 0.204 𝑙𝑜𝑔𝑊 − 1.965 𝑇 + 0.083 𝐴 + 0.532 ℎ + 0.398 𝑑 | 264 |
| **D** |  | 243,265-267 |
| **B-C** |  | 67,69,70,246,268-270 |
| **14.** **Non-commercial large pelagic fish:** *Cetorhinus maximus, Mobula mobular, Mola mola* | | |
| **B** |  | 271-274 |
| **P/B** | Mortality (z)= F+M | 263 |
| **Q/B** | log (𝑄/𝐵) = 7.964 − 0.204 𝑙𝑜𝑔𝑊 − 1.965 𝑇 + 0.083 𝐴 + 0.532 ℎ + 0.398 𝑑 | 264 |
| **D** |  | 243,266,275 |
| **B-C** |  | IUCN Red List, 70,246,270,276,277 |
| **15.** **Bluefin tuna:** *Thunnus thynnus* | | |
| **B** |  | 278 |
| **P/B** | Mortality (z)= F+M | 263 |
| **Q/B** | log (𝑄/𝐵) = 7.964 − 0.204 𝑙𝑜𝑔𝑊 − 1.965 𝑇 + 0.083 𝐴 + 0.532 ℎ + 0.398 𝑑 | 264 |
| **D** |  | 243,279-286 |
| **C** |  | GFCM-FAO |
| **Di** |  | 67,70,247,251,270,287,288 |
| **16. Swordfish:** Xiphias gladius | | |
| **B** |  | 289 |
| **P/B** | Mortality (z)= F+M | 263 |
| **Q/B** | log (𝑄/𝐵) = 7.964 − 0.204 𝑙𝑜𝑔𝑊 − 1.965 𝑇 + 0.083 𝐴 + 0.532 ℎ + 0.398 𝑑 | 264 |
| **D** |  | 243,282,290-294 |
| **C** |  | GFCM-FAO |
| **Di** |  | 67,70,246,247,251,287,288 |
| **17.** **Other large pelagic fish:** *Coryphaena hippurus, Lichia amia, Seriola dumerili, Thunnus alalunga, Regalecus glesne* | | |
| **B** |  | International Bottom Trawl Survey in the Mediterranean (Medits); 295,296,297 |
| **P/B** | Mortality (z)= F+M | 263 |
| **Q/B** | log (𝑄/𝐵) = 7.964 − 0.204 𝑙𝑜𝑔𝑊 − 1.965 𝑇 + 0.083 𝐴 + 0.532 ℎ + 0.398 𝑑 | 264 |
| **D** |  | 243,298-305 |
| **C** |  | GFCM-FAO |
| **Di** |  | 70,251,287,288,306 |
| **18.** **Mackerel:** *Scomber scombrus, Scomber colias, Scomberesox saurus saurus, Scomber spp.* | | |
| **B** |  | International Bottom Trawl Survey in the Mediterranean (Medits); 307,308 |
| **P/B** | Mortality (z)= F+M | 263 |
| **Q/B** | log (𝑄/𝐵) = 7.964 − 0.204 𝑙𝑜𝑔𝑊 − 1.965 𝑇 + 0.083 𝐴 + 0.532 ℎ + 0.398 𝑑 | 264 |
| **D** |  | 243,309-311 |
| **C** |  | GFCM-FAO |
| **Di** |  | DCF (EC), 251,277,287,288,306,312-318 |
| **19.** **Horse mackerel:** *Trachurus trachurus, Trachurus mediterraneus, Trachurus picturatus* | | |
| **B** |  | International Bottom Trawl Survey in the Mediterranean (Medits) |
| **P/B** | Mortality (z)= F+M | 263 |
| **Q/B** | log (𝑄/𝐵) = 7.964 − 0.204 𝑙𝑜𝑔𝑊 − 1.965 𝑇 + 0.083 𝐴 + 0.532 ℎ + 0.398 𝑑 | 264 |
| **D** |  | 243,309,310,319-322 |
| **C** |  | GFCM-FAO |
| **Di** |  | DCF (EC), 251,277,287,288,306,312-316,318,323-330 |
| **20.** **Other medium pelagic fish:** *Alosa alosa, Alosa fallax, Caranx rhonchus, Naucrates doctor, Pomatomus saltatrix, Sarda sarda, Schedophilus medusophagus, Sphyraena sphyraena* | | |
| **B** |  | International Bottom Trawl Survey in the Mediterranean (Medits) |
| **P/B** | Mortality (z)= F+M | 263 |
| **Q/B** | log (𝑄/𝐵) = 7.964 − 0.204 𝑙𝑜𝑔𝑊 − 1.965 𝑇 + 0.083 𝐴 + 0.532 ℎ + 0.398 𝑑 | 264 |
| **D** |  | 310,331-338 |
| **C** |  | GFCM-FAO |
| **Di** |  | DCF (EC), 70,247,251,287,288,306,312-314,316,325,327,328,339,340 |
| **21.** **European pilchard:** *Sardina pilchardus* (Adults&Juveniles) | | |
| **B** |  | 341-356 |
| **P/B** | Mortality (z)= F+M | 263 |
| **Q/B** | log (𝑄/𝐵) = 7.964 − 0.204 𝑙𝑜𝑔𝑊 − 1.965 𝑇 + 0.083 𝐴 + 0.532 ℎ + 0.398 𝑑 | 264 |
| **D** |  | 310,357-364 |
| **C** |  | GFCM-FAO |
| **Di** |  | DCF (EC), 306,312-316,318,323,324,339,365 |
| **23.** **European anchovy:** *Engraulis encrasicolus* (Adults&Juveniles) | | |
| **B** |  | 341-343,345-356,366 |
| **P/B** | Mortality (z)= F+M | 263 |
| **Q/B** | log (𝑄/𝐵) = 7.964 − 0.204 𝑙𝑜𝑔𝑊 − 1.965 𝑇 + 0.083 𝐴 + 0.532 ℎ + 0.398 𝑑 | 264 |
| **D** |  | 357,361,362,364,367-370 |
| **C** |  | GFCM-FAO |
| **Di** |  | DCF (EC), 306,312,313-316,323,327,339,365 |
| **25.** **Other small pelagic fish:** *Cubiceps gracilis, Dussumieria elopsoides, Sardinella aurita, Sardinella maderensis, Syngnathus phlegon, Spicara flexuosa, Spicara maena, Spicara smaris, Sprattus sprattus* | | |
| **B** |  | International Bottom Trawl Survey in the Mediterranean (Medits) |
| **P/B** | Mortality (z)= F+M | 263 |
| **Q/B** | log (𝑄/𝐵) = 7.964 − 0.204 𝑙𝑜𝑔𝑊 − 1.965 𝑇 + 0.083 𝐴 + 0.532 ℎ + 0.398 𝑑 | 264 |
| **D** |  | 310,319,361,362,364,371-376 |
| **C** |  | GFCM-FAO |
| **Di** |  | DCF (EC), 277,306,312,313,315,317,318,323,324,326-330,340,377,378 |
| **26.** **Benthopelagic fish:** *Argyrosomus regius,* *Coelorinchus caelorhincus, Dysomma brevirostre, Eretmophorus kleinenbergi, Gadella maraldi, Gaidropsarus biscayensis, Hoplostethus mediterraneus, Hymenocephalus italicus, Lagocephalus lagocephalus,* *Lagocephalus sceleratus, Lagocephalus suezensis, Lepidopus caudatus, Nezumia aequalis, Nezumia sclerorhynchus, Physiculus dalwigki, Schedophilus ovalis, Sphoeroides pachygaster, Stromateus fiatola, Trichiurus lepturus* | | |
| **B** |  | International Bottom Trawl Survey in the Mediterranean (Medits) |
| **P/B** | Mortality (z)= F+M | 263 |
| **Q/B** | log (𝑄/𝐵) = 7.964 − 0.204 𝑙𝑜𝑔𝑊 − 1.965 𝑇 + 0.083 𝐴 + 0.532 ℎ + 0.398 𝑑 | 264 |
| **D** |  | 243,310,379-386 |
| **C** |  | GFCM-FAO |
| **Di** |  | 306,312,323,324,329,340,377,387-390 |
| **27.** **No commercial meso (bathy) pelagic fish:** *Arctozenus risso,* *Argyropelecus aculeatus, Argyropelecus hemigymnus, Bathophilus nigerrimus, Benthosema glaciale, Brama brama, Centrolophus niger, Ceratoscopelus maderensis, Chauliodus sloani, Cyclothone braueri, Cyclothone pygmaea, Diaphus holti, Diaphus metopoclampus, Diaphus rafinesquii, Electrona risso, Evermannella balbo, Gadiculus argenteus, Gonichthys cocco, Gonostoma denudatum, Hygophum benoiti, Hygophum hygomii, Ichthyococcus ovatus, Lampanyctus crocodilus, Lampanyctus pusillus, Lepidion lepidion, Lestidiops sphyrenoides, Lobianchia dofleini, Lobianchia gemellarii, Maurolicus muelleri, Melanostigma atlanticum, Mora moro, Myctophidae, Myctophum punctatum, Nansenia oblita, Nemichthys scolopaceus, Nettastoma melanurum, Notacanthus bonaparte, Notoscopelus bolini, Notoscopelus elongatus, Paralepis coregonoides, Psenes pellucidus, Rhynchogadus hepaticus, Stomias boa boa, Sudis hyalina, Symbolophorus veranyi, Trachipterus trachypterus, Vinciguerria attenuata, Vinciguerria poweriae* | | |
| **B** |  | International Bottom Trawl Survey in the Mediterranean (Medits) |
| **P/B** | Mortality (z)= F+M | 263 |
| **Q/B** | log (𝑄/𝐵) = 7.964 − 0.204 𝑙𝑜𝑔𝑊 − 1.965 𝑇 + 0.083 𝐴 + 0.532 ℎ + 0.398 𝑑 | 264 |
| **D** |  | 382,391-400 |
| **C** |  | GFCM-FAO |
| **Di** |  | 70,312,323,324,329,340,377,389,401 |
| **28. Anglerfish:** Lophius budegassa, Lophius piscatorius | | |
| **B** |  | International Bottom Trawl Survey in the Mediterranean (Medits) |
| **P/B** | Mortality (z)= F+M | 263 |
| **Q/B** | log (𝑄/𝐵) = 7.964 − 0.204 𝑙𝑜𝑔𝑊 − 1.965 𝑇 + 0.083 𝐴 + 0.532 ℎ + 0.398 𝑑 | 264 |
| **D** |  | 310,382,402-406 |
| **C** |  | GFCM-FAO |
| **Di** |  | DCF (EC), 277,306,313-315,317,323,324,328-330,401 |
| **29.** **European hake:** *Merluccius merluccius* (Adults&Juveniles) | | |
| **B** |  | International Bottom Trawl Survey in the Mediterranean (Medits) |
| **P/B** | Mortality (z)= F+M | 263 |
| **Q/B** | log (𝑄/𝐵) = 7.964 − 0.204 𝑙𝑜𝑔𝑊 − 1.965 𝑇 + 0.083 𝐴 + 0.532 ℎ + 0.398 𝑑 | 264 |
| **D** |  | 310,382,407-414 |
| **C** |  | GFCM-FAO |
| **Di** |  | DCF (EC), 277,306,313-315,323,324,328-330 |
| **31.** **Other commercial large demersal fish:** *Conger conger,* *Epinephelus aeneus, Epinephelus costae, Epinephelus marginatus, Molva dypterygia, Molva macrophthalma, Molva molva, Polyprion americanus* | | |
| **B** |  | International Bottom Trawl Survey in the Mediterranean (Medits) |
| **P/B** | Mortality (z)= F+M | 263 |
| **Q/B** | log (𝑄/𝐵) = 7.964 − 0.204 𝑙𝑜𝑔𝑊 − 1.965 𝑇 + 0.083 𝐴 + 0.532 ℎ + 0.398 𝑑 | 264 |
| **D** |  | 310,382,415-423 |
| **C** |  | GFCM-FAO |
| **Di** |  | DCF (EC), 277,306,312-314,323,324,329,330,389,401,424 |
| **32. Gadidae:** *Merlangius merlangus, Micromesistius poutassou, Trisopterus capelanus, Trisopterus luscus* | | |
| **B** |  | International Bottom Trawl Survey in the Mediterranean (Medits) |
| **P/B** | Mortality (z)= F+M | 263 |
| **Q/B** | log (𝑄/𝐵) = 7.964 − 0.204 𝑙𝑜𝑔𝑊 − 1.965 𝑇 + 0.083 𝐴 + 0.532 ℎ + 0.398 𝑑 | 264 |
| **D** |  | 310,382,425-433 |
| **C** |  | GFCM-FAO |
| **Di** |  | DCF (EC), 277,312-315,323,324,327-330,389,401 |
| **33.** **Sparidae:** *Boops boops, Centracanthus cirrus, Dentex dentex, Dentex gibbosus, Dentex macrophthalmus, Dentex maroccanus, Diplodus annularis, Diplodus cervinus, Diplodus puntazzo, Diplodus sargus, Diplodus vulgaris, Lithognathus mormyrus, Oblada melanura, Pagellus acarne, Pagellus bogaraveo, Pagellus erythrinus, Pagrus caeruleostictus, Pagrus pagrus, Sarpa salpa, Sparus aurata, Spondyliosoma cantharus* | | |
| **B** |  | International Bottom Trawl Survey in the Mediterranean (Medits) |
| **P/B** | Mortality (z)= F+M | 263 |
| **Q/B** | log (𝑄/𝐵) = 7.964 − 0.204 𝑙𝑜𝑔𝑊 − 1.965 𝑇 + 0.083 𝐴 + 0.532 ℎ + 0.398 𝑑 | 264 |
| **D** |  | 310,319,414,419,428,434-463 |
| **C** |  | GFCM-FAO |
| **Di** |  | DCF (EC), 277,306,312-318,323,324,326,329,330,339,401 |
| **34.** **Other commercial medium demersal fish:** *Anguilla anguilla, Chelidonichthys cuculus, Chelidonichthys lucerna, Chelidonichthys obscurus, Chelon labrosus, Dactylopterus volitans, Dicentrarchus labrax, Eutrigla gurnardus, Gaidropsarus mediterraneus, Gaidropsarus vulgaris, Gnathophis mystax, Helicolenus dactylopterus, , Labrus mixtus, Labrus viridis, Liza aurata, Liza ramada, Liza saliens, Mugil cephalus, Oedalechilus labeo, Parapristipoma octolineatum, Peristedion cataphractum, Phycis blennoides, Phycis phycis, Sciaena umbra, Scorpaena elongata, Scorpaena loppei, Scorpaena maderensis, Scorpaena notata, Scorpaena porcus, Scorpaena scrofa, Serranus atricauda, Serranus cabrilla, Serranus hepatus, Serranus scriba, Sparisoma cretense, Symphodus tinca, Trachinus radiatus, Trachyscorpia cristulata echinata, Trigla lyra, Trigloporus lastoviza, Umbrina canariensis, Umbrina cirrosa, Umbrina ronchus, Xyrichtys novacula, Zeus faber* | | |
| **B** |  | International Bottom Trawl Survey in the Mediterranean (Medits) |
| **P/B** | Mortality (z)= F+M | 263 |
| **Q/B** | log (𝑄/𝐵) = 7.964 − 0.204 𝑙𝑜𝑔𝑊 − 1.965 𝑇 + 0.083 𝐴 + 0.532 ℎ + 0.398 𝑑 | 264 |
| **D** |  | 310,319,382,419,438,443,448,461,464-495 |
| **C** |  | GFCM-FAO |
| **Di** |  | DCF (EC), 247,277,306,312,313,315,317,318,323,324,326-330,377,389,390,401,496 |
| **35.** **Flatfish:** *Arnoglossus imperialis, Arnoglossus kessleri, Arnoglossus laterna, Arnoglossus rueppelii, Arnoglossus thori, Bathysolea profundicola, Bothus podas, Buglossidium luteum, Citharus linguatula, Dicologlossa cuneata, Dicologlossa hexophthalma, Lepidorhombus boscii, Lepidorhombus whiffiagonis, Microchirus azevia, Microchirus boscanion, Microchirus ocellatus, Microchirus variegatus, Monochirus hispidus, Platichthys flesus, Pegusa impar, Pegusa lascaris, Scophthalmus maximus, Scophthalmus rhombus, Solea senegalensis, Solea solea, Solea spp., Symphurus ligulatus, Symphurus nigrescens, Synapturichthys kleinii, Zeugopterus regius* | | |
| **B** |  | International Bottom Trawl Survey in the Mediterranean (Medits) |
| **P/B** | Mortality (z)= F+M | 263 |
| **Q/B** | log (𝑄/𝐵) = 7.964 − 0.204 𝑙𝑜𝑔𝑊 − 1.965 𝑇 + 0.083 𝐴 + 0.532 ℎ + 0.398 𝑑 | 264 |
| **D** |  | 310,319,382,406,419,431,448,497-508 |
| **C** |  | GFCM-FAO |
| **Di** |  | DCF (EC), 247,277,306,312,313,323,324,326,327,329,330,339,377,389,401,496 |
| **36.** **No commercial medium demersal fish:** *Apterichtus caecus, Aulopus filamentosus, Balistes capriscus, Cepola macrophthalma, Chlopsis bicolor, Dalophis imberbis, Echelus myrus, Facciolella oxyrhyncha, Fistularia commersonii, Muraena helena, Nerophis maculatus, Ophichthus rufus, Ophidion barbatum, Ophidion rochei, Ophisurus serpens, Saurida undosquamis, Siganus luridus, Siganus rivulatus, Stephanolepis diaspros, Syngnathus acus, Syngnathus spp., Syngnathus tenuirostris, Syngnathus typhle, Synodus saurus, Trachinus araneus, Trachinus draco, Uranoscopus scaber* | | |
| **B** |  | International Bottom Trawl Survey in the Mediterranean (Medits) |
| **P/B** | Mortality (z)= F+M | 263 |
| **Q/B** | log (𝑄/𝐵) = 7.964 − 0.204 𝑙𝑜𝑔𝑊 − 1.965 𝑇 + 0.083 𝐴 + 0.532 ℎ + 0.398 𝑑 | 264 |
| **D** |  | 310,319,419,448,474,487,509-518 |
| **Di** |  | 247,277,306,313,324,326,327,330,377,401,496 |
| **37. Red mullet:** *Mullus barbatus* (Adults&Juveniles) | | |
| **B** |  | International Bottom Trawl Survey in the Mediterranean (Medits) |
| **P/B** | Mortality (z)= F+M | 263 |
| **Q/B** | log (𝑄/𝐵) = 7.964 − 0.204 𝑙𝑜𝑔𝑊 − 1.965 𝑇 + 0.083 𝐴 + 0.532 ℎ + 0.398 𝑑 | 264 |
| **D** |  | 310,448,462,509,519-525 |
| **C** |  | GFCM-FAO |
| **Di** |  | DCF (EC), 306,312,313-315,317,326,330,401 |
| **39. Surmullet:** *Mullus surmuletus* | | |
| **B** |  | International Bottom Trawl Survey in the Mediterranean (Medits) |
| **P/B** | Mortality (z)= F+M | 263 |
| **Q/B** | log (𝑄/𝐵) = 7.964 − 0.204 𝑙𝑜𝑔𝑊 − 1.965 𝑇 + 0.083 𝐴 + 0.532 ℎ + 0.398 𝑑 | 264 |
| **D** |  | 310,319,419,448,462,509,518,519,523,526,527 |
| **C** |  | GFCM-FAO |
| **Di** |  | DCF (EC), 277,306,312,313,315,317,326,377,401 |
| **40. Commercial small demersal fish:** *Acantholabrus palloni,* *Anthias anthias,* *Aphia minuta, Ariosoma balearicum, Atherina boyeri, Coris julis, Gymnammodytes cicerelus, Lappanella fasciata,* *Lepidotrigla cavillone, Lepidotrigla dieuzeidei, Symphodus cinereus, Symphodus doderleini, Symphodus mediterraneus, Symphodus ocellatus, Symphodus roissali, Symphodus rostratus* | | |
| **B** |  | International Bottom Trawl Survey in the Mediterranean (Medits) |
| **P/B** | Mortality (z)= F+M | 263 |
| **Q/B** | log (𝑄/𝐵) = 7.964 − 0.204 𝑙𝑜𝑔𝑊 − 1.965 𝑇 + 0.083 𝐴 + 0.532 ℎ + 0.398 𝑑 | 264 |
| **D** |  | 310,319,419,448,467,474,528-533 |
| **C** |  | GFCM-FAO |
| **Di** |  | DCF (EC), 247,277,306,312-314,317,318,323,327,329,534 |
| **41. No commercial small demersal fish:** *Apogon imberbis, Blenniidae, Blennius ocellaris, Callanthias ruber, Callionymus lyra, Callionymus pusillus, Callionymus spp., Capros aper, Carapus acus, Chlorophthalmus agassizi, Chromis chromis, Coryphoblennius galerita, Crystallogobius linearis, Deltentosteus collonianus, Deltentosteus quadrimaculatus, Diplecogaster bimaculata, Echiodon dentatus, Gobius cobitis, Gobius cruentatus, Gobius fallax, Gobius geniporus, Gobius niger, Gobius paganellus, Gobius spp., Grammonus ater, Hippocampus guttulatus, Hippocampus hippocampus, Hippocampus ramulosus, Lepadogaster lepadogaster, Lepadogaster spp., Lesueurigobius friesii, Lesueurigobius sanzi, Lesueurigobius suerii, Macroramphosus scolopax, Microlipophrys adriaticus, Microichthys coccoi, Parablennius gattorugine, Parablennius incognitus, Parablennius tentacularis, Pomatoschistus marmoratus, Pomatoschistus microps, Pomatoschistus minutus, Pomatoschistus norvegicus, Pomatoschistus spp., Pteragogus pelycus, Salaria pavo, Synchiropus phaeton, Syngnathus abaster, Syngnathus taenionotus, Upeneus moluccensis, Upeneus pori* | | |
| **B** |  | International Bottom Trawl Survey in the Mediterranean (Medits) |
| **P/B** | Mortality (z)= F+M | 263 |
| **Q/B** | log (𝑄/𝐵) = 7.964 − 0.204 𝑙𝑜𝑔𝑊 − 1.965 𝑇 + 0.083 𝐴 + 0.532 ℎ + 0.398 𝑑 | 264 |
| **D** |  | 310,382,419,448,462,474,509,535-542 |
| **Di** |  | 247,306,312,313,318,324,326,327,329,390,534 |
| **42. Bathydemersal (deep sea) fish:** *Alepocephalus rostratus, Argentina sphyraena, Bathypterois dubius, Bellottia apoda, Benthocometes robustus, Borostomias antarcticus, Cataetyx alleni, Coelorinchus occa, Epigonus constanciae, Epigonus denticulatus, Epigonus telescopus, Eutelichthys leptochirus, Glossanodon leioglossus, Paraliparis murieli, Polyacanthonotus rissoanus, Trachyrincus scabrus* | | |
| **B** |  | International Bottom Trawl Survey in the Mediterranean (Medits) |
| **P/B** | Mortality (z)= F+M | 263 |
| **Q/B** | log (𝑄/𝐵) = 7.964 − 0.204 𝑙𝑜𝑔𝑊 − 1.965 𝑇 + 0.083 𝐴 + 0.532 ℎ + 0.398 𝑑 | 264 |
| **D** |  | 317,382,394,395,536,543-546 |
| **C** |  | GFCM-FAO |
| **Di** |  | 312,323,324,329,389 |
| **43. Small-spotted catshark:** *Scyliorhinus canicula* | | |
| **B** |  | International Bottom Trawl Survey in the Mediterranean (Medits) |
| **P/B** | Mortality (z)= F+M | 263 |
| **Q/B** | log (𝑄/𝐵) = 7.964 − 0.204 𝑙𝑜𝑔𝑊 − 1.965 𝑇 + 0.083 𝐴 + 0.532 ℎ + 0.398 𝑑 | 264 |
| **D** |  | 310,382,448,547-552 |
| **C** |  | GFCM-FAO |
| **Di** |  | DCF (EC), 247,277,312,315,317,323,324,329,330,377,389,401 |
| **44. Blackmouth catshark:** *Galeus melastomus* | | |
| **B** |  | International Bottom Trawl Survey in the Mediterranean (Medits) |
| **P/B** | Mortality (z)= F+M | 263 |
| **Q/B** | log (𝑄/𝐵) = 7.964 − 0.204 𝑙𝑜𝑔𝑊 − 1.965 𝑇 + 0.083 𝐴 + 0.532 ℎ + 0.398 𝑑 | 264 |
| **D** |  | 310,382,551-554 |
| **C** |  | GFCM-FAO |
| **Di** |  | DCF (EC), 312,315,317,323,324,329,377,389 |
| **45. Other small demersal sharks:** *Centrophorus granulosus, Chimaera monstrosa, Dalatias licha, Etmopterus spinax, Galeus atlanticus, Heptranchias perlo, Hexanchus griseus, Hiatella arctica, Mustelus asterias, Mustelus mustelus, Mustelus punctulatus, Oxynotus centrina, Scyliorhinus stellaris, Squalus acanthias, Squalus blainville, Squalus uyato, Squatina aculeata, Squatina oculata, Squatina squatina* | | |
| **B** |  | International Bottom Trawl Survey in the Mediterranean (Medits) |
| **P/B** | Mortality (z)= F+M | 263 |
| **Q/B** | log (𝑄/𝐵) = 7.964 − 0.204 𝑙𝑜𝑔𝑊 − 1.965 𝑇 + 0.083 𝐴 + 0.532 ℎ + 0.398 𝑑 | 264 |
| **D** |  | 266,310,382,448,549,551,553,555-566 |
| **C** |  | GFCM-FAO |
| **Di** |  | DCF (EC), 277,306,312,313,315,317,323,324,329,377,389,424 |
| **46. Rays and skates:** *Dasyatis centroura, Dasyatis pastinaca, Dasyatis tortonesei, Dipturus batis, Dipturus nidarosiensis, Dipturus oxyrinchus, Gymnura altavela, Leucoraja circularis, Leucoraja fullonica, Leucoraja melitensis, Leucoraja naevus, Myliobatis aquila, Pteromylaeus bovinus, Pteroplatytrygon violacea, Raja asterias, Raja brachyura, Raja clavata, Raja miraletus, Raja montagui, Raja polystigma, Raja radula, Raja spp., Raja undulata, Rostroraja alba* | | |
| **B** |  | International Bottom Trawl Survey in the Mediterranean (Medits) |
| **P/B** | Mortality (z)= F+M | 263 |
| **Q/B** | log (𝑄/𝐵) = 7.964 − 0.204 𝑙𝑜𝑔𝑊 − 1.965 𝑇 + 0.083 𝐴 + 0.532 ℎ + 0.398 𝑑 | 264 |
| **D** |  | 266,310,406,448,549,551,552,567-579 |
| **C** |  | GFCM-FAO |
| **Di** |  | DCF (EC), 70,247,277,306,312,313,315,317,323,324,329,330,339,377,401,424 |
| **47. Torpedos:** *Torpedo marmorata, Torpedo nobiliana, Torpedo spp., Torpedo torpedo* | | |
| **B** |  | International Bottom Trawl Survey in the Mediterranean (Medits) |
| **P/B** | Mortality (z)= F+M | 263 |
| **Q/B** | log (𝑄/𝐵) = 7.964 − 0.204 𝑙𝑜𝑔𝑊 − 1.965 𝑇 + 0.083 𝐴 + 0.532 ℎ + 0.398 𝑑 | 264 |
| **D** |  | 266,310,448,518,580,581 |
| **C** |  | GFCM-FAO |
| **Di** |  | 247,277,306,314,315,317,324,329,330,401,496 |
| **48. Benthic cephalopods:** *Bathypolypus sponsalis, Callistoctopus macropus, Eledone cirrhosa, Eledone moschata, Macrotritopus defilippi, Neorossia caroli, Octopus salutii, Octopus vulgaris, Pteroctopus tetracirrhus, , Rondeletiola minor, Rossia macrosoma, Rossia spp., Scaeurgus unicirrhus, Sepia elegans, Sepia officinalis, Sepia orbignyana, Sepietta neglecta, Sepietta obscura, Sepietta oweniana, Sepietta spp., Sepiola affinis, Sepiola intermedia, Sepiola ligulata, Sepiola robusta, Sepiola rondeletii, Sepiola spp.* | | |
| **B** |  | International Bottom Trawl Survey in the Mediterranean (Medits) |
| **P/B** | log M =- 0.2107-0.0824 log W +0.6757 log K + 0.4627 log T | 582 |
| **Q/B** |  | 583 |
| **D** |  | 552,584-593 |
| **C** |  | GFCM-FAO |
| **Di** |  | DCF (EC), 277,306,312,313,317,324,326,329,377,389,401,534,552 |
| **49. Benthopelagic cephalopods:** *Abralia veranyi, Abraliopsis morisii, Alloteuthis media, Alloteuthis spp., Alloteuthis subulata, Illex coindetii, Loligo forbesii, Loligo spp., Loligo vulgaris, Ocythoe tuberculata, Opisthoteuthis calypso, Todarodes sagittatus, Todaropsis eblanae* | | |
| **B** |  | International Bottom Trawl Survey in the Mediterranean (Medits) |
| **P/B** | log M =- 0.2107-0.0824 log W+0.6757 log K + 0.4627 log T | 582 |
| **Q/B** |  | 583 |
| **D** |  | 552,594-601 |
| **C** |  | GFCM-FAO |
| **Di** |  | DCF (EC), 277,306,312,313,315,317,318,324,327,329,377,389,401,496 |
| **50.** **Mesopelagic cephalopods:** *Ancistrocheirus lesueurii,* *Ancistroteuthis lichtensteinii, Argonauta argo, Brachioteuthis riisei, Chiroteuthis veranii, Chtenopteryx sicula, Heteroteuthis dispar, Histioteuthis bonnellii, Histioteuthis reversa, Histioteuthis spp., Octopoteuthis sicula, Onychoteuthis banksii, Pyroteuthis margaritifera, Stoloteuthis leucoptera* | | |
| **B** |  | International Bottom Trawl Survey in the Mediterranean (Medits) |
| **P/B** | log M =- 0.2107-0.0824 log W+0.6757 log K + 0.4627 log T | 582 |
| **Q/B** |  | 583 |
| **D** |  | 552,598,602 |
| **Di** |  | 70,312,324,329,389 |
| **51. Bivalves:** *Abra alba, Abra longicallus Acanthocardia aculeata, Acanthocardia echinata, Acanthocardia paucicostata, Acanthocardia spinosa, Acanthocardia tuberculata, Aequipecten commutatus, Aequipecten opercularis, Amygdalum politum, Anadara corbuloides, Anadara inaequivalvis, Anadara transversa, Anomia ephippium, Arca noae, Arca tetragona, Arcopagia balaustina, Arcopagia crassa, Atrina fragilis, Atrina pectinata, Callista chione, Cardiomya spp., Chama gryphoides, Chamelea gallina, Clausinella fasciata, CrassOweniidaea gigas, Cuspidaria cuspidata, Flexopecten flexuosus, Flexopecten glaber glaber, Gibbomodiola adriatica, Glossus humanus, Gryphus vitreus, Hiatella arctica, Laevicardium crassum, Laevicardium oblongum, Lima lima, Limaria hians, Lutraria spp, Mimachlamys varia, Modiolus modiolus, Monia squama, Musculus subpictus, Mytilus edulis, Mytilus galloprovincialis, Neopycnodonte cochlear, Nucula nucleus, Nucula sulcata, Ostrea edulis, Pecten jacobaeus, Pecten maximus, Pinna nobilis, Pinna rudis, Pseudamussium clavatum, Pteria hirundo, Ruditapes decussatus, Solecurtus scopula, Spisula subtruncata, Tellina fabula, Tellina planata, Teredo navalis, Thracia phaseolina, Venus casina, Venus nux, Venus verrucosa* | | |
| **B** |  | International Bottom Trawl Survey in the Mediterranean (Medits) |
| **P/B** | log(P/B)=10.154-0.271*LOG(W)-2824.247*(1/(BT+273)-0.063*LOG(D+1)+0.13*(Me)+0.076*(Car)-0.311*(Mol)-0.154*(Crus)-0.266*(Pol)-0.472*(Echi) | 603,604 |
| **Q/B** | (P/B)/(P/Q) |  |
| **D** |  | 605-608 |
| **C** |  | GFCM-FAO |
| **Di** |  | 312,313,324,329,339,377,534 |
| **52.** **Gastropods:** *Alcyonidium spp., Aplysia depilans, Aplysia fasciata, Aplysia punctata, Aplysia parvula, Aporrhais pespelecani, Aporrhais serresianus, Armina loveni, Armina maculata, Armina neapolitana, Armina tigrina, Berthella aurantiaca, Bivetiella cancellata, Bolinus brandaris, Bolma rugosa, Buccinum humphreysianum, Calliostoma granulatum, Calliostoma laugieri, Calliostoma zizyphinum, Callumbonella suturalis, Calyptraea chinensis, Capulus ungaricus, Cavolinia tridentata, Coralliophila meyendorffii, Crepidula unguiformis, Charonia lampas, Colus gracilis, Colus jeffreysianus, Cylichna cylindracea, Cymbium olla, Cymbulia peronii, Dendrodoris limbata, Diodora italica, Doris pseudoargus, Doris sticta, Doris verrucosa, Epitonium clathratulum, Epitonium clathrus, Euspira catena, Euspira fusca, Euspira grossularia, Euspira guilleminii, Euthria cornea, Felimare picta, Fusinus rostratus, Fusinus syracusanus, Fusiturris similis, Fusiturris undatiruga, Galeodea echinophora, Galeodea rugosa, Gastropteron rubrum, Gibbula magus, Hadriania craticulata, Haminoea hydatis, Haminoea navicula, Hexaplex trunculus, Hirtomurex squamosus, Jorunna tomentosa, Kaloplocamus ramosus, Lamellaria perspicua, Marionia blainvillea, Monoplex corrugatus, Nassarius incrassatus, Nassarius lima, Nassarius reticulatus, Naticarius hebraeus, Naticarius stercusmuscarum, Ocenebra erinaceus, Okenia elegans, Peltodoris atromaculata, Philine aperta, Philinopsis depicta, Platydoris argo, Pleurobranchaea meckeli, Pleurobranchus membranaceus, Pleurobranchus testudinarius, Pseudosimnia adriatica, Pseudosimnia carnea, Ranella olearium, Scaphander lignarius, Semicassis granulata, Semicassis saburon, Tenagodus obtusus, Tethys fimbria, Tonna galea, Tritonia hombergii, Trophonopsis muricata, Turritella communis, Turritella turbona, Umbraculum umbraculum, Xenophora crispa* | | |
| **B** |  | International Bottom Trawl Survey in the Mediterranean (Medits) |
| **P/B** | log(P/B) = 10.154-0.271*LOG(M)-2824.247*(1/(T+273)-0.063*LOG(D+1)+0.13*(Me)+0.076*(Car)-0.311*(Mol)-0.154*(Crus)-0.266*(Pol)-0.472*(Echi) | 603,604 |
| **Q/B** | (P/B)/(P/Q) |  |
| **D** |  | 609-613 |
| **C** |  | GFCM-FAO |
| **Di** |  | 312-314,324,329,339,534 |
| **53. Deep-water rose shrimp:** *Parapenaeus longirostris* | | |
| **B** |  | International Bottom Trawl Survey in the Mediterranean (Medits) |
| **P/B** | log(P/B) = 10.154-0.271*LOG(M)-2824.247*(1/(T+273)-0.063*LOG(D+1)+0.13*(Me)+0.076*(Car)-0.311*(Mol)-0.154*(Crus)-0.266*(Pol)-0.472*(Echi) | 603,604 |
| **Q/B** |  | 583 |
| **D** |  | 614-617 |
| **C** |  | GFCM-FAO |
| **Di** |  | DCF (EC), 306,313,317,323,324,329,377,378,389,534 |
| **54. Blue and red shrimp:** *Aristeus antennatus* | | |
| **B** |  | International Bottom Trawl Survey in the Mediterranean (Medits) |
| **P/B** | log(P/B) = 10.154-0.271*LOG(M)-2824.247*(1/(T+273)-0.063*LOG(D+1)+0.13*(Me)+0.076*(Car)-0.311*(Mol)-0.154*(Crus)-0.266*(Pol)-0.472*(Echi) | 603,604 |
| **Q/B** |  | 583 |
| **D** |  | 616,618-622 |
| **C** |  | GFCM-FAO |
| **Di** |  | DCF (EC), 306,315,317,377,534 |
| **55. Giant red shrimp:** *Aristaeomorpha foliacea* | | |
| **B** |  | International Bottom Trawl Survey in the Mediterranean (Medits) |
| **P/B** | log(P/B) = 10.154-0.271*LOG(M)-2824.247*(1/(T+273)-0.063*LOG(D+1)+0.13*(Me)+0.076*(Car)-0.311*(Mol)-0.154*(Crus)-0.266*(Pol)-0.472*(Echi) | 603,604 |
| **Q/B** |  | 583 |
| **D** |  | 614,622-628 |
| **C** |  | GFCM-FAO |
| **Di** |  | DCF (EC), 306,317,323,324,329,377,378,534 |
| **56. Other commercial shrimps:** *Penaeus kerathurus, Penaeus japonicus* | | |
| **B** |  | International Bottom Trawl Survey in the Mediterranean (Medits) |
| **P/B** | log(P/B) = 10.154-0.271*LOG(M)-2824.247*(1/(T+273)-0.063*LOG(D+1)+0.13*(Me)+0.076*(Car)-0.311*(Mol)-0.154*(Crus)-0.266*(Pol)-0.472*(Echi) | 603,604 |
| **Q/B** |  | 583 |
| **D** |  | 629 |
| **C** |  | GFCM-FAO |
| **Di** |  | DCF (EC), 313,377,390,534 |
| **57. No commercial shrimps:** *Acanthephyra eximia, Acanthephyra pelagica, Aegaeon cataphractus, Aegaeon lacazei, Allosergestes sargassi, Alpheus glaber, Alpheus macrocheles, Alpheus platydactylus, Ascidonia flavomaculata, Athanas nitescens, Calocarides coronatus, Calocaris macandreae, Caridion steveni, Chlorotocus crassicornis, Crangon, Eusergestes arcticus, Funchalia woodwardi, Gennadas elegans, Hymenopenaeus debilis, Ligur ensiferus, Lysmata seticaudata, Lophogaster typicus, Pandalina brevirostris, Pandalina profunda, Pasiphaea multidentata, Pasiphaea sivado, Penaeopsis serrata, Periclimenes granulatus, Philocheras echinulatus, Philocheras sculptus, Plesionika acanthonotus, Plesionika antigai, Plesionika edwardsii, Plesionika gigliolii, Plesionika heterocarpus, Plesionika martia, Plesionika narval, Pontophilus norvegicus, Pontophilus spinosus, Processa canaliculata, Processa edulis edulis, Processa elegantula, Processa nouveli, Richardina fredericii, Sergestes arachnipodus, Sergia robusta, Sicyonia carinata, Solenocera membranacea, Systellaspis debilis, Typton spongicola,* *Stenopus spinosus,* *Synalpheus gambarelloides, Upogebia pusilla* | | |
| **B** |  | International Bottom Trawl Survey in the Mediterranean (Medits) |
| **P/B** | log(P/B) = 10.154-0.271*LOG(M)-2824.247*(1/(T+273)-0.063*LOG(D+1)+0.13*(Me)+0.076*(Car)-0.311*(Mol)-0.154*(Crus)-0.266*(Pol)-0.472*(Echi) | 603,604 |
| **Q/B** |  | 583 |
| **D** |  | 614,630-639 |
| **C** |  | GFCM-FAO |
| **Di** |  | 313,324,329,378,389,534 |
| **58. Norway lobster:** *Nephrops norvegicus* | | |
| **B** |  | International Bottom Trawl Survey in the Mediterranean (Medits) |
| **P/B** | log(P/B) = 10.154-0.271*LOG(M)-2824.247*(1/(T+273)-0.063*LOG(D+1)+0.13*(Me)+0.076*(Car)-0.311*(Mol)-0.154*(Crus)-0.266*(Pol)-0.472*(Echi) | 603,604 |
| **Q/B** |  | 583 |
| **D** |  | 640-642 |
| **C** |  | GFCM-FAO |
| **Di** |  | DCF (EC), 313,317,324,329,377,378,389,390 |
| **59. Other commercial decapods:** *Homarus gammarus, Maja squinado, Necora puber, Palinurus elephas, Palinurus mauritanicus, Palinurus spp, Squilla mantis* | | |
| **B** |  | International Bottom Trawl Survey in the Mediterranean (Medits) |
| **P/B** | log(P/B) = 10.154-0.271*LOG(M)-2824.247*(1/(T+273)-0.063*LOG(D+1)+0.13*(Me)+0.076*(Car)-0.311*(Mol)-0.154*(Crus)-0.266*(Pol)-0.472*(Echi) | 603,604 |
| **Q/B** |  | 583 |
| **D** |  | 643-646 |
| **C** |  | GFCM-FAO |
| **Di** |  | DCF (EC), 247,306,313,326,329,339,401,534 |
| **60. Non-commercial decapods:** *Anamathia rissoana, Anapagurus bicorniger, Anapagurus laevis, Atelecyclus rotundatus, Bathynectes longipes, Bathynectes maravigna, Brachynotus sexdentatus, Calappa granulata, Calappa pelii, Calappa tuerkayana, Calcinus tubularis, Dardanus arrosor, Dardanus calidus, Derilambrus angulifrons, Dicranodromia mahieuxii, Dorhynchus thomsoni, Dromia personata, Ebalia cranchii, Ebalia deshayesi, Ebalia granulosa, Ebalia nux, Ebalia tuberosa, Ergasticus clouei, Eriphia verrucosa, Erugosquilla massavensis, Ethusa mascarone, Eurynome aspera, Galathea dispersa, Galathea intermedia, Galathea nexa, Galathea strigosa, Geryon longipes, Geryon trispinosus, Goneplax rhomboides, Homola barbata, Ilia nucleus, Inachus aguiarii, Inachus communissimus, Inachus dorsettensis, Inachus leptochirus, Inachus parvirostris, Inachus phalangium, Inachus thoracicus, Jaxea nocturna, Latreillia elegans, Liocarcinus corrugatus, Liocarcinus depurator, Liocarcinus maculatus, Liocarcinus navigator, Liocarcinus vernalis, Lissa chiragra, Macropipus tuberculatus, Macropodia linaresi, Macropodia longipes, Macropodia longirostris, Macropodia rostrata, Macropodia tenuirostris, Maja brachydactyla, Maja crispata, Maja goltziana, Medorippe lanata, Monodaeus couchii, Munida curvimana, Munida intermedia, Munida rugosa, Munida rutllanti, Munida tenuimana, Nepinnotheres pinnotheres, Paguristes eremita, Pagurus alatus, Pagurus anachoretus, Pagurus cuanensis, Pagurus excavates, Pagurus forbesii, Pagurus prideaux, Palicus caronii, Paractaea monodi, Parasquilla ferussaci, Paromola cuvieri, Parthenope expansa, Parthenopidae, Parthenopoides Massena, Pilumnus hirtellus, Pilumnus spinifer, Pilumnus villosissimus, Pinnotheres pisum, Pisa armata, Pisa nodipes, Pisidia longicornis, Polybius henslowii, Polycheles typhlops, Portumnus latipes, Portunus hastatus, Pseudosquillopsis cerisii, Rissoides desmaresti, Rissoides pallidus, Rochinia carpenter, Scyllarides latus, Scyllarus arctus, Scyllarus pygmaeus, Sphaerozius nitidus, Spinolambrus macrochelos, Thalamita poissonii, Thia scutellata, Xantho pilipes* | | |
| **B** |  | International Bottom Trawl Survey in the Mediterranean (Medits) |
| **P/B** | log(P/B) = 10.154-0.271*LOG(M)-2824.247*(1/(T+273)-0.063*LOG(D+1)+0.13*(Me)+0.076*(Car)-0.311*(Mol)-0.154*(Crus)-0.266*(Pol)-0.472*(Echi) | 603,604,647 |
| **Q/B** |  | 583 |
| **D** |  | 630,632,648-652 |
| **C** |  | GFCM-FAO |
| **Di** |  | 306,312,313,315,323-327,329,339,377,378,389,390,401,496,534 |
| **61. Mobile benthos:** *Amphipholis squamata, Amphipoda, Amphiura chiajei, Amphiura spp., Annelida, Anseropoda placenta, Antalis entalis, Antedon mediterranea, Antedon spp., Aphrodita aculeata, Aphrodita spp., Aspidosiphon muelleri muelleri, Asteroidea, Astropecten aranciacus, Astropecten bispinosus, Astropecten irregularis pentacanthus, Astropecten jonstoni, Astropecten spinulosus, Astropecten spp., Astrospartus mediterraneus, Bonelliidae, Brissopsis atlantica, Brissopsis atlantica mediterranea, Brissopsis lyrifera, Brissus unicolor, Centrostephanus longispinus, Ceramaster grenadensis, Chaetaster longipes, Chloeia venusta, Cidaris cidaris, Coscinasterias tenuispina, Dentaliidae, Diopatra neapolitana, Echinaster sepositus, Echinidae, Echinocardium cordatum, Echinocardium mediterraneum, Echinus melo, Euphrosine foliosa, Filograna implexa, Genocidaris maculata, Golfingia vulgaris vulgaris, Gracilechinus acutus, Hacelia attenuata, Havelockia inermis, Hediste diversicolor, Hirudinea, Holothuria forskali, Holothuria helleri, Holothuria mammata, Holothuria poli, Holothuria spp., Holothuria tubulosa, Hyalinoecia tubicola, Hymenodiscus coronata, Hyperiidae, Idotea spp., Isopoda, Labidoplax digitata, Laetmonice hystrix, Laetmonice spp., Leptometra phalangium, Leptometra spp., Leptopentacta elongate, Leptopentacta spp., Leptopentacta tergestina, Luidia ciliaris, Luidia sarsii, Luidia spp., Lumbrineris spp., Marthasterias glacialis, Mesothuria intestinalis, Molpadia musculus, Molpadia spp., Natatolana borealis, Ocnus planci, Ocnus syracusanus, Odontaster mediterraneus, Ophiacantha setosa, Ophidiaster ophidianus, Ophiocomina nigra, Ophioderma longicauda, Ophiomyxa pentagona, Ophiopsila aranea, Ophiothrix fragilis, Ophiothrix quinquemaculata, Ophiothrix spp., Ophiura albida, Ophiura ophiura, Ophiura spp., Ophiuridae, Ophiuroidea, Ova canaliferus, Oweniidae, Paracentrotus lividus, Parastichopus regalis, Peltaster placenta, Phronima sedentaria, Phronima spp., Phrosina semilunata, Phyllophorus urna, Plutonaster bifrons, Polychaeta, Pontobdella muricata, Priapulidae, Protula intestinum, Psammechinus microtuberculatus, Sipunculidae, Sipunculus nudus, Sipunculus spp., Spatangus purpureus, Spatangus subinermis, Sphaerechinus granularis, Sternaspis scutata, Stylocidaris affinis, Synaptidae, Tubulanus spp.* | | |
| **B** |  | International Bottom Trawl Survey in the Mediterranean (Medits) |
| **P/B** | log(P/B) = 10.154-0.271*LOG(M)-2824.247*(1/(T+273)-0.063*LOG(D+1)+0.13*(Me)+0.076*(Car)-0.311*(Mol)-0.154*(Crus)-0.266*(Pol)-0.472*(Echi) | 603,604 |
| **Q/B** |  | 583 |
| **D** |  | 653-657 |
| **Di** |  | 247,312,313,324,329,339,534 |
| **62. Sessile benthos:** *Actinauge richardi, Actinauge spp., Actinia spp., Actiniidae, Adamsia palliata, Adamsia spp., Agelas oroides, Amathia semiconvoluta, Andresia parthenopea, Aplidium conicum, Aplidium elegans, Aplidium haouarianum, Aplidium nordmanni, Aplidium pallidum, Aplidium proliferum, Aplidium spp., Ascidia involuta, Ascidia mentula, Ascidia spp., Ascidia virginea, Ascidiella aspersa, Ascidiella scabra, Ascidiella spp., Ascidiidae, Aulactinia verrucosa, Axinella cannabina, Axinella damicornis, Axinella polypoides, Axinella spp., Axinella verrucosa, Beania cylindrica, Botryllus schlosseri, Botryllus spp., Bryozoa, Bugula neritina, Bugula spp., Calliactis parasitica, Calpensia nobilis, Caryophyllia smithii, Caryophyllia spp., Cellaria salicornioides, Celleporina caliciformis, Cerianthus membranaceus, Cerianthus spp., Chondrosia reniformis, Ciona edwardsi, Ciona intestinalis, Ciona spp., Clathria coralloides, Cystodytes dellechiajei, Diazona violacea, Didemnidae, Didemnum maculosum, Didemnum spp., Diplosoma listerianum, Distaplia magnilarva, Distomus variolosus, Epizoanthus arenaceus, Epizoanthus paguricola, Epizoanthus spp., Eudistoma mucosum, Eudistoma spp., Filicrisia spp., Frondipora verrucosa, Funiculina quadrangularis, Geodia cydonium, Geodia spp., Halecium halecinum, Haliclona simulans, Halocynthia papillosa, Hippospongia communis, Hormathia alba, Hydractinia echinata, Hydrozoa, Ircinia spp., Lytocarpia myriophyllum, Metridium senile, Microcosmus claudicans, Microcosmus polymorphus, Microcosmus sabatieri, Microcosmus spp., Microcosmus squamiger, Microcosmus vulgaris, Molgula appendiculata, Molgula occulta, Molgula socialis, Molgula spp., Myriapora truncata, Nemertesia antennina, Nemertesia ramosa, Nemertesia spp., Parazoanthus axinellae, Pennatula aculeata, Pennatula phosphorea, Pennatula rubra, Pennatula spp., Pennatulidae, Pentapora fascialis fascialis, Pentapora foliacea, Petrosia ficiformis, Phallusia mammillata, Poecillastra compressa, Polycarpa mamillaris, Polycarpa pomaria, Polycarpa spp., Polycitor adriaticus, Polycitor crystallinus, Polycitor spp., Polyclinella azemai, Polyclinidae, Porifera, Protula tubularia, Pseudodistoma cyrnusense, Pteroeides griseum, Pteroeides spinosum, Pteroeides spp., Pyura dura, Pyura microcosmus, Pyura spp., Pyura tessellata, Reteporella beaniana, Reteporella grimaldii , Reteporella spp., Rhizaxinella pyrifera, Sabella spallanzanii, Sabella spp., Sabellidae, Sagartia elegans, Sarcotragus foetidus, Scalarispongia scalaris, Scalpellum scalpellum, Schizobrachiella sanguinea, Scrupocellaria scrupea, Serpula vermicularis, Serpulidae, Sertularella crassicaulis, Sertularella spp., Spongia officinalis, Spongiidae, Styela canopus, Styela spp., Suberites carnosus, Suberites domuncula, Suberites ficus, Suberites spp., Synoicum blochmanni, Telmatactis forskali, Tethya aurantium, Tethya citrina, Tethyaster subinermis, Thenea muricata, Veretillidae, Veretillum cynomorium, Veretillum spp., Virgularia mirabilis* | | |
| **B** |  | International Bottom Trawl Survey in the Mediterranean (Medits) |
| **P/B** | log(P/B) = 10.154-0.271*LOG(M)-2824.247*(1/(T+273)-0.063*LOG(D+1)+0.13*(Me)+0.076*(Car)-0.311*(Mol)-0.154*(Crus)-0.266*(Pol)-0.472*(Echi) | 603,604 |
| **Q/B** |  | 583 |
| **D** |  | 658-660 |
| **Di** |  | 312,324,329,339,534 |
| **63. Jellyfish&Salps:** *Aequorea forskalea, Aurelia aurita, Chrysaora hysoscella, Cnidaria , Pelagia noctiluca, Pleurobrachia pileus, Pyrosoma, Pyrosoma atlanticum, Rhizostoma pulmo, Thalia democratica, Salpa maxima, Salpa spp.* | | |
| **B** |  | 661 |
| **P/B** | M/K | 662 |
| **Q/B** | (P/B)/(P/Q) |  |
| **D** |  | 663-669 |
| **Di** |  | 313,324,339,389,670,671 |
| **64. Corals&gorgonians:** *Acanthogorgia hirsuta, Alcyonidium spp., Alcyonium acaule, Alcyonium palmatum, Alcyonium spp., Antipathella subpinnata, Antipathes dichotoma, Bebryce mollis, Callogorgia verticillata, Corallium rubrum, Desmophyllum dianthus, Eunicella cavolini, Eunicella filiformis, Eunicella singularis, Eunicella spp., Eunicella verrucosa, Funiculina quadrangularis, Gorgoniidae, Isidella elongata, Kophobelemnon stelliferum, Leptogorgia sarmentosa, Lophelia pertusa, Madrepora oculata, Paramuricea macrospina, Paramuricea clavata, Parantipathes larix, Pennatula spp., Swiftia pallida, Villogorgia bebrycoides, Viminella flagellum* | | |
| **B** |  | 672-688,689; International Bottom Trawl Survey in the Mediterranean (Medits) |
| **P/B** |  | 672,675,677,685,686,690,691 |
| **Q/B** | (P/B)/(P/Q) |  |
| **D** |  | 692-697 |
| **C** |  | GFCM-FAO |
| **Di** |  | 324,698 |
| **65. Zooplankton** | | |
| **B** |  | 699 |
| **P/B** |  | 699 |
| **Q/B** |  | 699 |
| **D** |  | 699 |
| **66. Seagrass:** *Cymodocea nodosa, Posidonia oceanica, Zoostera marina, Zoostera noltii* | | |
| **B** |  | 700-741 |
| **P/B** |  | 704,708,718,742-746 |
| **Di** |  | 312,725 |
|  | | |
| **67.** **Seaweed:** *Cystoseira barbata, Cystoseira brachycarpa balearica, Cystoseira cf. Elegans, Cystoseira compressa, Cystoseira compressa pustulata, Cystoseira corniculata, Cystoseira foeniculacea latiramosa, Cystoseira foeniculacea tenuiramosa, Cystoseira spinosa, Sargassum vulgare, Cutleria multifida, Hildenbrandia crouaniorum, Lobophora variegata, Palmophyllum crassum, Peyssonnelia rosa-marina, Polystrata fosliei, Pseudolithoderma adriaticum, Corallinaceae encrusting, Lithophyllum sp., Lithophyllum stictaeforme, Mesophyllum alternans, Acrodiscus vidovichii, Acrosymphyton purpuriferum, Amphiroa cryptarthrodia, Amphiroa rigida, Bonnemaisonia asparagoides, Caulerpa prolifera, Codium bursa, Codium cf. Coralloides, Codium effusum, Codium vermilara, Colpomenia sinuosa, Cryptonemia lomation, Dictyopteris polypodioides, Dictyota dichotoma, Dictyota fasciola, Dictyota implexa, Digenea simplex, Dudresnaya verticillata, Flabellia petiolata, Halimeda tuna, Halopteris filicina, Halopteris scoparia, Hydroclathrus clathratus, Liagora viscida, Nereia filiformis, Padina pavonica, Peyssonnelia harveyana, Peyssonnelia squamaria, Rytiphlaea tinctoria, Scinaia furcellata, Scinaia sp., Sebdenia sp., Sphaerococcus coronopifolius, Stilophora tenella, Taonia atomaria, Tricleocarpa fragilis, Ulva rigida, Zanardinia typus, Acrothamnion preissii, Asparagopsis armata, Asparagopsis taxiformis, Caulerpa racemosa cylindracea, Caulerpa racemosa lamourouxii, Lophocladia lallemandii, Stypopodium schimperi, Womersleyella setacea, Acetabularia acetabulum, Acinetospora crinita, Acrosorium ciliolatum, Alsidium corallinum, Anadyomene stellate, Asperococcus bullosus, Botryocladia botryoides, Botryocladia chiajeana, Bryopsis sp., Chaetomorpha sp., Champia parvula, Chondracanthus acicularis, Chondrophycus sp., Cladophora pellucida, Cladophora prolifera, Cladophora sp., Cladostephus spongiosus, Corallina elongate, Corallinaceae articulated, Cyanobacteria, Dasycladus vermicularis, Elachista intermedia, Gelidium bipectinatum, Gigartinales unidentified, Haliptilon virgatum, Irvinea boergesenii, Laurencia gr. Obtusa, Laurencia sp., Parvocaulis parvulus, Pseudochlorodesmis furcellata, Rhodymenia ardissonei, Sphacelaria cirrosa, Valonia utricularis, Wrangelia penicillata* | | |
| **B** |  | 747-757 |
| **P/B** |  | 753 |
| **68. Small phytoplankton:** *Dinoflagellates* | | |
| **B** |  | 699 |
| **P/B** |  | 699 |
|  | | |
| **B** |  | 699 |
| **P/B** |  | 699 |
|  | | |
| **B** |  | 699 |
| **Fisheries:** *Beach Seine, Dredge, Driftnet, Gillnet, Longline, Midwater Trawl, Purse Seine, Trammel net,* *Trap&Pot, Trawler* | | |
| **Catch/Di** |  | GFCM-FAO; DCF (EC) |
| **Effort** |  | GFCM-FAO; DCF (EC) |

**References**

1 Cañadas, A. & Hammond, P. Model-based abundance estimates for bottlenose dolphins off southern Spain: implications for conservation and management. *Journal of Cetacean Research and Management* **8**, 13 (2006).

2 Bompar, J., Baril, D., Dhermain, F. & Ripoll, T. Estimation of the Corsican population of bottlenose dolphins (Tursiops truncatus): is there a real conflict with fishermen. *European Research on Cetaceans* **8**, 92-94 (1994).

3 Fortuna, C. M. *Ecology and conservation of bottlenose dolphins (Tursiops truncatus) in the north-eastern Adriatic Sea*, University of St Andrews, (2006).

4 Dhermain, F. Suivi de la population du Grand Dauphin *Tursiops truncatus* en Corse. *Rapport GECEM pour le PNPC* (2006).

5 Forcada, J., Gazo, M., Aguilar, A., Gonzalvo, J. & Fernández-Contreras, M. Bottlenose dolphin abundance in the NW Mediterranean: addressing heterogeneity in distribution. *Marine Ecology Progress Series* **275**, 275-287 (2004).

6 Gannier, A. Summer distribution and relative abundance of delphinids in the Mediterranean Sea. *Rev Ecol (Terre Vie)* **60**, 223-238 (2005).

7 Gnone, G. *et al.* Distribution, abundance, and movements of the bottlenose dolphin (Tursiops truncatus) in the Pelagos Sanctuary MPA (north‐west Mediterranean Sea). *Aquatic Conservation: Marine and Freshwater Ecosystems* **21**, 372-388 (2011).

8 Gomez de Segura, A., Crespo, E., Pedraza, S., Hammond, P. & Raga, J. Abundance of small cetaceans in waters of the central Spanish Mediterranean. *Marine Biology* **150**, 149 (2006).

9 Gonzalvo, J., Forcada, J., Grau, E. & Aguilar, A. Strong site-fidelity increases vulnerability of common bottlenose dolphins Tursiops truncatus in a mass tourism destination in the western Mediterranean Sea. *Journal of the Marine Biological Association of the United Kingdom* **94**, 1227-1235 (2014).

10 Laran, S. *et al.* Seasonal distribution and abundance of cetaceans within French waters-Part I: The North-Western Mediterranean, including the Pelagos sanctuary. *Deep Sea Research Part II: Topical Studies in Oceanography* (2016).

11 Lauriano, G., Fortuna, C., Moltedo, G. & Notarbartolo di Sciara, G. Interactions between common bottlenose dolphins (Tursiops truncatus) and the artisanal fishery in Asinara Island National Park (Sardinia): assessment of catch damage and economic loss. *Journal of Cetacean Research and Management* **6**, 165-173 (2004).

12 Lauriano, G., Pierantonio, N., Donovan, G. & Panigada, S. Abundance and distribution of Tursiops truncatus in the Western Mediterranean Sea: An assessment towards the Marine Strategy Framework Directive requirements. *Marine environmental research* **100**, 86-93 (2014).

13 Lauriano, G., Mackelworth, P., Fortuna, C., Moltedo, G. & Notarbartolo di Sciara, G. Bottlenose dolphin(Tursiops truncatus) density and abundance in the Asinara National Park, Sardinia. *Biologia marina mediterranea* **10**, 717-720 (2003).

14 Manfredini, E. *et al.* Presence and abundance of bottlenose dolphin along the east Ligurian coast in relation to the pleasure boating. . (San Sebastián, Spain, 2007).

15 Nuti, S. *et al.* Stima di abbondanza di *Tursiops truncatus* tra La Spezia, Viareggio e Marina di Pisa attraverso cattura e ricattura fotografica. . (Cattolica (RI), Italy, 2006).

16 Ripoll, T. *et al.* First summer population estimate of bottlenose dolphins along the north-western coasts of the occidental Mediterranean basin. *European Research on Cetaceans* **15**, 393-396 (2004).

17 Pleslić, G. *et al.* The abundance of common bottlenose dolphins (Tursiops truncatus) in the former special marine reserve of the Cres‐Lošinj Archipelago, Croatia. *Aquatic Conservation: Marine and Freshwater Ecosystems* **25**, 125-137 (2015).

18 Fortuna, C., Holcer, D., Filidei, E., Donovan, G. & Tunesi, L. in *7th Meeting of the ACCOBAMS Scientific Committee.* 16.

19 Pulcini, M., Pace, D. S., La Manna, G., Triossi, F. & Fortuna, C. M. Distribution and abundance estimates of bottlenose dolphins (Tursiops truncatus) around Lampedusa Island (Sicily Channel, Italy): implications for their management. *Marine Biological Association of the United Kingdom. Journal of the Marine Biological Association of the United Kingdom* **94**, 1175 (2014).

20 ERA. MSFD Initial Assessement: marine mammals. ERA report, 33pp. (2019).

21 Bearzi, G. *et al.* Dolphins in a scaled-down Mediterranean: the Gulf of Corinth's odontocetes. *Advances in marine biology* **75**, 297-331 (2016).

22 Bearzi, G., Agazzi, S., Bonizzoni, S., Costa, M. & Azzellino, A. Dolphins in a bottle: abundance, residency patterns and conservation of bottlenose dolphins Tursiops truncatus in the semi‐closed eutrophic Amvrakikos Gulf, Greece. *Aquatic Conservation: Marine and Freshwater Ecosystems* **18**, 130-146 (2008).

23 Bearzi, G., FORTUNA, C. & Reeves, R. R. Ecology and conservation of common bottlenose dolphins Tursiops truncatus in the Mediterranean Sea. *Mammal Review* **39**, 92-123 (2009).

24 Bearzi, G., Holcer, D. & Notarbartolo di Sciara, G. The role of historical dolphin takes and habitat degradation in shaping the present status of northern Adriatic cetaceans. *Aquatic Conservation: Marine and Freshwater Ecosystems* **14**, 363-379 (2004).

25 Bearzi, G., Politi, E., Agazzi, S. & Azzellino, A. Prey depletion caused by overfishing and the decline of marine megafauna in eastern Ionian Sea coastal waters (central Mediterranean). *Biological Conservation* **127**, 373-382 (2006).

26 Naceur, L. B. *et al.* Recensement du grand dauphin Tursiops truncatus dans les eaux tunisiennes. *Bull. Inst. Natn. Scien. Tech. Mer de Salammbô* **31**, 76 (2004).

27 Santostasi, N. L., Bonizzoni, S., Bearzi, G., Eddy, L. & Gimenez, O. A robust design capture-recapture analysis of abundance, survival and temporary emigration of three Odontocete species in the Gulf of Corinth, Greece. *PloS one* **11**, e0166650 (2016).

28 Barlow, J. & Boveng, P. Modeling age‐specific mortality for marine mammal populations. *Marine Mammal Science* **7**, 50-65 (1991).

29 Pauly, D., Trites, A., Capuli, E. & Christensen, V. Diet composition and trophic levels of marine mammals. *ICES Journal of Marine Science: Journal du Conseil* **55**, 467-481 (1998).

30 Hunter, J. *A multiple regression model for predicting the energy requirements of marine mammals.* MSc degree thesis, University of British Columbia, (2005).

31 Pavan, G., Bernuzzi, E., Cozzi, B. & Podestà, M. The national network to monitor marine mammals strandings. *Biol. Mar. Mediterr.* **20** 262-226 (2013).

32 Kastelein, R., Staal, C. & Wiepkema, P. Food consumption, food passage time, and body measurements of captive Atlantic bottlenose dolphins (Tursiops truncatus). *Aquatic Mammals* **29**, 53-66 (2003).

33 Blanco, C., Salomón, O. & Raga, J. Diet of the bottlenose dolphin (Tursiops truncatus) in the western Mediterranean Sea. *Journal of the Marine Biological Association of the UK* **81**, 1053-1058 (2001).

34 Aznar, F., Fognani, P., Balbuena, J. A., Pietrobelli, M. & Raga, J. Distribution of Pholeter gastrophilus (Digenea) within the stomach of four odontocete species: the role of the diet and digestive physiology of hosts. *Parasitology* **133**, 369 (2006).

35 Orsi Relini, L., Cappello, M. & Poggi, R. The stomach content of some bottlenose dolphins (Tursiops truncatus) from the Ligurian Sea. *European Research on Cetaceans* **8**, 192-195 (1994).

36 Montenegro Benalcázar, V. E. *Modelo de nicho ecológico para el delfín mular (Tursiops Truncatus) en el mar Balear* Máster Universitario en Evaluación y Seguimiento Ambiental de Ecosistemas Marinos y Costeros thesis, Universitat Politècnica de València. Departamento de Ciencia Animal - Departament de Ciència Animal (2018).

37 Milani, C. *et al.* Cetacean stranding and diet analyses in the North Aegean Sea (Greece). *Marine Biological Association of the United Kingdom. Journal of the Marine Biological Association of the United Kingdom* **98**, 1011-1028 (2018).

38 Ancha, L. Regional by-catch of long-lived species (sea birds, marine mammals and sea turtles) in the Mediterranean and Black Seas. *Masters project to be submitted in partial fulfillment of the requirements for the Master of Environmental Management degree in the Nicholas School of the Environment and Earth Sciences of Duke University* (2008).

39 Di Natale, A. By-catch of Marine Mammals in Tuna and Swordfish Fisheries: the Mediterranean Case. *ICES CM* (1997).

40 Reeves, R. R., McClellan, K. & Werner, T. B. Marine mammal bycatch in gillnet and other entangling net fisheries, 1990 to 2011. *Endangered Species Research* **20**, 71-97 (2013).

41 Lewison, R. L. *et al.* Global patterns of marine mammal, seabird, and sea turtle bycatch reveal taxa-specific and cumulative megafauna hotspots. *Proceedings of the National Academy of Sciences* **111**, 5271-5276 (2014).

42 European Commission. Report o the second meeting of the subgroup on fishery and environment (SGFEN) of the Scientific, Technical and Economic Committee for Fisheries (STECF). Incidental catches of small cetaceans. (Brussels, 2002).

43 Crosti, R., Arcangeli, A., Romeo, T. & Andaloro, F. Assessing the relationship between cetacean strandings (Tursiops truncatus and Stenella coeruleoalba) and fishery pressure indicators in Sicily (Mediterranean Sea) within the framework of the EU Habitats Directive. *European journal of wildlife research* **63**, 55 (2017).

44 Díaz-López, B. Interactions between bottlenose dolphins with trammel nets in the Sardinia Island. *ICES Document CM* **10**, 01 (2005).

45 Fortuna, C. M. *et al.* By-catch of cetaceans and other species of conservation concern during pair trawl fishing operations in the Adriatic Sea (Italy). *Chemistry and Ecology* **26**, 65-76 (2010).

46 Fortuna, C. M., Vallini, C., De Carlo, F., Filidei, E.jr, Lucchetti, A., Gaspari, S., Fossi, M.C., Maltese, S., & Marsili, L., Bottaro, M., Ruffino, M., Scacco, U., Giovanardi, O., Mazzola, A., Sala, A., Tunesi, L. Relazione finale del progetto “Valutazione delle catture accidentali di specie protette nel traino pelagico (BYCATCH III)”, codice progetto: 7A02. 84 pagine + Allegati., (2010).

47 Gannier, A. Summer cetacean popoulation in the Pelagos Marine Sanctuary (northwest Mediterranean): distribution and abundance. *Mammalia* **70**, 17-27 (2006).

48 Cotté, C., Guinet, C., Taupier-Letage, I. & Petiau, E. Habitat use and abundance of striped dolphins in the western Mediterranean Sea prior to the morbillivirus epizootic resurgence. *Endangered Species Research* **12**, 203-214 (2010).

49 Forcada, J., Notarbartolo di Sciara, G. & Fabbri, F. Abundance of fin whales and striped dolphins summering in the Corso-Ligurian Basin. *Mammalia* **59**, 127-140 (1995).

50 Laran, S., Joiris, C., Gannier, A. & Kenney, R. D. Seasonal estimates of densities and predation rates of cetaceans in the Ligurian Sea, northwestern Mediterranean Sea: an initial examination. *J Cetacean Res Manag* **11**, 31-40 (2010).

51 Bauer, R. K., Fromentin, J.-M., Demarcq, H., Brisset, B. & Bonhommeau, S. Co-Occurrence and Habitat Use of Fin Whales, Striped Dolphins and Atlantic Bluefin Tuna in the Northwestern Mediterranean Sea. *PloS one* **10**, e0139218 (2015).

52 Forcada, J. & Hammond, P. Geographical variation in abundance of striped and common dolphins of the western Mediterranean. *Journal of sea research* **39**, 313-325 (1998).

53 Forcada, J., Aguilar, A., Hammond, P. S., Pastor, X. & Aguilar, R. Distribution and numbers of striped dolphins in the western Mediterranean Sea after the 1990 epizootic outbreak. *Marine Mammal Science* **10**, 137-150 (1994).

54 Fortuna, C. M. *et al.* An insight into the status of the striped dolphins, Stenella coeruleoalba, of the southern Tyrrhenian Sea. *Journal of the Marine Biological Association of the United Kingdom* **87**, 1321-1326 (2007).

55 Lauriano, G., Panigada, S., Canneri, R. & M.M, Z. Abundance estimate of striped dolphins (Stenella coeruleoalba) in the Pelagos Sanctuary (NW Mediterranean) by means of line transect surveys. *J Cetacean Res Manag SC-61-SM25* (2009).

56 Panigada, S., Lauriano, G., Burt, L., Pierantonio, N. & Donovan, G. Monitoring winter and summer abundance of cetaceans in the Pelagos Sanctuary (northwestern Mediterranean Sea) through aerial surveys. *PloS one* **6**, e22878 (2011).

57 Aguilar, A. Population biology, conservation threats and status of Mediterranean striped dolphins(Stenella coeruleoalba). *J. Cetacean Res. Manag.* **2**, 17-26 (2000).

58 Marsili, L., Casini, C., Marini, L., Regoli, A. & Focardi, S. Age, growth and organochlorines (HCB, DDTs and PCBs) in Mediterranean striped dolphins Stenella coeruleoalba stranded in 1988-1994 on the coasts of Italy. *Marine Ecology Progress Series* **151**, 273-282 (1997).

59 Di-Méglio, N., Romero-Alvarez, R. & Collet, A. Growth comparison in striped dolphins, Stenella coeruleoalba, from the Atlantic and Mediterranean coasts of France. *Aquatic Mammals* **22**, 11-21 (1996).

60 Aguilar, A. & Raga, J. A. The striped dolphin epizootic in the Mediterranean Sea. *Ambio*, 524-528 (1993).

61 Würtz, M. & Marrale, D. Food of striped dolphin, Stenella coeruleoalba, in the Ligurian Sea. *Journal of the Marine Biological Association of the United Kingdom* **73**, 571-578 (1993).

62 Blanco, C., Aznar, J. & Raga, J. Cephalopods in the diet of the striped dolphin Stenella coeruleoalba from the western Mediterranean during an epizootic in 1990. *Journal of Zoology* **237**, 151-158 (1995).

63 Gómez-Campos, E., Borrell, A., Cardona, L., Forcada, J. & Aguilar, A. Overfishing of small pelagic fishes increases trophic overlap between immature and mature striped dolphins in the Mediterranean Sea. *PloS one* **6**, e24554 (2011).

64 Aznar, F. J. *et al.* Long-term changes (1990-2012) in the diet of striped dolphins Stenella coeruleoalba from the western Mediterranean. *Marine Ecology Progress Series* **568**, 231-247 (2017).

65 Berti, A. *Analisi del contenuto stomacale degli esemplari di stenella striata (S. coeruleoalba, Meyen, 1833) spiaggiati lungo le coste della Toscana* Master Degree thesis, Universita di Pisa, (2013).

66 Dede, A., Salman, A. & Tonay, A. M. Stomach contents of by-caught striped dolphins (Stenella coeruleoalba) in the eastern Mediterranean Sea. *Marine Biological Association of the United Kingdom. Journal of the Marine Biological Association of the United Kingdom* **96**, 869 (2016).

67 Banaru, D., DEKEYSER, I., IMBERT, G. & LAUBIER, L. Non-target and released alive by-catches distributions observed during French driftnet fishery in the Northwestern Mediterranean Sea (2000-2003 database). *Journal of Oceanography, Research and Data* **3** (2010).

68 López, D. M., Barcelona, S. G., Báez, J. C., De la Serna, J. M. & de Urbina, J. M. O. Marine mammal bycatch in Spanish Mediterranean large pelagic longline fisheries, with a focus on Risso’s dolphin (Grampus griseus). *Aquatic Living Resources* **25**, 321-331 (2012).

69 Silvani, L., Gazo, M. & Aguilar, A. Spanish driftnet fishing and incidental catches in the western Mediterranean. *Biological Conservation* **90**, 79-85 (1999).

70 Garibaldi, F. By-catch in the mesopelagic swordfish longline fishery in the Ligurian Sea (Western Mediterranean). *Collect. Vol. Sci. Pap. ICCAT* **71**, 1495-1498 (2015).

71 Cañadas, A. & Hammond, P. Abundance and habitat preferences of the short-beaked common dolphin Delphinus delphis in the southwestern Mediterranean: implications for conservation. *Endangered Species Research* **4**, 309 (2008).

72 Pace, D., Mussi, B, Vella, A, Vella, J, Frey, S, Bearzi, G, Benamer, I, Benmessaoud, R, Gannier, A,, Genov, T., Giménez, J, Gonzalvo, J, Kerem, D, Larbi Doukara, K, Milani, C, Murphy, S, Natoli, A, & Öztürk, A., Pierce, GJ Report of the 1st International Workshop “Conservation and Research Networking on Short‐beaked Common Dolphin (*Delphinus delphis*) in the Mediterranean Sea” (Ischia Island, Italy, 2016).

73 Kastelein, R., Macdonald, G. & Wiepkema, P. A note on food consumption and growth of common dolphins (Delphinus delphis). *Journal of Cetacean Research and Management* **2**, 69-74 (2000).

74 Orsi Relini, L. & Relini, M. The stomach content of some common dolphins (Delphinus delphis L.) from the Ligurian Sea. *European research on cetaceans* **7**, 99-102 (1993).

75 Giménez, J. *et al.* Feeding ecology of Mediterranean common dolphins: The importance of mesopelagic fish in the diet of an endangered subpopulation. *Marine Mammal Science* **34**, 136-154 (2018).

76 Gannier, A. Estimation de l'abondance estivale du Rorqual commun Balaenoptera physalus (Linné, 1758) dans le bassin Liguro-provençal (Méditerranée occidentale). (1997).

77 Forcada, J., Aguilar, A., Hammond, P., Pastor, X. & Aguilar, R. Distribution and abundance of fin whales (Balaenoptera physalus) in the western Mediterranean sea during the summer. *Journal of Zoology* **238**, 23-34 (1996).

78 Monestiez, P., Dubroca, L., Bonnin, E., Durbec, J.-P. & Guinet, C. Geostatistical modelling of spatial distribution of Balaenoptera physalus in the Northwestern Mediterranean Sea from sparse count data and heterogeneous observation efforts. *Ecological Modelling* **193**, 615-628 (2006).

79 Notarbartolo di Sciara, G., Castellote, M., Druon, J.-N. & Panigada, S. Fin whales, Balaenoptera physalus: At home in a changing Mediterranean Sea? *Advances in marine biology* **75**, 75-101 (2016).

80 Notarbartolo di Sciara, G., Zanardelli, M., Jahoda, M., Panigada, S. & Airoldi, S. The fin whale Balaenoptera physalus (L. 1758) in the Mediterranean Sea. *Mammal Review* **33**, 105-150 (2003).

81 Notarbartolo di Sciara, G. *et al.* Cetaceans in the central Mediterranean Sea: distribution and sighting frequencies. *Italian Journal of Zoology* **60**, 131-138 (1993).

82 Laran, S. & Gannier, A. Spatial and temporal prediction of fin whale distribution in the northwestern Mediterranean Sea. *ICES Journal of Marine Science* **65**, 1260-1269 (2008).

83 Canese, S. *et al.* The first identified winter feeding ground of fin whales (Balaenoptera physalus) in the Mediterranean Sea. *Journal of the Marine Biological Association of the United Kingdom* **86**, 903-907 (2006).

84 Cotté, C., Guinet, C., Taupier-Letage, I., Mate, B. & Petiau, E. Scale-dependent habitat use by a large free-ranging predator, the Mediterranean fin whale. *Deep Sea Research Part I: Oceanographic Research Papers* **56**, 801-811 (2009).

85 Bentaleb, I. *et al.* Foraging ecology of Mediterranean fin whales in a changing environment elucidated by satellite tracking and baleen plate stable isotopes. *Marine Ecology Progress Series* **438**, 285-302 (2011).

86 Giménez, J., Gómez‐Campos, E., Borrell, A., Cardona, L. & Aguilar, A. Isotopic evidence of limited exchange between Mediterranean and eastern North Atlantic fin whales. *Rapid Communications in Mass Spectrometry* **27**, 1801-1806 (2013).

87 Pace, D. S., Miragliuolo, A., Mariani, M., Vivaldi, C. & Mussi, B. Sociality of sperm whale off Ischia Island (Tyrrhenian Sea, Italy). *Aquatic Conservation: Marine and Freshwater Ecosystems* **24**, 71-82 (2014).

88 Rendell, L. *et al.* Abundance and movements of sperm whales in the western Mediterranean basin. *Aquatic Conservation: Marine and Freshwater Ecosystems* **24**, 31-40 (2014).

89 Gannier, A., Drouot, V. & Goold, J. C. Distribution and relative abundance of sperm whales in the Mediterranean Sea. *Marine Ecology Progress Series* **243**, 281-293 (2002).

90 Frantzis, A., Airoldi, S., Notarbartolo-di-Sciara, G., Johnson, C. & Mazzariol, S. Inter-basin movements of Mediterranean sperm whales provide insight into their population structure and conservation. *Deep Sea Research Part I: Oceanographic Research Papers* **58**, 454-459 (2011).

91 Frantzis, A., Alexiadou, P. & Gkikopoulou, K. C. Sperm whale occurrence, site fidelity and population structure along the Hellenic Trench (Greece, Mediterranean Sea). *Aquatic Conservation: Marine and Freshwater Ecosystems* **24**, 83-102 (2014).

92 Frantzis, A. *et al.* Current knowledge of the cetacean fauna of the Greek Seas. *Journal of Cetacean Research and Management* **5**, 219-232 (2003).

93 Rendell, L. & Frantzis, A. Mediterranean sperm whales, Physeter macrocephalus: the precarious state of a lost tribe. *Advances in marine biology* **75**, 37-74 (2016).

94 Lewis, T. *et al.* Abundance estimates for sperm whales in the Mediterranean Sea from acoustic line-transect surveys. (2018).

95 Drouot, V., Gannier, A. & Goold, J. C. Diving and feeding behaviour of sperm whales (Physeter macrocephalus) in the northwestern Mediterranean Sea. *Aquatic mammals* **30**, 419-426 (2004).

96 Praca, E. & Gannier, A. Ecological niches of three teuthophageous odontocetes in the northwestern Mediterranean Sea. *Ocean Science* **4** (2008).

97 Roberts, S. M. Examination of the stomach contents from a Mediterranean sperm whale found south of Crete, Greece. *Journal of the Marine Biological Association of the UK* **83**, 667-670 (2003).

98 Notarbartolo di Sciara, G., Bearzi, G., Canadas, A. & Frantzis, A. High mortality of sperm whales in the north-western Mediterranean, 1971-2003. *Paper submitted to the Scientific Committee of the International Whaling Commission (SC/56/BC10). Sorrento, Italy* (2004).

99 Rosso, M. *Modello bioenergetico per la stima del fabbisogno trofico dei cetacei odontoceti nel Mediterraneo nord-occidentale* PhD thesis, Università degli studi della Basilicata, (2009).

100 Rosso, M., Ballardini, M., Moulins, A. & Würtz, M. Natural markings of Cuvier's beaked whale Ziphius cavirostris in the Mediterranean Sea. *African Journal of Marine Science* **33**, 45-57 (2011).

101 Podestà, M. *et al.* Cuvier's Beaked Whale, Ziphius cavirostris, Distribution and Occurrence in the Mediterranean Sea: High-Use Areas and Conservation Threats. *Advances in Marine Biology* **75**, 103-140 (2016).

102 Gannier, A. Using existing data and focused surveys to highlight Cuvier’s beaked whales favourable areas: A case study in the central Tyrrhenian Sea. *Marine pollution bulletin* **63**, 10-17 (2011).

103 Gannier, A. & Epinat, J. Cuvier's beaked whale distribution in the Mediterranean Sea: results from small boat surveys 1996-2007. *Marine Biological Association of the United Kingdom. Journal of the Marine Biological Association of the United Kingdom* **88**, 1245 (2008).

104 Cañadas, A. & Vázquez, J. Conserving Cuvier’s beaked whales in the Alboran Sea (SW Mediterranean): Identification of high density areas to be avoided by intense man-made sound. *Biological Conservation* **178**, 155-162 (2014).

105 Verborgh, P. *et al.* Conservation Status of Long-Finned Pilot Whales, Globicephala melas, in the Mediterranean Sea. *Advances in Marine Biology* **75**, 173-203 (2016).

106 Azzellino, A. *et al.* Risso's Dolphin, Grampus griseus, in the Western Ligurian Sea: Trends in Population Size and Habitat Use. *Advances in Marine Biology* **75**, 205-232 (2016).

107 Cañadas, A. *et al.* The challenge of habitat modelling for threatened low density species using heterogeneous data: the case of Cuvier’s beaked whales in the Mediterranean. *Ecological Indicators* **85**, 128-136 (2018).

108 Blanco, C., Raduán, M. Á. & Raga, J. A. Diet of Risso's dolphin (Grampus griseus) in the western Mediterranean Sea. *Scientia Marina* **70**, 407-411 (2006).

109 Würtz, M., Poggi, R. & Clarke, M. R. Cephalopods from the stomachs of a Risso's dolphin (Grampus griseus) from the Mediterranean. *Journal of the Marine Biological Association of the United Kingdom* **72**, 861-867 (1992).

110 Cañadas, A. & Sagarminaga, R. The Northeastern Alboran Sea, an important breeding and feeding ground for the long-finned pilot whale (Globicephala melas) in the Mediterranean Sea. *Marine Mammal Science* **16**, 513-529 (2000).

111 De Stephanis, R. *et al.* Diet of the social groups of long-finned pilot whales (Globicephala melas) in the Strait of Gibraltar. *Marine Biology* **154**, 603-612 (2008).

112 Santos, M. B. *et al.* Feeding ecology of Cuvier's beaked whale (Ziphius cavirostris): a review with new information on the diet of this species. *Journal of the Marine Biological Association of the UK* **81**, 687-694 (2001).

113 Mo, G. Mediterranean monk seal (Monachus monachus) sightings in Italy (1998-2010) and implications for conservation. *Aquatic Mammals* **37**, 236 (2011).

114 Mo, G., Agnesi, S., Di Nora, T. & Tunesi, L. Mediterranean monk seal sightings in Italy through interviews: Validating the information (1998–2006). *Rapport Commission International Mer Méditerranée* **38**, 542 (2007).

115 Mo, G., Bazairi, H., Bayed, A. & Agnesi, S. Survey on Mediterranean monk seal (Monachus monachus) sightings in Mediterranean Morocco. *Aquatic Mammals* **37**, 248 (2011).

116 Bouderbala, M. *et al.* Résultats de la campagne de sensibilisation et recherche de la possible présence de phoque moine sur le littoral occidental d'Algérie. (2006).

117 Aguilar, A. Current status of Mediterranean monk seal (Monachus monachus) populations. *IUCN, Gland, Switzerland* (1998).

118 UNEP/MAP (United Nations Environment Programme/Mediterranean Action Plan). Report of the Meeting of Expert on the Evaluation of the Implementation of the Action Plan for the Management of the Mediterranean Monk Seal. UNEP(OCA)/MED WG.87/4. (Tunis, 1994).

119 Notarbartolo di Sciara, G. & Kotomatas, S. Are Mediterranean Monk Seals, Monachus monachus, Being Left to Save Themselves from Extinction? *Advances in Marine Biology* **75**, 359-386 (2016).

120 UNEP/MAP (United Nations Environment Programme/Mediterranean Action Plan). Current status of Mediterranean monk seal (*Monachus monachus*) populations. UNEP(OCA)/MED WG.146/4. (Tunis, 1998).

121 Gomerčić, T., Huber, D., Gomerčić, M. & Gomerčić, H. Presence of the Mediterranean monk seal (Monachus monachus) in the Croatian part of the Adriatic Sea. *Aquatic Mammals* **37**, 243-247 (2011).

122 Hamza, A., Mo, G. & Tayeb, K. Results of a preliminary mission carried out in Cyrenaica, Libya, to assess monk seal presence and potential coastal habitat. *The Monachus Guardian* **6** (2003).

123 Karamanlidis, A. A. *et al.* The Mediterranean monk seal *Monachus monachus*: status, biology, threats, and conservation priorities. *Mammal Review* **46**, 92-105 (2016).

124 Murphy, S. *et al.* Age estimation, growth and age-related mortality of Mediterranean monk seals Monachus monachus. *Endangered Species Research* **16**, 149-163 (2012).

125 Margaritoulis, D. & Touliatou, S. Mediterranean monk seals present an ongoing threat for loggerhead sea turtles in Zakynthos. *Marine Turtle Newsletter*, 18 (2011).

126 Karamanlidis, A. A., Kallianiotis, A., Psaradellis, M. & Adamantopoulou, S. Stomach contents of a subadult Mediterranean monk seal (Monachus monachus) from the Aegean Sea. *Aquatic Mammals* **37**, 280 (2011).

127 Pierce, G. J. *et al.* Diet of the monk seal (Monachus monachus) in Greek waters. *Aquatic Mammals* **37**, 284 (2011).

128 Salman, A., Bilecenoglu, M. & Güçlüsoy, H. Stomach contents of two Mediterranean monk seals (Monachus monachus) from the Aegean Sea, Turkey. *Journal of the Marine Biological Association of the UK* **81**, 719-720 (2001).

129 Karamanlidis, A. A. *et al.* Assessing accidental entanglement as a threat to the Mediterranean monk seal Monachus monachus. *Endangered Species Research* **5**, 205-213 (2008).

130 MEDMARAVIS & Monbailliu, X. *Mediterranean Marine Avifauna.*, Vol. 12 (Springer, 1986).

131 Garcia Robles, H., Deceuninck, B. & Micol, T. Status Report for Yelkouan Shearwater Puffinus Yelkouan (2nd draft). *Project LIFE 14 PRE/UK/000002 Coordinated Efforts for International Species Recovery EuroSAP. Ligue pour la Protection des Oiseaux, BirdLife* 22 pp (2016).

132 Baccetti, N. *et al.* Breeding shearwaters on Italian islands: population size, island selection and co-existence with their main alien predator, the black rat. *Riv. Ital. Orn* **78**, 83-100 (2009).

133 Arcos, J. M. *et al.* in *Proceedings of the 13th Medmaravis Pan-Mediterranean Symposium.* 84-94.

134 Abelló, P. & Oro, D. Offshore distribution of seabirds in the northwestern Mediterranean in June 1995. *Colonial Waterbirds*, 422-426 (1998).

135 Amengual, J. *et al.* The Mediterranean Storm Petrel Hydrobates pelagicus melitensis at Cabrera archipelago (Balearic Islands, Spain): Breeding moult, biometry and evaluation of the population size by mark and recapture techniques. *Ringing & Migration* **19**, 181-190 (1999).

136 Arcos, J. M. *et al.* Assessing the location and stability of foraging hotspots for pelagic seabirds: an approach to identify marine Important Bird Areas (IBAs) in Spain. *Biological conservation* **156**, 30-42 (2012).

137 Arroyo, G. M. *et al.* New population estimates of a critically endangered species, the Balearic Shearwater Puffinus mauretanicus, based on coastal migration counts. *Bird Conservation International* **26**, 87-99 (2016).

138 Bourgeois, K. & Vidal, E. The endemic Mediterranean yelkouan shearwater Puffinus yelkouan: distribution, threats and a plea for more data. *Oryx* **42**, 187-194 (2008).

139 Carboneras, C., Derhé, M. & Ramírez, I. in *Report to Seventh Meeting of the ACAP Advisory Committee.* 6-10.

140 Defos du Rau, P. *et al.* Reassessment of the size of the Scopoli’s Shearwater population at its main breeding site resulted in a tenfold increase: implications for the species conservation. *Journal of Ornithology* **156**, 877-892 (2015).

141 Gallo-Orsi, U. Species Action Plans for the conservation of seabirds in the Mediterranean Sea: Audouin's gull, Balearic shearwater and Mediterranean shag. *Scientia Marina* **67**, 47-55 (2003).

142 García-Barcelona, S. *et al.* Modelling abundance and distribution of seabird by-catch in the Spanish Mediterranean longline fishery. (2010).

143 Genovart, M. *et al.* Demography of the critically endangered Balearic shearwater: the impact of fisheries and time to extinction. *Journal of Applied Ecology* **53**, 1158-1168 (2016).

144 Grémillet, D. *et al.* Irreplaceable area extends marine conservation hotspot off Tunisia: insights from GPS-tracking Scopoli’s shearwaters from the largest seabird colony in the Mediterranean. *Marine biology* **161**, 2669-2680 (2014).

145 Yésou, P., Bacceti, N. & Sultana, J. in *Proceedings of the 13th MEDMARAVIS Pan-Mediterranean Symposium, Alghero (Sardinia).* 84-94.

146 Oro, D. & Ruiz, X. Exploitation of trawler discards by breeding seabirds in the north-western Mediterranean: differences between the Ebro Delta and the Balearic Islands areas. *ICES Journal of Marine Science: Journal du Conseil* **54**, 695-707 (1997).

147 Paracuellos, M. & Nevado, J. C. Nesting seabirds in SE Spain: distribution, numbers and trends in the province of Almería. *Scientia Marina* **67**, 125-128 (2003).

148 Paracuellos, M. & Jerez, D. A comparison of two seabird communities on opposite coasts of the Alborán Sea (western Mediterranean). *Scientia Marina* **67**, 117-123 (2003).

149 Boué, A. *et al.* in *First Meeting of the Population and Conservation Status Working Group*

150 Péron, C. *et al.* Importance of coastal Marine Protected Areas for the conservation of pelagic seabirds: The case of Vulnerable yelkouan shearwaters in the Mediterranean Sea. *Biological conservation* **168**, 210-221 (2013).

151 Pettex, E. *et al.* Using large scale surveys to investigate seasonal variations in seabird distribution and abundance. Part I: The North Western Mediterranean Sea. *Deep Sea Research Part II: Topical Studies in Oceanography* (2016).

152 Thibault, J.-C., Zotier, R., Guyot, I. & Bretagnolle, V. Recent trends in breeding marine birds of the Mediterranean region with special reference to Corsica. *Colonial Waterbirds*, 31-40 (1996).

153 ICES. Report of the Working Group on Seabird Ecology (WGSE). *ICES CM 2008/LRC:05*, 99 pp. (2008).

154 Zotier, R., Bretagnolle, V. & Thibault, J. C. Biogeography of the marine birds of a confined sea, the Mediterranean. *Journal of Biogeography* **26**, 297-313 (1999).

155 MEDMARAVIS. in *Proceedings of the 13th Medmaravis Pan-Mediterranean Symposium.* (eds P Yesou, N Baccetti, & J Sultana).

156 Peronace, V., Cecere, J. G., Gustin, M. & Rondinini, C. Lista Rossa 2011 degli uccelli nidificanti in Italia. *Avocetta* **36**, 11-58 (2012).

157 Mante, A. & Debize, E. Mediterranean storm petrel *Hydrobates pelagicus melitensis*, Updated state of knowledge & conservation of the nesting populations of the Mediterranean Small Island. Initiative PIM. 20p. (2012).

158 BirdLife International. European Red List of Birds. Luxembourg: Office for Official Publications of the European Communities., (Wageningen, 2015).

159 UNEP-MAP-RAC/SPA. Sicily Channel/Tunisian Plateau: Status and conservation of Seabirds. 22 pp (Tunis, 2015).

160 Nardelli, R. *et al.* Rapporto sull’applicazione della Direttiva 147/2009/CE in Italia: dimensione, distribuzione e trend delle popolazioni di uccelli (2008–2013). *ISPRA, Serie Rapporti* **219**, 2015 (2015).

161 Zakkak, S., Panagiotopoulou, M. & Halley, J. M. Estimating abundance patterns of seabirds in the north Aegean Sea. *Marine Ornithology* **41**, 141-148 (2013).

162 Krebs, C. J. *Ecological methodology*. (Harper & Row New York, 1989).

163 Karpouzi, V. S., Watson, R. & Pauly, D. Modelling and mapping resource overlap between seabirds and fisheries on a global scale: a preliminary assessment. *Marine Ecology Progress Series* **343**, 87-99 (2007).

164 Paleczny, M. *An analysis of temporal and spatial patterns in global seabird abundance during the modern industrial era, 1950-2010, and the relationship between global seabird decline and marine fisheries catch* Master degree thesis, University of British Columbia, (2012).

165 Arcos, J. *Balearic shearwater and fisheries. Foraging ecolgy of seabirds at sea: significance of commercial fisheries in the Northwest Mediterranean* PhD thesis thesis, Universitat de Barcelona, (2001).

166 Bourgeois, K., Vorenger, J., Faulquier, L., Legrand, J. & Vidal, E. Diet and contamination of the Yelkouan Shearwater Puffinus yelkouan in the Hyères archipelago, Mediterranean Basin, France. *Journal of ornithology* **152**, 947-953 (2011).

167 Albores‐Barajas, Y. *et al.* Diet and diving behaviour of European Storm Petrels Hydrobates pelagicus in the Mediterranean (ssp. melitensis). *Bird Study* **58**, 208-212 (2011).

168 MEDMARAVIS. in *Proceedings of the 2nd Medmaravis Pan-Mediterranean Symposium.* (eds JS Aguilar, X Monbailliu, & AM Paterson).

169 Báez, J. C. *et al.* Cory’s shearwater by-catch in the Mediterranean Spanish commercial longline fishery: implications for management. *Biodiversity and conservation* **23**, 661-681 (2014).

170 Genovart, M. *et al.* Varying demographic impacts of different fisheries on three Mediterranean seabird species. *Global change biology* **23**, 3012-3029 (2017).

171 Doxa, A. *et al.* Inferring dispersal dynamics from local population demographic modelling: the case of the slender‐billed gull in France. *Animal Conservation* **16**, 684-693 (2013).

172 Aguilar, J. & Fernández, G. Species action plan for the Mediterranean Shag Phalacrocorax aristotelis desmarestii in Europe. *BirdLife International, Cambridge, UK* (1999).

173 Bosch, M., Oro, D., Cantos, F. J. & Zabala, M. Short‐term effects of culling on the ecology and population dynamics of the yellow‐legged gull. *Journal of Applied Ecology* **37**, 369-385 (2000).

174 Bregnballe, T. *et al.* Breeding numbers of great cormorants Phalacrocorax carbo in the western Palearctic, 2012-2013. *IUCN-Wetlands International Cormorant Research Group Report. - Scientific Report from DCE – Danish Centre for Environment and Energy* **No. 99**, 224 pp (2014).

175 Cama, A., Josa, P., Ferrer-Obiol, J. & Arcos, J. M. Mediterranean Gulls Larus melanocephalus wintering along the Mediterranean Iberian coast: numbers and activity rhythms in the species’ main winter quarters. *Journal of Ornithology* **152**, 897-907 (2011).

176 Cama, A., Abellana, R., Christel, I., Ferrer, X. & Vieites, D. R. Living on predictability: modelling the density distribution of efficient foraging seabirds. *Ecography* **35**, 912-921 (2012).

177 Carboneras, C. *Bird population dynamics in the winter season: the case of the Mediterranean gull Larus melanocephalus* PhD thesis, Universitat de Barcelona, (2015).

178 Carboneras, C. *Selection of winter habitat by a gregarious long-lived seabird* MSc thesis, Universitat de Barcelona, (2009).

179 Duhem, C., Roche, P., Vidal, E. & Tatoni, T. Effects of anthropogenic food resources on yellow-legged gull colony size on Mediterranean islands. *Population ecology* **50**, 91-100 (2008).

180 Fasola, M. & Canova, L. Conservation of gull and tern colony sites in northeastern Italy, an internationally important bird area. *Colonial Waterbirds*, 59-67 (1996).

181 Prévot-Julliard, A.-C., Lebreton, J.-D. & Pradel, R. Re-evaluation of adult survival of Black-headed Gulls (Larus ridibundus) in presence of recapture heterogeneity. *The Auk*, 85-95 (1998).

182 Martínez-Abraín, A. *et al.* Unforeseen effects of ecosystem restoration on yellow-legged gulls in a small western Mediterranean island. *Environmental Conservation* **31**, 219-224 (2004).

183 Oro, D. Breeding biology and population dynamics of Slender-billed Gulls at the Ebro Delta (Northwestern Mediterranean). *Waterbirds* **25**, 67-77 (2002).

184 Oro, D. *et al.* Interference competition in a threatened seabird community: a paradox for a successful conservation. *Biological Conservation* **142**, 1830-1835 (2009).

185 Paterson, A. Seasonal evolution of the gull populations in Malaga, Spain. *Ardeola* **37**, 19-27 (1990).

186 Bazin, N. & Imbert, M. MEDITERRANEAN SHAG Phalacrocorax aristotelis desmarestii. Updated state of knowledge and conservation of the nesting populations of the Mediterranean Small Islands. . 19pp (2012).

187 Guyot, I. & Thibault, J.-C. Recent changes in the size of colonies of the Mediterranean Shag Phalacrocorax aristotelis desmarestii in Corsica, Western Mediterranean. *Seabird*, 10-19 (1996).

188 Zenatello, M., Baccetti, N. & Borghesi, F. Risultati dei censimenti degli uccelli acquatici svernanti in Italia. Distribuzione, stima e trend delle popolazioni nel 2001-2010. . *ISPRA, Serie Rapporti, 206/2014* (2014).

189 Sadoul, N., Johnson, A. R., Walmsley, J. G. & Levêque, R. Changes in the numbers and the distribution of colonial Charadriiformes breeding in the Camargue, Southern France. *Colonial waterbirds*, 46-58 (1996).

190 Sadoul, N. The importance of spatial scales in long-term monitoring of colonial Charadriiformes in southern France. *Colonial Waterbirds*, 330-338 (1997).

191 Martinez-Abraín, A., Oro, D. & Jiménez, J. The dynamics of a colonization event in the European shag: the roles of immigration and demographic stochasticity. *Waterbirds*, 97-102 (2001).

192 Sanz-Aguilar, A. *et al.* Living on the edge: demography of the slender-billed gull in the Western Mediterranean. *PloS one* **9**, e92674 (2014).

193 The Seabird Group. (ed S Wanless).

194 Mitchell, P., Newton, S., Ratcliffe, N. & Dunn, T. Seabird Populations of Britain and Ireland: results of the Seabird 2000 census (1998-2002). (London, 2004).

195 Young, H. G. Important Bird Areas in Africa and Associated Islands: Priority Sites for Conservation edited by Lincoln DC Fishpool & Michael I. Evans (2001), xvi+ 1,144 pp., Pisces Publications & BirdLife International, Newbury & Cambridge, UK. ISBN 1 874357 20 X (hbk),£ 55/$82.50. *Oryx* **36**, 305-307 (2002).

196 Telailia, S. *et al.* Demographic development of breeding populations of yellow-legged gull Larus michahellis Naumann, 1840 on the small islands and along the coastline of Numidia (North-Eastern Algeria). *Journal of Animal & Plant Sciences* **25**, 1160-1167 (2015).

197 UNEP-MAP-RAC/SPA. Adriatic Sea: Status and conservation of Seabirds. (Tunis, 2015).

198 Bonato, L., Trabucco, R. & Bon, M. Atti 7° Convegno Faunisti Veneti. Boll. Mus. St. Nat. Venezia, suppl. al vol. 66, pp. 292. (2016).

199 Scarton, F. & Valle, R. Long-term trends (1989-2013) in the seabird community breeding in the lagoon of Venice (Italy). *Rivista Italiana di Ornitologia* **85**, 19-28 (2016).

200 Kazantzidis, S. Status of the breeding population of Great Cormorants in Greece in 2012. 51-54 ( IUCN-Wetlands International Cormorant Research Group Report. Technical Report from DCE – Danish Centre for Environment and Energy, Aarhus Univ. , 2013).

201 Chokri, M. A. & Selmi, S. Nesting phenology and breeding performance of the Slender-billed Gull Chroicocephalus genei in Sfax salina, Tunisia. *Ostrich* **83**, 13-18 (2012).

202 Xirouchakis, S. M. *et al.* Status and diet of the European Shag (Mediterranean subspecies) Phalacrocorax aristotelis desmarestii in the Libyan Sea (south Crete) during the breeding season. *Marine Ornithology* **45**, 1-9 (2017).

203 Saravia Mullin, V. *et al.* in *Ecology and Conservation of Mediterranean Seabirds and other bird species under the Barcelona Convention-Proceedings of the 13th Medmaravis Pan-Mediterranean Symposium. Alghero (Sardinia).* 14-17.

204 Boschert, M. Population trends and status of Mediterranean Gull Larus melanocephalus as a breeding bird in Germany. *Proc 1st Int Mediterranean Gull Meet. EcoNum, Bailleul*, 43-46 (1999).

205 Panagiotopoulou, M., Kazantzidis, S., Katrana, E. & Alvanou, L. in *12th International Congress on the Zoogeography and ecology of Greece and Adjacent regions. Athens.*

206 Gerakēs, P. *Conservation and Management of Greek Wetlands: Proceedings of a Workshop on Greek Wetlands, Thessaloniki, Greece, 17-21 April, 1989*. Vol. 3 (IUCN, 1992).

207 Arizaga, J., Herrero, A., Aldalur, A., Cuadrado, J. F. & Oro, D. Effect of Pre-Fledging Body Condition on Juvenile Survival in Yellowlegged Gulls Larus michahellis. *Acta Ornithologica* **50**, 139-147 (2015).

208 Cam, E., Oro, D., Pradel, R. & Jimenez, J. Assessment of hypotheses about dispersal in a long‐lived seabird using multistate capture–recapture models. *Journal of Animal Ecology* **73**, 723-736 (2004).

209 Oro, D., Ruiz, X., Jover, L., Pedrocchi, V. & González-Solís, J. Diet and adult time budgets of Audouin's Gull Larus audouinii in response to changes in commercial fisheries. *Ibis* **139**, 631-637 (1997).

210 Goutner, V. in *Mediterranean Marine Avifauna* 431-447 (Springer, 1986).

211 Goutner, V. The diet of Mediterranean Gull (Larus melanocephalus) chicks at fledging. *Journal für Ornithologie* **135**, 193-201 (1994).

212 Talmat-Chaouchi, N., Boukhemza, M. & Moulai, R. Comparative analysis of the Yellow-legged Gull’s (Larus michahellis (Naumann, 1840)) trophic ecology in two colonies of the Central Coast of Algeria. *Zoology and Ecology* **24**, 324-331 (2014).

213 Cosolo, M., Privileggi, N., Cimador, B. & Sponza, S. Dietary changes of Mediterranean Shags Phalacrocorax aristotelis desmarestii between the breeding and post-breeding seasons in the upper Adriatic Sea. *Bird Study* **58**, 461-472 (2011).

214 Morat, F. *et al.* Diet of the mediterranean european shag, Phalacrocorax aristotelis desmarestii, in a northwestern mediterranean area: a competitor for local fisheries? *Scientific Reports of the Port-Cros National Park* **28**, 113-132 (2014).

215 Goutner, V., Papakostas, G. & Economidis, P. S. Diet and growth of great cormorant (Phalacrocorax carbo) nestlings in a Mediterranean estuarine environment (Axios Delta, Greece). *Israel Journal of Ecology and Evolution* **43**, 133-148 (1997).

216 Van Eerden, M. & Munsterman, M. in *Mediterranean Marine Avifauna* 123-141 (Springer, 1986).

217 Dies, J. I. & Dies, B. Breeding biology and colony size of Sandwich Tern at L'Albufera de Valencia (Western Mediterranean). *Ardeola* **51**, 431-435 (2004).

218 Oro, D., Bertolero, A., Vilalta, A. M. & López, M. A. The biology of the Little Tern in the Ebro Delta (northwestern Mediterranean). *Waterbirds* **27**, 434-440 (2004).

219 Sánchez, J., del Viejo, A. M., Corbacho, C., Costillo, E. & Fuentes, C. Status and trends of Gull-billed Tern Gelochelidon nilotica in Europe and Africa. *Bird Conservation International* **14**, 335-351 (2004).

220 Hamza, A. *et al.* in *Ecology and conservation of Mediterranean seabirds and other bird species under the Barcelona Convention: update and progress: proceedings of the 13th Medmaravis Pan-Mediterranean Symposium, Alghero, Sardinia, Italy.* 14-17.

221 Feltrup-Azafzaf, C. *et al.* Bulletin of the network “Mediterranean Waterbirds”, No. 2. AAO, ONCFS, Tour du Valat., (2014).

222 Hamza, A. *et al.* Migration flyway of the Mediterranean breeding Lesser Crested Tern Thalasseus bengalensis emigratus. *Ostrich* **88**, 53-58 (2017).

223 Casale, P. & Margaritoulis, D. *Sea turtles in the Mediterranean: distribution, threats and conservation priorities*. (IUCN, 2010).

224 Canbolat, A. F. A review of sea turtle nesting activity along the Mediterranean coast of Turkey. *Biological Conservation* **116**, 81-91 (2004).

225 Margaritoulis, D. *et al.* Loggerhead turtles in the Mediterranean Sea: present knowledge and conservation perspectives. *Loggerhead Sea Turtles (editors: AB Bolten, BE Witherington). Smithsonian Institution Press, Washington DC* (2003).

226 Margaritoulis, D. & Rees, A. F. The loggerhead turtle, Caretta caretta, population nesting in Kyparissia Bay, Peloponnesus, Greece: results of beach surveys over seventeen seasons and determination of the core nesting habitat. *Zoology in the Middle East* **24**, 75-90 (2001).

227 Mingozzi, T. *et al.* *Discovery of a regular nesting area of loggerhead turtle Caretta caretta in southern Italy: a new perspective for national conservation*. (Springer, 2008).

228 Margaritoulis, D. Nesting activity and reproductive output of loggerhead sea turtles, Caretta caretta, over 19 seasons (1984–2002) at Laganas Bay, Zakynthos, Greece: the largest rookery in the Mediterranean. *Chelonian Conservation and Biology* **4**, 916-929 (2005).

229 Lauriano, G., Panigada, S., Casale, P., Pierantonio, N. & Donovan, G. Aerial survey abundance estimates of the loggerhead sea turtle Caretta caretta in the Pelagos Sanctuary, northwestern Mediterranean Sea. *Mar Ecol Prog Ser* **437**, 291-302 (2011).

230 Groombridge, B. *Marine turtles in the Mediterranean: distribution, population status, conservation*. (Council of Europe, 1990).

231 Camiñas, J. A. Sea turtles of the Mediterranean Sea: population dynamics, sources of mortality and relative importance of fisheries impacts. *FAO fisheries report*, 27-84 (2004).

232 Broderick, A. C., Glen, F., Godley, B. J. & Hays, G. C. Estimating the number of green and loggerhead turtles nesting annually in the Mediterranean. *Oryx* **36**, 227-235 (2002).

233 Bentivegna, F. *et al.* Loggerhead turtle (*Caretta caretta*) nests at high latitudes in Italy: a call for vigilance in the Western Mediterranean. *Chelonian Conservation and Biology* **9**, 283-289 (2010).

234 Gomez de Segura, A., Tomas, J., Pedraza, S., Crespo, E. & Raga, J. Preliminary patterns of distribution and abundance of loggerhead sea turtles, Caretta caretta, around Columbretes Islands Marine Reserve, Spanish Mediterranean. *Marine Biology* **143**, 817-823 (2003).

235 Gómez de Segura, A., Tomás, J., Pedraza, S., Crespo, E. & Raga, J. Abundance and distribution of the endangered loggerhead turtle in Spanish Mediterranean waters and the conservation implications. *Animal Conservation* **9**, 199-206 (2006).

236 Casale, P. & Heppell, S. S. How much sea turtle bycatch is too much? A stationary age distribution model for simulating population abundance and potential biological removal in the Mediterranean. *Endangered Species Research* **29**, 239-254 (2016).

237 Türkozan, O. & Yilmaz, C. Loggerhead turtles, Caretta caretta, at Dalyan Beach, Turkey: nesting activity (2004–2005) and 19-year abundance trend (1987–2005). *Chelonian Conservation and Biology* **7**, 178-187 (2008).

238 Olgun, K. *et al.* Nesting activity of sea turtles, Caretta caretta (Linnaeus, 1758) and Chelonia mydas (Linnaeus, 1758)(Reptilia, Cheloniidae), at Patara Beach (Antalya, Turkey) over four nesting seasons. *Turkish Journal of Zoology* **40**, 215-222 (2016).

239 Casale, P. *et al.* Annual survival probabilities of juvenile loggerhead sea turtles indicate high anthropogenic impact on Mediterranean populations. *Aquatic Conservation: Marine and Freshwater Ecosystems* **25**, 690-700 (2015).

240 Hatase, H. & Tsukamoto, K. Smaller longer, larger shorter: energy budget calculations explain intrapopulation variation in remigration intervals for loggerhead sea turtles (Caretta caretta). *Canadian Journal of Zoology* **86**, 595-600 (2008).

241 Bjorndal, K. A. in *The biology of sea turtles* 199-231 (CRC press, 2017).

242 Bjorndal, K. A. Nutritional ecology of sea turtles. *Copeia*, 736-751 (1985).

243 Cardona, L., Álvarez de Quevedo, I., Borrell, A. & Aguilar, A. Massive consumption of gelatinous plankton by Mediterranean apex predators. *PloS one* **7**, e31329 (2012).

244 Godley, B., Smith, S., Clark, P. & Taylor, J. Molluscan and crustacean items in the diet of the loggerhead turtle, Caretta caretta (Linnaeus, 1758)[Testudines: Chelonidae] in the eastern Mediterranean. *Journal of Molluscan Studies* **63**, 474-476 (1997).

245 Tomas, J., Aznar, F. & Raga, J. Feeding ecology of the loggerhead turtle Caretta caretta in the western Mediterranean. *Journal of Zoology* **255**, 525-532 (2001).

246 Burgess, E. *et al.* Non-target by-catch in the Maltese bluefin tuna (Thunnus thynnus) longline fishery (Central Mediterranean). (2010).

247 Akyol, O., Ceyhan, T. & Erdem, M. Turkish pelagic gillnet fishery for swordfish and incidental catches in the Aegean Sea. *J. Black Sea/Mediterranean Environment* **18**, 188-196 (2012).

248 Báez, J. C., Macías, D., García-Barcelona, S. & Real, R. Interannual differences for sea turtles bycatch in Spanish longliners from Western Mediterranean Sea. *The Scientific World Journal* **2014** (2014).

249 Casale, P. Sea turtle by‐catch in the Mediterranean. *Fish and fisheries* **12**, 299-316 (2011).

250 Lucchetti, A. & Sala, A. An overview of loggerhead sea turtle (Caretta caretta) bycatch and technical mitigation measures in the Mediterranean Sea. *Reviews in Fish Biology and Fisheries* **20**, 141-161 (2010).

251 FAO. The State of Mediterranean and Black Fisheries. 134 pp. (Rome, Italy, 2016).

252 Kasparek, M. & Baran, İ. *Marine turtles, Turkey: Status survey 1988 and recommendations for conservation and management*. (World Wide Fund for Nature, 1989).

253 Kasparek, M., Godley, B. J. & Broderick, A. C. Nesting of the green turtle, Chelonia mydas, in the Mediterranean: a review of status and conservation needs. *Zoology in the Middle East* **24**, 45-74 (2001).

254 Rees, A. F., Saad, A. & Jony, M. Discovery of a regionally important green turtle Chelonia mydas rookery in Syria. *Oryx* **42**, 456-459 (2008).

255 Wabnitz, C. C. *et al.* Ecosystem structure and processes at Kaloko Honokhau, focusing on the role of herbivores, including the green sea turtle Chelonia mydas, in reef resilience. *Marine Ecology Progress Series* **420**, 27-44 (2010).

256 Bjorndal, K. A. Nutrition and grazing behavior of the green turtle Chelonia mydas. *Marine Biology* **56**, 147-154 (1980).

257 Lazar, B., Žuljević, A. & Holcer, D. Diet composition of a green turtle, Chelonia mydas, from the Adriatic Sea. *Natura Croatica: Periodicum Musei Historiae Naturalis Croatici* **19**, 263-271 (2010).

258 De Maddalena, A. SUPPLEMENTI Lo squalo bianco nel Mediterraneo. *Rivista Marittima* **143** (2010).

259 Echwikhi, K., Saidi, B., Bradai, M. & Bouain, A. Preliminary data on elasmobranch gillnet fishery in the Gulf of Gabès, Tunisia. *Journal of Applied Ichthyology* **29**, 1080-1085 (2013).

260 Storai, T., ZUFFA, M., CELONA, A. & DE MADDALENA, A. Historical and contemporary presence of the porbeagle, *Lamna nasus* (Bonnaterre, 1788) in Italian waters (Mediterranean Sea). *Annales, Series historia naturalis* **15**, 195-202 (2005).

261 Valeiras, J., de la Serna, J., Macías, D. & Alot, E. Nuevos datos científicos sobre desembarcos de especies asociadas realizados por la flota española de palangre de superficie en el Mediterráneo en 1999 y 2000. *Collective Volumen Scientific Papers ICCAT* **55**, 149-153 (2003).

262 Megalofonou, P. Incidental catch and estimated discards of pelagic sharks from the swordfish and tuna fisheries in the Mediterranean Sea. *Fishery Bulletin* **103**, 620-634 (2005).

263 Pauly, D. On the interrelationships between natural mortality, growth parameters, and mean environmental temperature in 175 fish stocks. *Journal du Conseil* **39**, 175-192 (1980).

264 Palomares, M. L. D. & Pauly, D. Predicting food consumption of fish populations as functions of mortality, food type, morphometrics, temperature and salinity. *Marine and freshwater research* **49**, 447-453 (1998).

265 De Maddalena, A. & Heim, W. *Mediterranean great white sharks: a comprehensive study including all recorded sightings*. (McFarland, 2012).

266 Barría, C., Coll, M. & Navarro, J. Unravelling the ecological role and trophic relationships of uncommon and threatened elasmobranchs in the western Mediterranean Sea. *Marine Ecology Progress Series* **539**, 225-240 (2015).

267 Kabasakal, H. On the occurrence of the blue shark, Prionace glauca (Chondrichthyes: Carcharhinidae), off Turkish coast of northern Aegean Sea. *Marine Biodiversity Records* **3** (2010).

268 Storai, T. *et al.* Bycatch of large elasmobranchs in the traditional tuna traps (tonnare) of Sardinia from 1990 to 2009. *Fisheries Research* **109**, 74-79 (2011).

269 De la Serna, J., Valeiras, J., Ortiz, J. & Macias, D. Large Pelagic sharks as by-catch in the Mediterranean Swordfish Longline Fishery: some biological aspects. *NAFO SCR Doc* **2**, 137 (2002).

270 Ceyhan, T. & Akyol, O. On the Turkish surface longline fishery targeting swordfish in the Eastern Mediterranean Sea. (2014).

271 Notarbartolo di Sciara, G. *et al.* The Devil We Don't Know: Investigating Habitat and Abundance of Endangered Giant Devil Rays in the North-Western Mediterranean Sea. *PloS one* **10**, e0141189 (2015).

272 Fortuna, C. M. *et al.* Summer distribution and abundance of the giant devil ray (Mobula mobular) in the Adriatic Sea: baseline data for an iterative management framework. *Scientia Marina* **78**, 227-237 (2014).

273 Mancusi, C. *et al.* On the presence of basking shark (Cetorhinus maximus) in the Mediterranean Sea. *Cybium* **29**, 399-405 (2005).

274 Lauriano, G. & Panigada, S. Aerial survey in the Pelagos Sanctuary for the Management and Conservation of the protected species. *Biologia marina mediterranea* **17**, 43-46 (2010).

275 Grémillet, D. *et al.* Ocean sunfish as indicators for the ‘rise of slime’. *Current Biology* **27**, R1263-R1264 (2017).

276 Celona, A. Catture ed avvistamenti di mòbula, Mobula mobular (Bonnaterre, 1788) nelle acque dello Stretto di Messina. *Annales* **14**, 11-18 (2004).

277 Quetglas, A. *et al.* Common spiny lobster (Palinurus elephas Fabricius 1787) fisheries in the western Mediterranean: A comparison of Spanish and Tunisian fisheries. Pesqueries de llagosta roja (Palinurus elephas Fabricius 1787) al Mediterrani Occidental: comparació de. *Bolletí de la Societat d'Història Natural de les Balears* **47**, 63-80 (2004).

278 Rouyer, T., Kimoto, A., Kell, L., Walter, J.F., Lauretta, M., Zarrad, R., Ortiz, M., Palma, C., Arrizabalaga, H., Sharma, R., Kitakado, T., Abid, N. Preliminary 2017 stock assessment results for the Eastern and Mediterranean Atlantic bluefin tuna stock. *Collect. Vol. Sci. Pap. ICCAT* **74**, 3234-3275 (2018).

279 Battaglia, P. *et al.* Feeding habits of the Atlantic bluefin tuna, Thunnus thynnus (L. 1758), in the central Mediterranean Sea (Strait of Messina). *Helgoland Marine Research* **67**, 97-107 (2013).

280 De la Serna, J. *et al.* Preliminary study on the feeding of bluefin tuna (Thunnus thynnus) in the Mediterranean and the Strait of Gibraltar area. *Collect. Vol. Sci. Pap. ICCAT* **68**, 115-132 (2012).

281 Morovic, D. in *Proceedings and Technical Papers of the General Fisheries Council for the Mediterranean.* 155-157.

282 Orsi Relini, L., Garibaldi, F., Cima, C. & Palandri, G. Feeding of the swordfish, the bluefin and other pelagic nekton in the western Ligurian Sea. *Collect. Vol. Sci. pap. ICCAT* **44**, 283-286 (1995).

283 Sanz Brau, A. Sur la nourriture des jeunes thons rouges Thunnus thynnus (L. 1758) des côtes du Golfe de Valence. *Rapp Comm Int Expl Sci Mer Médit* **32**, 274 (1990).

284 Sinopoli, M. *et al.* Diet of young‐of‐the‐year bluefin tuna, Thunnus thynnus (Linnaeus, 1758), in the southern Tyrrhenian (Mediterranean) Sea. *Journal of Applied Ichthyology* **20**, 310-313 (2004).

285 Sarà, G. & Sarà, R. Feeding habits and trophic levels of bluefin tuna Thunnus thynnus of different size classes in the Mediterranean Sea. *Journal of applied Ichthyology* **23**, 122-127 (2007).

286 Karakulak, F., Salman, A. & Oray, I. Diet composition of bluefin tuna (Thunnus thynnus L. 1758) in the Eastern Mediterranean Sea, Turkey. *Journal of Applied Ichthyology* **25**, 757-761 (2009).

287 Tsagarakis, K., Palialexis, A. & Vassilopoulou, V. Mediterranean fishery discards: review of the existing knowledge. *ICES Journal of Marine Science*, doi: doi:10.1093/icesjms/fst074 (2013).

288 FAO. The State of Mediterranean and Black Sea Fisheries. 172 (Rome, 2018).

289 ICCAT, S. (Madrid, Spain, September, 2006).

290 Chalabi, A. & Ifrene, F. Le régime alimentaire hivernal de l’espadon Xiphias gladius L. pêche pres des côtes est de L’Algerie. *SCRS/92/91 ICCAT* **40**, 162 (1992).

291 Navarro, J., Albo-Puigserver, M., Serra, P. E., Sáez-Liante, R. & Coll, M. Trophic strategies of three predatory pelagic fish coexisting in the north-western Mediterranean Sea over different time spans. *Estuarine, Coastal and Shelf Science* **246**, 107040 (2020).

292 Peristeraki, P., Tserpes, G. & Lefkaditou, E. What cephalopod remains from Xiphias gladius stomachs can imply about predator‐prey interactions in the Mediterranean Sea? *Journal of Fish Biology* **67**, 549-554 (2005).

293 Romeo, T., Consoli, P., Castriota, L. & Andaloro, F. An evaluation of resource partitioning between two billfish, Tetrapturus belone and Xiphias gladius, in the central Mediterranean Sea. *Journal of the Marine Biological Association of the United Kingdom* **89**, 849-857 (2009).

294 Salman, A. The role of cephalopods in the diet of swordfish (Xiphias gladius Linnaeus, 1758) in the Aegean Sea (Eastern Mediterranean). *Bulletin of Marine Science* **74**, 21-29 (2004).

295 Sacco, F. *et al.* The Mediterranean Sea hosts endemic haplotypes and a distinct population of the dolphinfish Coryphaena hippurus Linnaeus, 1758 (Perciformes, Coryphaenidae). *Fisheries research* **186**, 151-158 (2017).

296 Maggio, T. *et al.* Historical separation and present-day structure of common dolphinfish (Coryphaena hippurus) populations in the Atlantic Ocean and Mediterranean Sea. *ICES Journal of Marine Science* **76**, 1028-1038 (2019).

297 ICCAT. Report of the 2011 ICCAT South Atlantic and Mediterranean Albacore stock assessment sessions. (2012).

298 Castriota, L. *et al.* Diet of Coryphaena hippurus (Coryphaenidae) associated with FADs in the Ionian and southern Tyrrhenian seas. *Cybium* **31**, 435-441 (2007).

299 Massutí, E., Deudero, S., Sánchez, P. & Morales-Nin, B. Diet and feeding of dolphin (Coryphaena hippurus) in western Mediterranean waters. *Bulletin of Marine Science* **63**, 329-341 (1998).

300 Coetzee, D. Stomach content analysis of the leervis, Lichia amia (L.), from the Swartvlei system, southern Cape. *South African Journal of Zoology* **17**, 177-181 (1982).

301 Andaloro, F. & Pipitone, C. Food and feeding habits of the amberjack, Seriola dumerili in the Central Mediterranean Sea during the spawning season. *Cahiers de biologie marine* **38**, 91-96 (1997).

302 Matallanas, J., Casadevall, M., Carrasson, M., Bolx, J. & Fernandez, V. The food of Seriola dumerili (pisces: Carangidae) in the Catalan sea (western Mediterranean). *Journal of the Marine Biological Association of the United Kingdom* **75**, 257-260 (1995).

303 Mazzola, A., Lopiano, L., Sarà, G. & D’anna, G. Sistemi di pesca, cattura ed abitudini alimentary de Seriola dumerili (Risso 1810). *Natur. Sicil* **16**, 137-148 (1993).

304 Pipitone, C. & Andaloro, F. Food and feeding habits of juvenile greater amberjack, Seriola dumerili (Osteichthyes, Carangidae) in inshore waters of the central Mediterranean Sea. *Cybium (Paris)* **19**, 305-310 (1995).

305 Consoli, P. *et al.* Feeding habits of the albacore tuna Thunnus alalunga (Perciformes, Scombridae) from central Mediterranean Sea. *Marine Biology* **155**, 113-120 (2008).

306 Edelist, D., Sonin, O., Golani, D., Rilov, G. & Spanier, E. Spatiotemporal patterns of catch and discards of the Israeli Mediterranean trawl fishery in the early 1990 s: ecological and conservation perspectives. *Scientia Marina(Barcelona)* **75**, 641-652 (2011).

307 Giráldez, A., Torres, P., Quintanilla, L. & Baro, J. Anchovy (Engraulis encrasicolus) and sardine (Sardina pilchardus) Stock Assessment in the GFCM Geographical Sub-Area 01 (Northern Alboran Sea) and 06 (Northern Spain). *GFCM/SAC Working Group on Small Pelagic, Rome* (2005).

308 STECF. Review of scientific advice for 2012-Part 2 (STECF-11-09). Report No. 9279208039, (Luxembourg 2011).

309 Stergiou, K. *et al.* New fisheries-related data from the Mediterranean Sea. *Mediterranean Marine Science* **15**, 213-224 (2014).

310 Karachle, P. *Feeding ecology of the most important fish stock in the North Aegean Sea*, Ph. D. Thesis, Aristotle University of Thessaloniki, Department of Biology …, (2008).

311 Olaso, I. *et al.* Seasonal changes in the north-eastern Atlantic mackerel diet (Scomber scombrus) in the north of Spain (ICES Division VIIIc). *Journal of the Marine Biological Association of the United Kingdom* **85**, 415-418 (2005).

312 Sánchez, P., Demestre, M. & Martın, P. Characterisation of the discards generated by bottom trawling in the northwestern Mediterranean. *Fisheries Research* **67**, 71-80 (2004).

313 Sánchez, P. *et al.* Trawl catch composition during different fishing intensity periods in two Mediterranean demersal fishing grounds. *Scientia Marina* **71**, 765-773 (2007).

314 EC. Impact Assessment of Discard Reducing Policies: EU Discard Annex. (2011).

315 Damalas, D. *et al.* Historical discarding in Mediterranean fisheries: a fishers' perception. *ICES Journal of Marine Science* **72**, 2600-2608 (2015).

316 Lucchetti, A. *et al.* Small-scale driftnets in the Mediterranean: technical features, legal constraints and management options for the reduction of protected species bycatch. *Ocean & Coastal Management* **135**, 43-55 (2017).

317 Tsagarakis, K. *et al.* Old info for a new fisheries policy: Discard ratios and lengths at discarding in EU Mediterranean bottom trawl fisheries. *Frontiers in Marine Science* **4**, 99 (2017).

318 Cetinić, P., Škeljo, F. & Ferri, J. Discards of the commercial boat seine fisheries on Posidonia oceanica beds in the eastern Adriatic Sea. *Sci. Mar* **75**, 289-300 (2011).

319 Cresson, P., Ruitton, S., Ourgaud, M. & Harmelin-Vivien, M. Contrasting perception of fish trophic level from stomach content and stable isotope analyses: a Mediterranean artificial reef experience. *Journal of Experimental Marine Biology and Ecology* **452**, 54-62 (2014).

320 Kyrtatos, N. A. Contribution à la connaissance de la nourriture de Trachurus mediterraneus (Steind.) et de son influence sur les chaînes alimentaires de la mer Égée Centrale. *Rapp. Comm. Int. Expl. Sci. Mer Médit* **35**, 452-453 (1998).

321 Salem, M. B. Régime alimentaire de Trachurus trachurus (Linnaeus, 1758) et de T. mediterraneus (Steindachner, 1868),(poissons, téléosteens, carangidae) de la province Atlantic-Méditerranéenne. *Cybium (Paris)* **12**, 247-253 (1988).

322 Santic, M., Jardas, I. & Pallaoro, A. Feeding habits of Mediterranean horse mackerel, Trachurus mediterraneus (Carangidae), in the Central Adriatic Sea. *Cybium (Paris)* **27**, 247-253 (2003).

323 Moranta, J., Massutı́, E. & Morales-Nin, B. Fish catch composition of the deep-sea decapod crustacean fisheries in the Balearic Islands (western Mediterranean). *Fisheries Research* **45**, 253-264 (2000).

324 Sartor, P., Sbrana, M., Reale, B. & Belcari, P. Impact of the deep sea trawl fishery on demersal communities of the northern Tyrrhenian Sea (Western Mediterranean). *Journal of Northwest Atlantic Fishery Science* **31**, 275 (2003).

325 Fabi, G. & Grati, F. Small-scale fisheries in the maritime department of Ancona (Central Northern Adriatic Sea). *AdriaMed Tech. Doc*, 64-84 (2005).

326 Alsayes, A., Fattouh, S. & Abu-Enin, S. By-Catch and discarding of trawl fisheries at the Mediterranean coast of Egypt. *World Journal of Fish and Marine Sciences* **1**, 199-205 (2009).

327 Uzer, U., Yildiz, T. & Karakulak, F. S. Catch composition and discard of the boat seine in the İstanbul Strait (Turkey). *Turkish Journal of Zoology* **41**, 702-713 (2017).

328 Zengin, M. & Akyol, O. Description of by‐catch species from the coastal shrimp beam trawl fishery in Turkey. *Journal of Applied Ichthyology* **25**, 211-214 (2009).

329 Castriota, L., Campagnuolo, S. & Andaloro, F. Shrimp trawl fishery by-catch in the Straits of Sicily (central Mediterranean Sea). *Scientific Council Research Documents of the Northwest Atlantic Fisheries Organization, Serial* (2001).

330 Tzanatos, E., Somarakis, S., Tserpes, G. & Koutsikopoulos, C. Discarding practices in a Mediterranean small‐scale fishing fleet (Patraikos Gulf, Greece). *Fisheries Management and Ecology* **14**, 277-285 (2007).

331 Ceyhan, T., Akyol, O., Sever, T. M. & Kara, A. Diet composition of adult twaite shad (Alosa fallax) in the Aegean Sea (Izmir Bay, Turkey). *Journal of the Marine Biological Association of the United Kingdom* **92**, 601-604 (2012).

332 Sley, A., Jarboui, O., Ghorbel, M. & Bouain, A. Diet composition and food habits of Caranx rhonchus (Carangidae) from the Gulf of Gabes (central Mediterranean). *Journal of the Marine Biological Association of the United Kingdom* **88**, 831-836 (2008).

333 Dhieb, K., Ghorbel, M. & Bouain, A. Regime alimentaire de Pomatomus saltatrix (Teleostei, Pomatomidae) dans le Golfe de Gabes. *Tunisie. Rapp. Comm. int. Mer Médit* **36**, 259 (2001).

334 Campo, D., Mostarda, E., Castriota, L., Scarabello, M. & Andaloro, F. Feeding habits of the Atlantic bonito, Sarda sarda (Bloch, 1793) in the southern Tyrrhenian sea. *Fisheries Research* **81**, 169-175 (2006).

335 Fletcher, N., Batjakas, I. & Pierce, G. Diet of the A tlantic bonito S arda sarda (Bloch, 1793) in the Northeast Aegean Sea. *Journal of Applied Ichthyology* **29**, 1030-1035 (2013).

336 Lleonart, J. La pesquería de Cataluña y Valencia: descripción global y planteamiento de bases para su seguimiento. *Final Report. EC DG XIV Ref* (1990).

337 Navarro, J., Sáez-Liante, R., Albo-Puigserver, M., Coll, M. & Palomera, I. Feeding strategies and ecological roles of three predatory pelagic fish in the western Mediterranean Sea. *Deep Sea Research Part II: Topical Studies in Oceanography* **140**, 9-17 (2017).

338 Kalogirou, S., Mittermayer, F., Pihl, L. & Wennhage, H. Feeding ecology of indigenous and non‐indigenous fish species within the family Sphyraenidae. *Journal of fish biology* **80**, 2528-2548 (2012).

339 Grati, F. *et al.* The effect of monofilament thickness on the catches of gillnets for common sole in the Mediterranean small-scale fishery. *Fisheries Research* **164**, 170-177 (2015).

340 Machias, A. *et al.* Bottom trawl discards in the northeastern Mediterranean Sea. *Fisheries research* **53**, 181-195 (2001).

341 MEDIAS. Report of 5th meeting for MEDIterranean Acoustic Surveys in the framework of European Data Collection Framework (DCF)

(Sliema, Malta, 2012).

342 Bonanno, A. *et al.* Habitat selection response of small pelagic fish in different environments. Two examples from the oligotrophic Mediterranean Sea. *PLoS One* **9** (2014).

343 MEDIAS. Report of 3rd meeting for MEDiterranean Acoustic Surveys (MEDIAS) in the framework of European Data Collection Framework. (Capo Granitola, Sicily, 2010).

344 Bedairia, A. & Djebar, A. B. A preliminary analysis of the state of exploitation of the sardine, Sardina pilchardus (Walbaum, 1792), in the gulf of Annaba, East Algerian. *Animal biodiversity and conservation* **32**, 89-99 (2009).

345 GFCM. Scientific Advisory Committee on Fisheries (SAC). (Rome, Italy, 19–24 November 2018, 2018).

346 García, A. & Giraldez, A. Small pelagic fish research in the Mediterranean by the Spanish Institute of Oceanography: available data series for a climatic analysis. (Fuengirola, Spain, 2012).

347 GFCM. Scientific Advisory Committee on Fisheries (SAC). (Rome, Italy, 5–9 November 2012, 2012).

348 GFCM. Scientific Advisory Committee on Fisheries (SAC). (Rome, Italy, 23–28 November 2015, 2015).

349 STECF. Assessment of Mediterranean Sea stocks part I. (Luxembourg, 2010).

350 STECF. Assessment of Mediterranean Sea stocks part I (STECF 11-08). (Luxembourg, 2011).

351 STECF. Assessment of Mediterranean Sea stocks part I (STECF 12-19). (Luxembourg, 2012).

352 STECF. Assessment of Mediterranean Sea stocks part I (STECF 13-22). (Luxembourg, 2013).

353 STECF. Assessment of Mediterranean Sea stocks part II. (Luxembourg, 2010).

354 STECF. Assessment of Mediterranean Sea stocks part II (STECF-14-08). (Luxembourg, 2013).

355 STECF. Assessment of Mediterranean Sea stocks part II (STECF 11-14). (Luxembourg, 2011).

356 STECF. Assessment of Mediterranean Sea stocks part II (STECF 13-05). (Luxembourg, 2012).

357 Costalago, D., Palomera, I. & Tirelli, V. Seasonal comparison of the diets of juvenile European anchovy Engraulis encrasicolus and sardine Sardina pilchardus in the Gulf of Lions. *Journal of Sea Research* **89**, 64-72 (2014).

358 Costalago, D. & Palomera, I. Feeding of European pilchard (Sardina pilchardus) in the northwestern Mediterranean: from late larvae to adults. *Scientia Marina* **78**, 41-54 (2014).

359 Cunha, M. E., Garrido, S. & Pissarra, J. The use of stomach fullness and colour indices to assess Sardina pilchardus feeding. *Journal of the Marine Biological Association of the United Kingdom* **85**, 425-431 (2005).

360 Demirhindi, U. in *Proceedings and Technical Papers of the General Fisheries Council for the Mediterranean.*

361 Le Bourg, B. *et al.* Trophic niche overlap of sprat and commercial small pelagic teleosts in the Gulf of Lions (NW Mediterranean Sea). *Journal of Sea Research* **103**, 138-146 (2015).

362 Morote, E., Olivar, M. P., Villate, F. & Uriarte, I. A comparison of anchovy (Engraulis encrasicolus) and sardine (Sardina pilchardus) larvae feeding in the Northwest Mediterranean: influence of prey availability and ontogeny. *ICES Journal of Marine Science* **67**, 897-908 (2010).

363 Sever, T., Bayhan, B. & Taskavak, E. A preliminary study on the feeding regime of European pilchard (Sardina pilchardus Walbaum 1792) in Izmir Bay, Turkey, Eastern Aegean Sea. *NAGA, WorldFish Center Quarterly* **28**, 41-48 (2005).

364 Karachle, P. & Stergiou, K. Feeding and ecomorphology of three clupeoids in the N Aegean Sea. *Mediterranean Marine Science* **15**, 9-26 (2014).

365 Santojanni, A. *et al.* Stock assessment of sardine (Sardina pilchardus, Walb.) in the Adriatic Sea with an estimate of discards. *Scientia Marina* **69**, 603-617 (2005).

366 Pertierra, J. P. & Lleonart, J. NW Mediterranean anchovy fisheries. *Scientia Marina* **60**, 257-267 (1996).

367 Bacha, M. *et al.* Relationships between age, growth, diet and environmental parameters for anchovy (Engraulis encrasicolus L.) in the Bay of Bénisaf (SW Mediterranean, west Algerian coast). *Cybium* **34**, 47-57 (2010).

368 Plounevez, S. & Champalbert, G. Diet, feeding behaviour and trophic activity of the anchovy (Engraulis encrasicolus L.) in the Gulf of Lions (Mediterranean Sea). *Oceanologica Acta* **23**, 175-192 (2000).

369 Tudela, S. & Palomera, I. Trophic ecology of the European anchovy Engraulis encrasicolus in the Catalan Sea (northwest Mediterranean). *Marine Ecology Progress Series* **160**, 121-134 (1997).

370 Catalan, I. A. *et al.* Growth and feeding patterns of European anchovy (Engraulis encrasicolus) early life stages in the Aegean Sea (NE Mediterranean). *Estuarine, Coastal and Shelf Science* **86**, 299-312 (2010).

371 Tičina, V., Vidjak, O. & Kačič, I. Feeding of adult sprat, Sprattus sprattus, during spawning season in the Adriatic Sea. *Italian journal of zoology* **67**, 307-311 (2000).

372 Lomiri, S., Scacco, U., Mostarda, E. & Andaloro, F. Size‐related and temporal variation in the diet of the round sardinella, Sardinella aurita (Valenciennes, 1847), in the central Mediterranean Sea. *Journal of Applied Ichthyology* **24**, 539-545 (2008).

373 Madkour, F. F. Feeding ecology of the round sardinella, Sardinella aurita (Family: Clupeidae) in the Egyptian Mediterranean waters. *International Journal of Environmental Science and Engineering* **2**, 83-92 (2012).

374 Tsikliras, A. C., Torre, M. & Stergiou, K. I. Feeding habits and trophic level of round sardinella (Sardinella aurita) in the northeastern Mediterranean (Aegean Sea, Greece). *Journal of Biological Research* **3**, 67-75 (2005).

375 Harchouche, K., Maurin, C. & Zerouali-Khodja, F. Régime alimentaire de Spicara maena (centracanthidae), des eaux algériennes. *Bulletin de la Société zoologique de France* **134**, 125-143 (2009).

376 Mytilineou, C. *Contribution to the Biology of picarel Spicara flexuosa (Raf. 1810), in the Patraikos Gulf (Greece)*, PhD Thesis, University of Athens, (1987).

377 EC. Impact assessment of discard reducing policies. EU Discard Annex. (European Commission. Project: ZF0926_S10, 2011).

378 Castriota, L. *et al.* Crustacean fishery with bottom traps in an area of the southern Tyrrhenian Sea: species composition, abundance and biomass. *Mediterranean Marine Science* **5**, 15-22 (2004).

379 Aydin, M. Growth, reproduction and diet of pufferfish (Lagocephalus sceleratus Gmelin, 1789) from Turkey's Mediterranean sea coast. *Turkish Journal of Fisheries and Aquatic Sciences* **11** (2011).

380 Kalogirou, S. Ecological characteristics of the invasive pufferfish Lagocephalus sceleratus (Gmelin, 1789) in the eastern Mediterranean Sea–a case study from Rhodes. *Mediterranean Marine Science* **14**, 251-260 (2013).

381 Sever, T. M., Filiz, H., Bayhan, B., Taskavak, E. & Bilge, G. Food habits of the hollowsnout grenadier, Caelorinchus caelorhincus (Risso, 1810), in the Aegean Sea, Turkey. *Belgian Journal of Zoology* **138**, 81 (2008).

382 Macpherson, E. Resource partitioning in a Mediterranean demersal fish community. (1981).

383 Gramitto, M. Osservazioni sull’alimentazione di Antonogadus megalokynodon (Kolombatovic)(Pisces; Gadidae) nel Medio Adriatico. *Quad. Ist. Ric. Pesca Marit* **4**, 205-218 (1985).

384 Madurell, T. & Cartes, J. E. Temporal changes in feeding habits and daily rations of Hoplostethus mediterraneus in the bathyal Ionian Sea (eastern Mediterranean). *Marine Biology* **146**, 951-962 (2005).

385 Fanelli, E. & Cartes, J. E. Temporal variations in the feeding habits and trophic levels of three deep-sea demersal fishes from the western Mediterranean Sea, based on stomach contents and stable isotope analyses. *Marine Ecology Progress Series* **402**, 213-232 (2010).

386 Madurell, T. & Cartes, J. E. Trophic relationships and food consumption of slope dwelling macrourids from the bathyal Ionian Sea (eastern Mediterranean). *Marine Biology* **148**, 1325-1338 (2006).

387 Kebapcioglu, T. & Beğburs, C. Pufferfish species as discards in bottom trawl fisheries of Gulf of Antalya and Finike Bay, Turkey. *Natural and Engineering Sciences* (2017).

388 Yemisken, E., Dalyan, C. & Eryilmaz, L. Catch and discardfish species of trawl fisheries in the Iskenderun Bay (Northeastern Mediterranean) with emphasis on lessepsian and chondricthyan species. *Mediterranean Marine Science* **15**, 380-389 (2014).

389 Gorelli, G., Blanco, M., Sardà, F. & Carretón, M. Spatio-temporal variability of discards in the fishery of the deep-sea red shrimp Aristeus antennatus in the northwestern Mediterranean Sea: implications for management. *Scientia Marina* **80**, 79-88 (2016).

390 Gökçe, G., Bozaoğlu, A., Eryaşar, A. & Özbilgin, H. Discard reduction of trammel nets in the Northeastern Mediterranean prawn fishery. *Journal of Applied Ichthyology* **32**, 427-431 (2016).

391 Bernal, A., Olivar, M. P., Maynou, F. & de Puelles, M. L. F. Diet and feeding strategies of mesopelagic fishes in the western Mediterranean. *Progress in Oceanography* **135**, 1-17 (2015).

392 Battaglia, P. *et al.* Feeding habits of juvenile fishes belonging to three medusivorous species (Centrolophidae and Nomeidae) from the Strait of Messina (central Mediterranean Sea). *Marine Biology Research* **10**, 927-933 (2014).

393 Battaglia, P. *et al.* Diet of the spothead lanternfish Diaphus metopoclampus (Cocco, 1829)(Pisces: Myctophidae) in the central Mediterranean Sea. *Italian Journal of Zoology* **81**, 530-543 (2014).

394 Modica, L., Cartes, J. E. & Carrassón, M. Food consumption of five deep‐sea fishes in the Balearic Basin (western Mediterranean Sea): are there daily feeding rhythms in fishes living below 1000 m? *Journal of fish biology* **85**, 800-820 (2014).

395 Carrassón, M. & Matallanas, J. Preliminary data about the feeding habits of some deep‐sea Mediterranean fishes. *Journal of Fish Biology* **36**, 461-463 (1990).

396 Scotto, C., Costanzo, G., Fresi, E., Guglielmo, L. & Ianora, A. Feeding ecology and stranding mechanisms in two lanternfishes. *Hygophum benoiti and Myctophum punctatum, Mar. Ecol.: Progr. Ser.* **9**, 13-24 (1982).

397 Kaspiris, P., Argyriou, A. & Vertsiotis, A. Food of Hygophum benoiti (Pisces: Myctophidae) in Korinthiakos Gulf (Greece). *AQUA,* **1**, 21-24 (1994).

398 Battaglia, P. *et al.* Diet and trophic ecology of the lanternfish Electrona risso (Cocco 1829) in the Strait of Messina (central Mediterranean Sea) and potential resource utilization from the Deep Scattering Layer (DSL). *Journal of Marine Systems* **159**, 100-108 (2016).

399 Bernal, A. *et al.* Diet and trophic levels of myctophids, gonostomatids and hatchetfish in the Western Mediterranean. (2013).

400 Fanelli, E., Papiol, V., Cartes, J. E. & Rodriguez‐Romeu, O. Trophic ecology of Lampanyctus crocodilus on north‐west Mediterranean Sea slopes in relation to reproductive cycle and environmental variables. *Journal of Fish Biology* **84**, 1654-1688 (2014).

401 Tsagarakis, K., Vassilopoulou, V., Kallianiotis, A. & Machias, A. Discards of the purse seine fishery targeting small pelagic fish in the eastern Mediterranean Sea. *Scientia Marina* **76**, 561-572 (2012).

402 Negzaoui-Garali, N., Ben Salem, M. & Capape, C. Feeding habits of the black anglerfish, Lophius budegassa (Osteichthyes: Lophiidae), off the Tunisian coast (central Mediterranean). *Cahiers de biologie marine* **49**, 113 (2008).

403 López, N. *et al.* Feeding ecology of two demersal opportunistic predators coexisting in the northwestern Mediterranean Sea. *Estuarine, Coastal and Shelf Science* **175**, 15-23 (2016).

404 Stagioni, M., Montanini, S. & Vallisneri, M. Feeding habits of anglerfish, L ophius budegassa (Spinola, 1807) in the Adriatic Sea, north‐eastern Mediterranean. *Journal of Applied Ichthyology* **29**, 374-380 (2013).

405 Tsimenidis, N. C. Contribution to the study of the angler-fishes Lophius budegassa Spinola, 1807 and L. piscatorius L., 1758 in Greek seas. *Institute of Oceanography Fisheries Research (Athens) Special Publication* **4**, 180-190 (1980).

406 Županović, Š., Jardas, I., Alajbeg, M. & Fajfer, V. *Fauna i flora Jadrana: Jabučka kotlina*. (Logos, 1989).

407 Andaloro, F. & Arena, P. Contribution to the knowledge of the age, growth and feeding of hake Merluccius merluccius (L. 1758) in the Sicilian channel. *FAO Fisheries Report (FAO). no. 336.* (1985).

408 Bozzano, A., Recasens, L. & Sartor, P. Diet of the European hake Merluccius merluccius (Pisces: Merlucciidae) in the western Mediterranean (Gulf of Lions). (1997).

409 Papaconstantinou, C. & Caragitsou, E. The food of hake (Merluccius merluccius) in Greek Seas. *Vie et Milieu/Life & Environment*, 77-83 (1987).

410 Stergiou, K. I. & Karpouzi, V. S. Feeding habits and trophic levels of Mediterranean fish. *Reviews in fish biology and fisheries* **11**, 217-254 (2001).

411 Carpentieri, P., Colloca, F., Cardinale, M., Belluscio, A. & Ardizzone, G. D. Feeding habits of European hake (Merluccius merluccius) in the central Mediterranean Sea. *Fishery Bulletin* **103**, 411-416 (2005).

412 Froglia, C. in *Atti V Congresso Nazionale della Societá Italiana di Biologia Marina.* 327-341.

413 Mellon-Duval, C. *et al.* Trophic ecology of the European hake in the Gulf of Lions, northwestern Mediterranean Sea. *Scientia Marina* **81**, 7-18 (2017).

414 Jukic, S. Nutrition of the hake (Merluccius merluccius), bogue (Boops boops), striped mullet (Mullus barbatus) and pandora (Pagellus erythrinus) in the Bay of Kaštela. *Acta Adriatica* **14**, 3-40 (1972).

415 Deudero, S. & Morales-Nin, B. Occurrence of Polyprion americanus under floating objects in western Mediterranean oceanic waters, inference from stomach contents analysis. *Journal of the Marine Biological Association of the United Kingdom* **80**, 751-752 (2000).

416 Cau, A. & Manconi, P. Relationship of feeding, reproductive cycle and bathymetric distribution in Conger conger. *Marine Biology* **81**, 147-151 (1984).

417 Matić-Skoko, S. *et al.* The age, growth and feeding habits of the European conger eel, Conger conger (L.) in the Adriatic Sea. *Marine Biology Research* **8**, 1012-1018 (2012).

418 Anastasopoulou, A. *et al.* The diet and feeding ecology of Conger conger (L. 1758) in the deep waters of the Eastern Ionian Sea. *Mediterranean Marine Science* **14**, 365-368 (2013).

419 Bell, J. & Harmelin-Vivien, M. Fish fauna of French Mediterranean Posidonia oceanica seagrass meadows. II: feeding habits. *Tethys (Marseille)* **11**, 1-14 (1983).

420 Sallami, B., Ben Salem, M., Reynaud, C. & Capape, C. Diet of European conger eel Conger conger (Osteichthyes: Congridae), from the northeastern coast of Tunisia (central Mediterranean). *Cahiers de Biologie Marine* **56**, 253-262 (2015).

421 Derbal, F. & Kara, M. in *Second International Symposium on the Mediterranean Groupers, Nice, 13-17 May 2007.* 67-69.

422 Linde, M., Grau, A. M., Riera, F. & Massutí-Pascual, E. Analysis of trophic ontogeny in Epinephelus marginatus (Serranidae). *Cybium* **28**, 27-35 (2004).

423 Gracia López, V. & Castelló i Orvay, F. Food habits of groupers Epinephelus marginatus (Lowe, 1834) and Epinephelus costae (Steindachner, 1878) in the Mediterranean Coast of Spain. *Hidrobiológica* **15**, 27-34 (2005).

424 Gülşahin, A. & Soykan, O. Catch composition, length-weight relationship and discard ratios of commercial longline fishery in, the Eastern Mediterranean. (2017).

425 Morte, M. S., Redón, M. J. & Sanz‐Brau, A. Diet of Phycis blennoides (Gadidae) in relation to fish size and season in the western Mediterranean (Spain). *Marine Ecology* **23**, 141-155 (2002).

426 Artüz, M. L. The diet and food consumption of whiting Merlangius merlangus merlangus (Linne) 1758 in the Sea of Marmara. *Hidrobiologica, Publications Scientifiques* **1**, 23-26 (2005).

427 Petrakis, G. & Stergiou, K. in *Proceedings of the 2nd Panhellenic Symposium of Oceanography and Fisheries.* 558-564.

428 Papaconstantinou, C. *et al.* in *Technical Report* (National Centre for Marine Research Athens, 1989).

429 Biagi, F., de Ranieri, S. & Viva, C. Recruitment, length at first maturity and feeding of poor‐cod, Trisopterus minutus capelanus, in the northern Tyrrhenian Sea. *Italian Journal of Zoology* **59**, 87-93 (1992).

430 Morte, M. S., Redón, M. J. & Sanz‐Brau, A. Feeding habits of Trisopterus minutus capelanus (Gadidae) off the eastern coast of Spain (western Mediterranean). *Marine Ecology* **22**, 215-229 (2001).

431 Papaconstantinou, C. Investigation of the abundance and distribution of the demersal stocks of primary importance to the Greek Fishery in the North Aegean Sea (Greece). (2016).

432 Politou, C.-Y. & Papaconstantinou, C. Feeding ecology of Mediterranean poor cod, Trisopterus minutus capelanus (Lacepede), from the eastern coast of Greece. *Fisheries research* **19**, 269-292 (1994).

433 Gramitto, M. Feeding habits and estimation of daily ration of poor cod Trisopterus minutus capelanus (Gadidae) in the Adriatic Sea. *Cybium (Paris)* **23**, 115-130 (1999).

434 Moreno, T. & Castro, J. Community structure of the juvenile of coastal pelagic fish species in the Canary Islands waters [Spain]. *Scientia Marina* **59** 405-413 (1995).

435 Derbal, F. & Kara, M. H. Composition du régime alimentaire du bogue Boops boops (Sparidae) dans le golfe d’Annaba (Algérie). *Cybium* **32**, 325-333 (2008).

436 Morales-Nin, B. & Moranta, J. Life history and fishery of the common dentex (Dentex dentex) in Mallorca (Balearic Islands, western Mediterranean). *Fisheries Research* **30**, 67-76 (1997).

437 Bayhan, B., Sever, T. M. & Heral, O. Diet composition of the Morocco dentex: Dentex maroccanus Valenciennes, 1830 (Teleostei: Sparidae) in the central Turkish Aegean Sea. *Oceanological and Hydrobiological Studies* **46**, 133-139 (2017).

438 Relini, G., Relini, M., Torchia, G. & De Angelis, G. Trophic relationships between fishes and an artificial reef. *ICES Journal of Marine Science* **59**, S36-S42 (2002).

439 Rodríguez-Ruiz, S., Sánchez-Lizaso, J. L. & Ramos Esplá, A. A. Cambios estacionales en la dieta de Diplodus annularis (L., 1758) en el sudeste ibérico. *Boletín. Instituto Español de Oceanografía* **17**, 87-95 (2011).

440 Rosecchi, E. L'alimentation de Diplodus annualris, Diplodus sargus, Diplodus vulgaris et Sparus aurata (Pisces, Sparidae) dnas le golfe du Lion et les lagunes littorales. *Revue des Travaux de l'Institut des Pêches maritimes* **49**, 125-141 (1985).

441 Rosecchi, E. & Nouaze, Y. Comparaison de cinq indices alimentaires utilisés dans l'analyse des contenus stomacaux. *Revue des Travaux de l'Institut des Pêches maritimes* **49**, 111-123 (1985).

442 Chessa, L. A., Pais, A., Scardi, M., Serra, S. & Atzori, G. Osservazioni sul regime alimentare di Diplodus annularis (Linnaeus, 1758) nello stagno di Calich (Sardegna nord occidentale)= Observations on the feeding behaviour of Diplodus annularis (Linnaeus, 1758) in the Calich Lagoon (North Western Sardinia). *Biologia marina mediterranea* **11**, 573-576 (2004).

443 Fabi, G., Manoukian, S. & Spagnolo, A. Feeding behavior of three common fishes at an artificial reef in the northern Adriatic Sea. *Bulletin of Marine Science* **78**, 39-56 (2006).

444 Dulcic, J., Lipej, L., Glamuzina, B. & Bartulovic, V. Diet of Spondyliosoma cantharus and Diplodus puntazzo (Sparidae) in the eastern central adriatic. *Cybium* **30**, 115-122 (2006).

445 Sala, E. & Ballesteros, E. Partitioning of space and food resources by three fish of the genus Diplodus (Sparidae) in a Mediterranean rocky infralittoral ecosystem. *Marine Ecology Progress Series* **152**, 273-283 (1997).

446 Chaouch, H., Hamida, O. B. A.-B. H., Ghorbel, M. & Jarboui, O. Diet composition and food habits of Diplodus puntazzo (Sparidae) from the Gulf of Gabès (Central Mediterranean). *Journal of the Marine Biological Association of the United Kingdom* **93**, 2257-2264 (2013).

447 Taieb, A. H., Sley, A., Ghorbel, M. & Jarboui, O. Feeding habits of Sparus aurata (Sparidae) from the Gulf of Gabes (central Mediterranean). *Cah. Biol. Mar* **54**, 263-270 (2013).

448 Karachle, P. & Stergiou, K. An update on the feeding habits of fish in the Mediterranean Sea (2002-2015). *Mediterranean Marine Science* **18**, 43-52 (2017).

449 Hamida, N. B. H., Hamida, O. B. A.-B. H., Jarboui, O. & Missaoui, H. Diet composition and feeding habits of Lithognathus mormyrus (Sparidae) from the Gulf of Gabes (Central Mediterranean). *Journal of the Marine Biological Association of the United Kingdom* **96**, 1491-1498 (2016).

450 Kallianiotis, A., Torre, M. & Argyri, A. Age, growth, mortality, reproduction and feeding habits of the striped seabream, Lithognathus mormyrus (Pisces: Sparidae) in the coastal waters of the Thracian Sea, Greece. *Scientia Marina* **69**, 391-404 (2005).

451 Santic, M., Paladin, A. & Elez, G. Diet of striped sea bream Lithognathus mormyrus (Sparidae) from eastern central Adriatic Sea/Regime alimentaire du marbre, Lithognathus mormyrus (Sparidae), en mer Adriatique centrale. *Cybium, International Journal of Ichthyology* **34**, 345-353 (2010).

452 Lenfant, P. & Olive, C. Changements graduels du régime alimentaire des juvéniles d'oblade (Oblada melanura, Sparidae) lors du recrutement. *Cybium (Paris)* **22**, 203-210 (1998).

453 Pallaoro, A., Santic, M. & Jardas, I. Feeding habits of the saddled bream, Oblada melanura (Sparidae), in the Adriatic Sea. *Cybium* **27**, 261-268 (2003).

454 Pallaoro, A., Cetinić, P., Dulčić, J., Jardas, I. & Kraljević, M. Biological parameters of the saddlead bream Oblada melanura in the eastern Adriatic. *Fisheries research* **38**, 199-205 (1998).

455 Fehri-Bedoui, R., Mokrani, E. & Hassine, O. K. B. Feeding habits of Pagellus acarne (Sparidae) in the Gulf of Tunis, central Mediterranean. *Scientia Marina* **73**, 667-678 (2009).

456 Hamida, N. B. H., Abdallah, O. B. H. H.-B., Ghorbel, M., Jarboui, O. & Missaoui, H. The Feeding Habits of the Bluespotted Seabream, Pagrus caeruleostictus (Valenciennes, 1830), in the Gulf of Gabes (Central Mediterranean). *Reviews in Fisheries Science* **18**, 65-72 (2009).

457 Ardizzone, G. & Messina, A. Feeding habits of Pagellus erythrinus(L.)(Pisces, Sparidae) from the middle Tyrrhenian Sea. *RAPP. P.-V. REUN. CIESM.* **28**, 39-42 (1983).

458 Rosecchi, E. Regime alimentaire du pageot, Pagellus erythrinus, Linne 1758,(Pisces, Sparidae) dans le Golfe du Lion. *Cybium (Paris)* **7**, 17-29 (1983).

459 Andaloro, F., Prestipino Giarritta, S., Garcia, S. & Charbonnier, D. Contribution to the knowledge of the age, growth and feeding of pandora, Pagellus erythrinus (L. 1758) in the Sicilian Channel. (1985).

460 Caragitsou, E. & Papaconstantinou, C. Feeding habits of red pandora (Pagellus erythrinus) off the western coast of Greece. *Journal of Applied Ichthyology* **4**, 14-22 (1988).

461 Papaconstantinou, C. & Caragitsou, E. Feeding interaction between two sympatric species Pagrus pagrus and Phycis phycis around Kastellorizo Island (Dodecanese, Greece). *Fisheries Research* **7**, 329-342 (1989).

462 Labropoulou, M. & Papadopoulou-Smith, K.-N. Foraging behaviour patterns of four sympatric demersal fishes. *Estuarine, Coastal and Shelf Science* **49**, 99-108 (1999).

463 Verlaque, M. Note preliminaire sur le comportement alimentaire de Sarpa salpa (L.)(Sparidae) en Méditerranée. *Rapp. Comm. Int. Mer Médit* **29**, 193-196 (1985).

464 Bouchereau, J., Marques, C., Pereira, P., Guélorget, O. & Vergne, Y. Trophic characterization of the Prévost lagoon (Mediterranean Sea) by the feeding habits of the European eel Anguilla anguilla. *Cahiers de Biologie Marine* **47**, 133 (2006).

465 Capoccioni, F. *et al.* δ13C and δ15N in yellow and silver eels (Anguilla anguilla, 1758) from different Mediterranean local stocks and their variation with body size and growth. *Marine and Freshwater Research* (2021).

466 Abdalhamid, A. H., Ali, S. M., Ramadan, A., Elawad, A. N. & Buzaid, E. M. Food and feeding habits of the European eel Anguilla Anguilla (Linnaeus, 1758) in Umm Hufayan Lagoon, eastern Libya Mediterranean coast. *Bulletin de l’Institut Scientifique, Rabat*, 63-70 (2017).

467 Terrats, A., Petrakis, G. & Papaconstantinou, C. Feeding habits of Aspitrigla cuculus (L., 1758)(red gurnard), Lepidotrigla cavillone (Lac., 1802)(large scale gurnard) and Trigloporus lastoviza (Brunn., 1768)(rock gurnard) around Cyclades and Dodecanese Islands (E. Mediterranean). *Mediterranean Marine Science* **1**, 91-104 (2000).

468 Moreno-Amich, R. Feeding habits of red gurnard, Aspitrigla cuculus (L. 1758)(Scorpaeniformes, Triglidae), along the Catalan coast (northwestern Mediterranean). *Hydrobiologia* **228**, 175-184 (1992).

469 Colloca, F., Ardizzone, G. D. & Gravina, M. F. Trophic ecology of gurnards (Pisces: Triglidae). *Marine Life* **4**, 45-57 (1994).

470 Labropoulou, M. & Plaitis, W. Selective predation on small crustaceans by six demersal fish species in Iraklion Bay (Cretan Sea, north-eastern Mediterranean). *Oceanographic Literature Review* **2**, 120 (1997).

471 Castriota, L. *et al.* Trophic relationships among scorpaeniform fishes associated with gas platforms. *Helgoland Marine Research* **66**, 401-411 (2012).

472 Boudaya, L., Neifar, L., Taktak, A., Ghorbel, M. & Bouain, A. Diet of Chelidonichthys obscurus and Chelidonichthys lastoviza (Pisces: Triglidae) from the Gulf of Gabes (Tunisia). *Journal of Applied Ichthyology* **23**, 646-653 (2007).

473 Rogdakis, Y., Ramfos, A., Koukou, K., Dimitriou, E. & Katselis, G. Feeding habits and trophic level of sea bass (Dicentrarchus labrax) in the Messolonghi-Etoliko lagoons complex (Western Greece). *Journal of Biological Research* **13**, 13 (2010).

474 Khoury, C. in *International workshop on Posidonia oceanica beds 1, 1984.* (GIS Posidonie Publishing).

475 Madurell, T. & Labropoulou, M. in *Proceedings of the 6th Panhellenic Symposium of Oceanography and Fisheries.* 39-44.

476 Froglia, C. Observations on the feeding of Helicolenus dactylopterus (Delaroche)(Pisces, Scorpaenidae) in the Mediterranean Sea. *Rapp Comm Int Expl Scien Médit* **23**, 47-48 (1976).

477 Ouannes-Ghorbel, A. & Bouain, A. Régime alimentraire de Labrus viridis (Pisces, Labridae) des côtes sud de la Tunisie. *Rapport de la Commission international de la Mer Méditerranée* **37**, 416 (2004).

478 Froglia, C. Osservazioni sull'alimentazione di Sciaena umbra ed Umbrina cirrosa (Pisces, Sciaenidae) in prossimita di barriere artificiali in Adriatico. *Biol. Mar. Med.* **5**, 100-108 (1998).

479 Ordines, F., Valls, M. & Gouraguine, A. Biology, feeding, and habitat preferences of Cadenat's rockfish, Scorpaena loppei (Actinopterygii: Scorpaeniformes: Scorpaenidae), in the Balearic Islands (western Mediterranean). *Acta ichthyologica et Piscatoria* **42**, 21 (2012).

480 Morte, S., Redon, M. J. & Sanz-Brau, A. Diet of Scorpaena porcus and Scorpaena notata (Pisces: Scorpaenidae) in the western Mediterranean. *Cahiers de Biologie Marine* **42**, 333-344 (2001).

481 Harmelin‐Vivien, M., Kaim‐Malka, R., Ledoyer, M. & Jacob‐Abraham, S. Food partitioning among scorpaenid fishes in Mediterranean seagrass beds. *Journal of fish biology* **34**, 715-734 (1989).

482 Pallaoro, A. & Jardas, I. Food and feeding habits of Black Scorpionfish (Scorpaena porcus L. 1758) (PISCES, SCORPAENIDAE) along the Adriatic coast. *Acta Adriatica* **32**, 885-898 (1991).

483 Arculeo, M., Froglia, C. & Riggio, S. Food partitioning between Serranus scriba and Scorpaena porcus (Perciformes) on the infralittoral ground of the South Tyrrhenian Sea. *Cybium (Paris)* **17**, 251-258 (1993).

484 Bradai, M. & BOUAIN, A. Régime alimentaire de Scorpaena porcus et de S. scrofa (Teleostei, Scorpaenidae) du Golfe de Gabès, Tunisie. *Cybium (Paris)* **14**, 207-216 (1990).

485 Labropoulou, M. & Eleftheriou, A. The foraging ecology of two pairs of congeneric demersal fish species: importance of morphological characteristics in prey selection. *Journal of fish biology* **50**, 324-340 (1997).

486 Labropoulou, M., Tserpes, G. & Tsimenides, N. Age, Growth and Feeding Habits of the Brown Comber *Serranus hepatus* (Linnaeus, 1758) on the Cretan Shelf. *Estuarine, coastal and shelf science* **46**, 723-732 (1998).

487 Lundberg, B. & Golani, D. Diet adaptations of Lessepsian migrant rabbitfishes, Siganus luridus and S. rivulatus, to the algal resources of the Mediterranean coast of Israel. *Marine ecology* **16**, 73-89 (1995).

488 Wagué, A. *Biologie, écologie et dynamique de l'espèce seserranus hepatus (L., 1758)(poisson, serranidae) dans le Golfe de thermaikos (mer égée, Grèce)*, Université d'Aristote deThéssaloniki, (1997).

489 Bilecenoglu, M. Growth and feeding habits of the brown comber, Serranus hepatus (Linnaeus, 1758) in Izmir Bay, Aegean Sea. *Acta Adriatica* **50**, 105-110 (2009).

490 Khoury, C. *Ichtyofaune des herbiers de posidonies du Parc National de Port-Cros: composition, éthologie alimentaire et rôle dans le reseau trophique*, Aix-Marseille 2, (1987).

491 Ouannes-Ghorbel, A. & Bouain, A. The diet of the peacock wrasse, Symphodus (Crenilabrus) tinca (Labridae), in the southern coast of Tunisia. *Acta Adriatica: International journal of Marine Sciences* **47**, 175-182 (2006).

492 Preciado, I. *et al.* Food web functioning of the benthopelagic community in a deep-sea seamount based on diet and stable isotope analyses. *Deep Sea Research Part II: Topical Studies in Oceanography* **137**, 56-68 (2017).

493 Jardas, I. & Zupanovic, S. Feeding and some other properties of piper, Trigla lyra L., 1758,(Pisces, Triglidae) population in the South Adriatic pit area (Montenegro Coastal Area). *Stud. Mar* **13**, 167-187 (1983).

494 Beltrano, A., Cannizzaro, L., Vitale, S. & Milazzo, A. Preliminary study on the feeding habits of cleaver wrasse, Xyrichthys novacula (Pisces: Labridae) in the Strait of Sicily (Mediterranean Sea). *Electronic Journal of Ichthyology* **2**, 50-54 (2006).

495 Stergiou, K. & Fourtouni, H. Food habits, ontogenetic diet shift and selectivity in Zeus faber Linnaeus, 1758. *Journal of Fish Biology* **39**, 589-603 (1991).

496 Olguner, M. T. & Deval, M. C. Catch and selectivity of 40 and 44 mm trammel nets in small-scale fisheries in the Antalya Bay, Eastern Mediterranean. *Su Ürünleri Dergisi* **30**, 167-173 (2013).

497 Darnaude, A. M., Salen-Picard, C., Polunin, N. V. & Harmelin-Vivien, M. L. Trophodynamic linkage between river runoff and coastal fishery yield elucidated by stable isotope data in the Gulf of Lions (NW Mediterranean). *Oecologia* **138**, 325-332 (2004).

498 Fanelli, E., Badalamenti, F., D'Anna, G. & Pipitone, C. Diet and trophic level of scaldfish Arnoglossus laterna in the southern Tyrrhenian Sea (western Mediterranean): contrasting trawled versus untrawled areas. *Journal of the Marine Biological Association of the United Kingdom* **89**, 817-828 (2009).

499 Bayhan, B., Sever, T. M. & Taşkavak, E. Age, length-weight relationships and diet composition of scaldfish Arnoglossus laterna (Walbaum, 1792)(Pisces: Bothidae) in Izmir Bay (Aegean Sea). *Journal of Animal and Veterinary Advances* **7**, 924-929 (2008).

500 Passariello, M., Schintu, P., Belluscio, A. & Ardizzone, G. Growth and diet of Bothus podas (Pisces: Bothidae) in the central Mediterranean Sea. *Scientia Marina* **58**, 359-361 (1994).

501 Abid, S., Ouannes-Ghorbel, A., Jarboui, O. & Bouain, A. Diet composition and feeding habits of the wide-eyed flounder, Bothus podas, in the Gulf of Gabes (Tunisia). *Marine Biodiversity* **43**, 149-161 (2013).

502 Jardas, I. Citharus macrolepidotus (Bloch, 1787)(Pisces, Pleuronectiformes) Nourisment and length-weight relationships in the Adriatic Sea. *Acta Biologica Jugosl (e Ichthyol.)* **16**, 2 (1984).

503 de Juan, S., Cartes, J. E. & Demestre, M. Effects of commercial trawling activities in the diet of the flat fish Citharus linguatula (Osteichthyes: Pleuronectiformes) and the starfish Astropecten irregularis (Echinodermata: Asteroidea). *Journal of Experimental Marine Biology and Ecology* **349**, 152-169 (2007).

504 Sartor, P. & De Ranieri, S. Food and feeding habits of Lepidorhombus boscii (Pisces, Scophtalmidae) in the southern Tuscan Archipelago, Tyrrhenian Sea. *Vie et Milieu/Life & Environment*, 57-64 (1996).

505 Morte, S., Redón, M. J. & Sanz-Brau, A. Feeding ecology of two megrims Lepidorhombus boscii and Lepidorhombus whiffiagonis in the western Mediterranean (Gulf of Valencia, Spain). *Journal of the Marine Biological Association of the United Kingdom* **79**, 161-169 (1999).

506 Wyche, C. & Shackley, S. The feeding ecology of Pleuronectes platessa L., Limanda limanda (L.) and Scophthalmus rhombus (L.) in Carmarthen Bay, South Wales, UK. *Journal of fish biology* **29**, 303-311 (1986).

507 Molinero, A. & Flos, R. Influence of sex and age on the feeding habits of the common sole Solea solea. *Marine biology* **111**, 493-501 (1991).

508 Pellegrini, D. & Barghigiani, C. Feeding behaviour and mercury content in two flat fish in the northern Tyrrhenian Sea. *Marine pollution bulletin* **20**, 443-447 (1989).

509 Karachle, P. *et al.* New fisheries-related data from the Mediterranean Sea (April 2015). *Mediterranean Marine Science* **16**, 285-293 (2015).

510 Stergiou, K. Abundance‐depth relationship, condition factor and adaptive value of zooplanktophagy for red bandfish, Cepola macrophthalma. *Journal of fish biology* **42**, 645-660 (1993).

511 Sallami, B., Ben Salem, M., Reynaud, C. & Capape, C. Diet of Mediterranean moray, Muraena helena (Actinopterygii: Anguilliformes, Muraenidae), from the North-Eastern Tunisian coast (central Mediterranean). *Acta Ichthyologica et Piscatoria* **44** (2014).

512 Matić-Skoko, S. *et al.* Food preferences of the Mediterranean moray eel, Muraena helena (Pisces: Muraenidae), in the southern Adriatic Sea. *Marine Biology Research* **10**, 807-815 (2014).

513 Casadevall, M., Matallanas García, J. & Bartolí, T. Feeding habits of Ophichthus rufus (Anguilliformes, Ophichthidae) in the western Mediterranean. *© Cybium: International Journal of Ichthyology, 1994, vol. 18, núm. 4, p. 431-440* (1994).

514 Golani, D. The biology of the Red Sea migrant, Saurida undosquamis in the Mediterranean and comparison with the indigenous confamilial Synodus saurus (Teleostei: Synodontidae). *Hydrobiologia* **271**, 109-117 (1993).

515 Esposito, V. *et al.* Diet of Atlantic lizardfish, Synodus saurus (Linnaeus, 1758)(Pisces: Synodontidae) in the central Mediterranean Sea. *Scientia Marina* **73**, 369-376 (2009).

516 Morte, S., Redon, M. & Sanz-Brau, A. Feeding habits of Trachinus draco off the eastern coast of Spain (Western Mediterranean). *Vie et Milieu/Life & Environment*, 287-291 (1999).

517 Sanz, A. (Pesq, 1985).

518 Jaramillo Londoño, Á. M. *Estudio de la biología trófica de cinco especies de peces bentónicos de la costa de Cullera. Relaciones con la acumulación de metales pesados*, Universitat Politècnica de València, (2010).

519 Bautista‐Vega, A., Letourneur, Y., Harmelin‐Vivien, M. & Salen‐Picard, C. Difference in diet and size‐related trophic level in two sympatric fish species, the red mullets Mullus barbatus and Mullus surmuletus, in the Gulf of Lions (north‐west Mediterranean Sea). *Journal of Fish Biology* **73**, 2402-2420 (2008).

520 Caragitsou, Ε. & Tsimenidis, N. Seasonal changes and comparative analysis of the food of the red mullet (Mullus Barbatus) in the Gulfs of Saronikos and Thermaikos. (2016).

521 Chérif, M. *et al.* Food and feeding habits of the red mullet, Mullus barbatus (Actinopterygii: Perciformes: Mullidae), off the northern Tunisian coast (central Mediterranean). *Acta Ichthyologica et Piscatoria* **41** (2011).

522 Esposito, V. *et al.* Diet and prey selectivity of the red mullet, Mullus barbatus (Pisces: Mullidae), from the southern Tyrrhenian Sea: the role of the surf zone as a feeding ground. *Marine Biology Research* **10**, 167-178 (2014).

523 Golani, D. Niche separation between colonizing and indigenous goatfish (Mullidae) along the Mediterranean coast of Israel. *Journal of fish biology* **45**, 503-513 (1994).

524 Papaconstantinou, C. & Caragitsou, E. in *Proceedings of the 2nd Panhellenic Symposium of Oceanography and Fisheries.* 577-583.

525 Vassilopoulou, V. & Papaconstantinou, C. Feeding habits of red mullet (Mullus barbatus) in a gulf in western Greece. *Fisheries research* **16**, 69-83 (1993).

526 El Bakali, M., Talbaoui, M. & Bendriss, A. Régime alimentaire du Rouget de roche (Mullus surmuletus L.)(Téléostéen, Mullidae) de la côte nord-ouest méditerranéenne du Maroc (région de M’diq). *Bulletin de l’Institut Scientifique, Rabat, section Sciences de la Vie* **32**, 87 (2010).

527 Arculeo, M., Pipitone, C. & Riggio, S. Aspetti del regime alimentare di Mullus surmuletus L.(Pisces, Mullidae) nel Golfo di Palermo. *Oebalia* **15**, 56-66 (1989).

528 La Mesa, M. *et al.* Feeding ecology of the transparent goby Aphia minuta (Pisces, Gobiidae) in the northwestern Adriatic Sea. *Scientia Marina* **72**, 99-108 (2008).

529 Kabasakal, H. Description of the feeding morphology and the food habits of four sympatric labrids (Perciformes, Labridae) from south-eastern Aegean Sea, Turkey. *Netherlands journal of zoology* **51**, 439-455 (2001).

530 Moreno, R. & Matallanas, J. Étude du régime alimentaire de Lepidotrigla cavillone (Lacepède, 1801)(Pisces, Triglidae) dans la Mer Catalane. *Cybium (Paris)* **7**, 93-103 (1983).

531 Caragitsou, E. & Papaconstantinou, C. Food and feeding habits of large scale gurnard, Lepidotrigla cavillone (Triglidae) in Greek Seas. *Cybium* **14**, 95-104 (1990).

532 Labropoulou, M. & Machias, A. Effect of habitat selection on the dietary patterns of two triglid species. *Marine Ecology Progress Series* **173**, 275-288 (1998).

533 Bouain, A., Ouannes-Ghorbel, A., Jarboui, O. & M’rabet, R. Régime alimentaire de symphodus (Crenilabrus) ocellatus des côtes sud de la Tunisie. (2003).

534 Urra, J. *et al.* Discard analysis and damage assessment in the wedge clam mechanized dredging fisheries of the northern Alboran Sea (W Mediterranean Sea). *Fisheries Research* **187**, 58-67 (2017).

535 Garnaud, J. *Monographie de l'Apogon méditerranéen, Apogon imberbis (Linné) 1758*. (1962).

536 Sever, T. M., Bayhan, B., Filiz, H., Taşkavak, E. & Bilge, G. Ege Denizi'nde dağılım gösteren beş derin deniz balığının diyet kompozisyonu. *Ege Journal of Fisheries and Aquatic Sciences* **30** (2013).

537 Carpentieri, P., Serpetti, N., Colloca, F., Criscoli, A. & Ardizzone, G. Food preferences and rhythms of feeding activity of two co‐existing demersal fish, the longspine snipefish, Macroramphosus scolopax (Linnaeus, 1758), and the boarfish Capros aper (Linnaeus, 1758), on the Mediterranean deep shelf. *Marine Ecology* **37**, 106-118 (2016).

538 Anastasopoulou, A. & Kapiris, K. Feeding ecology of the shortnose greeneye Chlorophthalmus agassizi Bonaparte, 1840 (Pisces: Chlorophthalmidae) in the eastern Ionian Sea (eastern Mediterranean). *Journal of Applied Ichthyology* **24**, 170-179 (2008).

539 Dulcic, J. Diet composition of young-of-the-year damselfish, Chromis chromis (Pomacentridae), from the eastern Adriatic Sea. *Cybium (Paris)* **31**, 95-96 (2007).

540 Pinnegar, J. K. & Polunin, N. V. Contributions of stable-isotope data to elucidating food webs of Mediterranean rocky littoral fishes. *Oecologia* **122**, 399-409 (2000).

541 Zander, C. Feeding ecology of littoral gobiid and blennioid fish of the Banyuls area (Mediterranean Sea) I. Main food and trophic dimension of niche and ecotope. *Vie et Milieu/Life & Environment* **32**, 1-10 (1982).

542 Filiz, H. & Toğulga, M. Age and growth, reproduction and diet of the black goby,(Gobius niger) from Aegean Sea, Turkey. (2009).

543 Carrassón, M. & Matallanas, J. Feeding habits of Alepocephalus rostratus (Pisces: Alepocephalidae) in the western Mediterranean Sea. *Journal of the Marine Biological Association of the United Kingdom* **78**, 1295-1306 (1998).

544 Carrassón, M. & Matallanas, J. Feeding habits of Cataetyx alleni (Pisces: Bythitidae) in the deep western Mediterranean. *Scientia Marina* **66**, 417-421 (2002).

545 Matallanas, J. Notes on the feeding habits of Epigonus denticulatus (Pisces, Apogonidae) in the Catalan sea (Western Mediterranean). *Vie et Milieu/Life & Environment* **32**, 77-81 (1982).

546 Relini Orsi, L. & Wurtz, M. Biology of Trachyrhynchus trachyrhynchus (Risso, 1810)(Osteichthyes, Macrouridae) during the first years of benthic life. *Rapport de la Commission Internationale de la Mer Méditerranée* **10**, 71-77 (1979).

547 Türker Çakır, D., Torcu Koç, H. & Erdoğan, Z. in *Proc. Of the Int. Workshop on Med. Cartilaginous Fish with Emphasis on South.-East. Med.* 14-16.

548 Gravino, F., Dimech, M. & Schembri, P. J. Feeding habits of the small-spotted catshark Scyliorhinus canicula (L., 1758) in the central Mediterranean. *Rapport Commission International de la Mer Mediterranee* **39**, 538 (2010).

549 Jardas, I. Supplement to the knowledge of ecology of some Adriatic cartilaginous fishes (Chondrichthyes) with special reference to their nutrition. *Acta Adriatica* **14**, 1-60 (1972).

550 Mulas, A., Bellodi, A., Pendugiu, A. & Cabiddu, S. Preliminary data on the trophic interactions between Scyliorhinus canicula (Scyliorhinidae) and Raja clavata (Rajidae) in the central-western Mediterranean. *Rapport de la Commission international de la Mer Méditerranée* **39**, 596 (2010).

551 Valls, M., Quetglas, A., Ordines, F. & Moranta, J. Feeding ecology of demersal elasmobranchs from the shelf and slope off the Balearic Sea (western Mediterranean). *Scientia Marina* **75**, 633-639 (2011).

552 Valls Mir, M. *Trophic Ecology in Marine Ecosystems from the Balearic Sea (Western Mediterraniean)* PhD thesis, Universitat de les Illes Balears, (2017).

553 Albo-Puigserver, M. *et al.* Feeding ecology and trophic position of three sympatric demersal chondrichthyans in the northwestern Mediterranean. *Marine Ecology Progress Series* **524**, 255-268 (2015).

554 Carrassón, M., Stefanescu, C. & Cartes, J. E. Diets and bathymetric distributions of two bathyal sharks of the Catalan deep sea (western Mediterranean). *Marine Ecology Progress Series*, 21-30 (1992).

555 Moura, T., Figueiredo, I., Bordalo-Machado, P. & Gordo, L. S. Feeding habits of Chimaera monstrosa L.(Chimaeridae) in relation to its ontogenetic development on the southern Portuguese continental slope. *Marine Biology Research* **1**, 118-126 (2005).

556 Torres, M., Gil, J. & Sobrino, I. in *Ecosystem Based Management and monitoring in the Deep Med. & N. Atlantic Conference.*

557 Eronat, E. G. T. Feeding ecology and trophic level of Chimaera monstrosa Linnaeus, 1758 (Holocephali: Chimaeridae) in the Eastern Mediterranean. *Zoology in the Middle East* **62**, 51-57 (2016).

558 Navarro, J., López, L., Coll, M., Barría, C. & Sáez-Liante, R. Short-and long-term importance of small sharks in the diet of the rare deep-sea shark Dalatias licha. *Marine Biology* **161**, 1697-1707 (2014).

559 Morato, T., Sola, E., Grós, M. P. & Menezes, G. Diets of thornback ray (Raja clavata) and tope shark (Galeorhinus galeus) in the bottom longline fishery of the Azores, northeastern Atlantic. *Fishery Bulletin* **101**, 590-602 (2003).

560 Celona, A., De Maddalena, A. & Romeo, T. Bluntnose sixgill shark, Hexanchus griseus (Bonnaterre, 1788), in the eastern north Sicilian waters. *Bollettino del Museo civico di Storia naturale di Venezia* **56**, 137-151 (2005).

561 Saidi, B., Enajjar, S., Bradai, M. N. & Bouain, A. Diet composition of smooth‐hound shark, Mustelus mustelus (Linnaeus, 1758), in the Gulf of Gabès, southern Tunisia. *Journal of Applied Ichthyology* **25**, 113-118 (2009).

562 Gračan, R., Mladineo, I. & Lazar, B. Insight into the diet composition and gastrointestinal parasite community of the common smooth-hound, Mustelus mustelus (Carcharhiniformes: Triakidae), in the northern Adriatic Sea. *Natura Croatica* **23** (2014).

563 Lipej, L., Mavric, B., Rešek, S., Cherif, M. & Capape, C. Food and feeding habits of the blackspotted smooth-hound, Mustelus punctulatus (Elasmobranchii: Carcharhiniformes: Triakidae), from the northern Adriatic. *Acta Ichthyologica et Piscatoria* **41** (2011).

564 Laptikhovsky, V., Arkhipkin, A. & Henderson, A. Feeding habits and dietary overlap in spiny dogfish Squalus acanthias (Squalidae) and narrowmouth catshark Schroederichthys bivius (Scyliorhinidae). *Journal of the Marine Biological Association of the United Kingdom* **81**, 1015-1018 (2001).

565 Kousteni, V. Population characteristics and heavy metal concentrations in benthic sharks from the eastern Mediterranean Sea. *Doctoral Thesis* (2015).

566 Jardas, I., Šantić, M., Nerlović, V. & Pallaoro, A. Diet of the smooth-hound Mustelus mustelus (Chondrichthyes: Triakidae) in the eastern Adriatic Sea. *Cybium* **31**, 459-464 (2007).

567 Santic, M., Paladin, A. & Agovic, A. Diet of common stingray, Dasyatis pastinaca (Chondrichthyes: Dasyatidae) in the eastern Adriatic Sea. *Cahiers de biologie marine* **52**, 349-356 (2011).

568 Ismen, A. Age, growth, reproduction and food of common stingray (Dasyatis pastinaca L., 1758) in Iskenderun Bay, the eastern Mediterranean. *Fisheries Research* **60**, 169-176 (2003).

569 Yıgın, C. & Ismen, A. Age, growth, reproduction and feed of longnosed skate, Dipturus oxyrinchus (Linnaeus, 1758) in Saros Bay, the north Aegean Sea. *Journal of Applied Ichthyology* **26**, 913-919 (2010).

570 Mulas, A. *et al.* Diet and feeding behaviour of longnosed skate Dipturus oxyrinchus. *Journal of Fish Biology* **86**, 121-138 (2015).

571 Yemışken, E., Forero, M. G., Megalofonou, P., Eryilmaz, L. & Navarro, J. Feeding habits of three Batoids in the Levantine Sea (north-eastern Mediterranean Sea) based on stomach content and isotopic data. *Journal of the Marine Biological Association of the United Kingdom* **98**, 89-96 (2018).

572 Jardas, I., Santic, M. & Pallaoro, A. Diet composition of the eagle ray, Myliobatisaquila (Chondrichthyes: Myliobatidae), in the Eastern Adriatic Sea. *Cybium (Paris)* **28**, 372-374 (2004).

573 Lipej, L., Mavrič, B., Paliska, D. & Capapé, C. Feeding habits of the pelagic stingray Pteroplatytrygon violacea (Chondrichthyes: Dasyatidae) in the Adriatic Sea. *Journal of the Marine Biological Association of the United Kingdom* **93**, 285-290 (2013).

574 Navarro, J., Coll, M., Preminger, M. & Palomera, I. Feeding ecology and trophic position of a Mediterranean endemic ray: consistency between sexes, maturity stages and seasons. *Environmental Biology of Fishes* **96**, 1315-1328 (2013).

575 Serena, F., Barone, M., Mancusi, C. & Abella, A. in *ICES Annual Science Conference.*

576 Capapé, C. & Quignard, J.-P. Contribution à la biologie des Rajidae des côtes tunisiennes: 6. Raja asterias Delaroche, 1809: Régime alimentaire. *Bull. Inst. Natl. Sci. Tech. Oceanogr.* **4**, 319-333 (1977).

577 Follesa, M. C. *et al.* Diet and feeding habits of two skate species, Raja brachyura and Raja miraletus (Chondrichthyes, Rajidae) in Sardinian waters (central‐western Mediterranean). *Italian Journal of Zoology* **77**, 53-60 (2010).

578 Catalano, B., Dalù, M., Scacco, U. & Vacchi, M. New biological data on Raja brachyura (Chondrichthyes, Rajidae) from around Asinara Island (NW Sardinia, Western Mediterranean). *Italian Journal of Zoology* **74**, 55-61 (2007).

579 Kadri, H., Saïdi, B., Marouani, S., Bradai, M. & Bouaïn, A. Food habits of the rough ray Raja radula (Chondrichthyes: Rajidae) from the Gulf of Gabès (central Mediterranean Sea). *Italian Journal of Zoology* **80**, 52-59 (2013).

580 Abdel-Aziz, S. Observations on the biology of the common torpedo (Torpedo torpedo, Linnaeus, 1758) and marbled electric ray (Torpedo marmorata, Risso, 1810) from Egyptian Mediterranean waters. *Marine and Freshwater Research* **45**, 693-704 (1994).

581 Jaramillo Londoño, Á. M., Cantos, G., Porras Castelló, R. & Bendito Durà, V. Composición de la dieta y estrategia alimentaria de cinco especies de peces bentónicos de la costa de Cullera (España). *Mediterránea*, 41pp (2011).

582 Pauly, D. Population dynamics of short-lived species, with emphasis on squids. *Northwest Atlantic Fisheries Organization (NAFO)* **9**, 101-106 (1984).

583 Pauly, D., Sambilay Jr, V. & Opitz, S. in *Trophic Models of Aquatic Ecosystems. V. Christensen and D. Pauly (eds.) ICLARM Conference Proceedings.* 236-225.

584 Quetglas, A., González, M., Carbonell, A. & Sánchez, P. Biology of the deep-sea octopus Bathypolypus sponsalis (Cephalopoda: Octopodidae) from the western Mediterranean Sea. *Marine Biology* **138**, 785-792 (2001).

585 Sanchez, P. Régime alimentaire d’Eledone cirrosa (Lamarck, 1798)(Mollusca, Cephalopoda) dans la mer Catalane. *Rapp. Comm. Int. Mer. Médit* **27**, 209-212 (1981).

586 Šifner, S. K. & Vrgoč, N. Diet and feeding of the musky octopus, Eledone moschata, in the northern Adriatic Sea. *Journal of the Marine Biological Association of the United Kingdom* **89**, 413-419 (2009).

587 Quetglas, A., González, M. & Franco, I. Biology of the upper-slope cephalopod Octopus salutii from the western Mediterranean Sea. *Marine Biology* **146**, 1131-1138 (2005).

588 Quetglas, A., Alemany, F., Carbonell, A., Merella, P. & Sánchez, P. Biology and fishery of Octopus vulgaris Cuvier, 1797, caught by trawlers in Mallorca (Balearic Sea, Western Mediterranean). *Fisheries Research* **36**, 237-249 (1998).

589 Quetglas, A., Ordines, F., Gonzalez, M. & Franco, I. Life history of the bathyal octopus Pteroctopus tetracirrhus (Mollusca, Cephalopoda) in the Mediterranean Sea. *Deep Sea Research Part I: Oceanographic Research Papers* **56**, 1379-1390 (2009).

590 Castro, B. & Guerra, A. The diet of Sepia officinalis (Linnaeus 1758) and Sepia elegans (D'Orbigny 1835)(Cephalopoda, Sepioidea) from the Ria de Vigo (Northwestern Spain). *Scientia Marina (Spain)* (1990).

591 Ambrose, R. F. & Nelson, B. V. Predation by Octopus vulgaris in the Mediterranean. *Marine Ecology* **4**, 251-261 (1983).

592 Ajana, R., Techetach, M. & Saoud, Y. Diet of Octopus vulgaris from the moroccan Mediterranean Coast. *Thalassas: An International Journal of Marine Sciences* **34**, 415-420 (2018).

593 Quetglas, A., Reñones, O. & Goñi, R. Trophic interactions among grouper (Epinephelus marginatus), octopus (Octopus vulgaris) and red lobster (Palinurus elephas) in the Western Mediterranean. *Rapp Comm Int Mer Médit* **36**, 310 (2001).

594 Rosas-Luis, R. & Sánchez, P. Food and feeding habits of Alloteuthis media in the Western Mediterranean Sea. *Marine Biology Research* **11**, 438-442 (2015).

595 Sánchez, P. Régimen alimentario de Illex coindetti (Verany, 1837) en el mar Catalán. (1982).

596 Martínez-Baena, F., Navarro, J., Albo-Puigserver, M., Palomera, I. & Rosas-Luis, R. Feeding habits of the short-finned squid Illex coindetii in the western Mediterranean Sea using combined stomach content and isotopic analysis. *Journal of the Marine Biological Association of the United Kingdom* **96**, 1235-1242 (2016).

597 Valls, M., Cabanellas-Reboredo, M., Uranga, I. & Quetglas, A. Feeding ecology of two squid species from the western Mediterranean. *Marine Ecology Progress Series* **531**, 207-219 (2015).

598 Pierce, G. J. *et al.* in *Essential Fish Habitat Mapping in the Mediterranean* 49-70 (Springer, 2008).

599 Pierce, G. J., Boyle, P. R., Hastie, L. C. & Santos, M. B. Diets of squid Loligo forbesi and Loligo vulgaris in the northeast Atlantic. *Fisheries research* **21**, 149-163 (1994).

600 Lordan, C., Burnell, G. & Cross, T. The diet and ecological importance of Illex coindetii and Todaropsis eblanae (Cephalopoda: Ommastrephidae) in Irish waters. *African Journal of Marine Science* **20** (1998).

601 Quetglas, A., Alemany, F., Carbonell, A., Merella, P. & Sanchez, P. Diet of the European flying squid Todarodes sagittatus (Cephalopoda: Ommastrephidae) in the Balearic Sea (western Mediterranean). *Journal of the Marine Biological Association of the United Kingdom* **79**, 479-486 (1999).

602 Quetglas, A., de Mesa, A., Ordines, F. & Grau, A. Life history of the deep-sea cephalopod family Histioteuthidae in the western Mediterranean. *Deep Sea Research Part I: Oceanographic Research Papers* **57**, 999-1008 (2010).

603 Brey, T. A collection of empirical relations for use in ecological modelling. *Naga the ICLARM quarterly* **22**, 24-28 (1999).

604 Brey, T. *Population dynamics in benthic invertebrates. In: A Virtual Handbook Version 01.2. Alfred Wegener Institute for Polar and Marine Research, Germany,* [*http://www.awi-bremerhaven.de/Benthic/Ecosystem/FoodWeb/Handbook/main.html*](http://www.awi-bremerhaven.de/Benthic/Ecosystem/FoodWeb/Handbook/main.html)*.*, 2001).

605 Purroy, A. *et al.* Bivalve trophic ecology in the Mediterranean: Spatio-temporal variations and feeding behavior. *Marine environmental research* **142**, 234-249 (2018).

606 Galimany, E., Ramón, M. & Ibarrola, I. Feeding behavior of the mussel Mytilus galloprovincialis (L.) in a Mediterranean estuary: a field study. *Aquaculture* **314**, 236-243 (2011).

607 Metaxatos, A. Population dynamics of the venerid bivalve Callista chione (L.) in a coastal area of the eastern Mediterranean. *Journal of sea research* **52**, 293-305 (2004).

608 Pernet, F. *et al.* Marine diatoms sustain growth of bivalves in a Mediterranean lagoon. *Journal of sea research* **68**, 20-32 (2012).

609 Moles, J. *et al.* As fast as a hare: colonization of the heterobranch Aplysia dactylomela (Mollusca: Gastropoda: Anaspidea) into the western Mediterranean. (2017).

610 Vizzini, S., Colombo, F., Costa, V. & Mazzola, A. Contribution of planktonic and benthic food sources to the diet of the reef-forming vermetid gastropod Dendropoma petraeum in the western Mediterranean. *Estuarine, Coastal and Shelf Science* **96**, 262-267 (2012).

611 Furfaro, G., Trainito, E., De Lorenzi, F., Fantin, M. & Doneddu, M. Tritonia nilsodhneri Marcus Ev., 1983 (Gastropoda, Heterobranchia, Tritoniidae): first records for the Adriatic Sea and new data on ecology and distribution of Mediterranean populations. *Acta Adriat* **58**, 261-270 (2017).

612 Chatzinikolaou, E., Grigoriou, P., Martini, E. & Sterioti, A. Impact of ocean acidification and warming on the feeding behaviour of two gastropod species. *Mediterranean Marine Science* **20**, 669-679 (2019).

613 Mazzella, L. & Russo, G. F. Grazing effect of two Gibbula species (Mollusca, Archaeogastropoda) on the epiphytic community of Posidonia oceanica leaves. *Aquatic Botany* **35**, 357-373 (1989).

614 Cartes, J. Diets of, and trophic resources exploited by, bathyal penaeoidean shrimps from the western Mediterranean. *Marine and Freshwater Research* **46**, 889-996 (1995).

615 Kapiris, K. Feeding ecology of Parapenaeus longirostris (Lucas, 1846)(Decapoda: Penaeidae) from the Ionian Sea (Central and Eastern Mediterranean Sea). *Scientia marina* **68**, 247-256 (2004).

616 Nouar, A., Kennouche, H. & Cartes, N. A. J. E. Temporal changes in the diet of deep-water Penaeoidean shrimp(Parapenaeus longirostris and Aristeus antennatus) off Algeria(southwestern Mediterranean). *Scientia Marina(Barcelona)* **75**, 279-288 (2011).

617 Sobrino, I., Silva, C., Sbrana, M. & Kapiris, K. A review of the biology and fisheries of the deep water rose shrimp, Parapenaeus longirostris, in European Atlantic and Mediterranean waters (Decapoda, Dendrobranchiata, Penaeidae). *Crustaceana*, 1153-1184 (2005).

618 Sardà, F. & Cartes, J. Morphological features and ecological aspects of early juvenile specimens of the aristeid shrimp Aristeus antennatus (Risso, 1816). *Marine and Freshwater Research* **48**, 73-77 (1997).

619 Cartes, J. & Sardà, F. Feeding ecology of the deep-water aristeid crustacean Aristeus antennatus. *Marine ecology progress series. Oldendorf* **54**, 229-238 (1989).

620 Cartes, J. E., Papiol, V. & Guijarro, B. The feeding and diet of the deep-sea shrimp Aristeus antennatus off the Balearic Islands (Western Mediterranean): Influence of environmental factors and relationship with the biological cycle. *Progress in Oceanography* **79**, 37-54 (2008).

621 Kapiris, K. & Thessalou-Legaki, M. Feeding ecology of the deep-water blue–red shrimp Aristeus antennatus (Decapoda: Aristeidae) in the Greek Ionian Sea (E. Mediterranean). *Journal of Sea Research* **65**, 151-160 (2011).

622 Chartosia, N. *et al.* Diet comparison of the bathyal shrimps, Aristeus antennatus (Risso, 1816) and Aristaeomorpha foliacea (Risso, 1827)(Decapoda, Aristeidae) in the eastern Mediterranean. *Crustaceana*, 273-284 (2005).

623 Bello, G. & Pipitone, C. Predation on cephalopods by the giant red shrimp Aristaeomorpha foliacea. *Journal of the Marine Biological Association of the United Kingdom* **82**, 213-218 (2002).

624 Kapiris, K., Thessalou‐Legaki, M., Petrakis, G. & Conides, A. Ontogenetic shifts and temporal changes in the trophic patterns of the deep‐sea red shrimp, Aristaeomorpha foliacea (Decapods: Aristeidae), in the Eastern Ionian Sea (Eastern Mediterranean). *Marine Ecology* **31**, 341-354 (2010).

625 Cartes, J. E. *et al.* Spatial variability in the trophic ecology and biology of the deep-sea shrimp Aristaeomorpha foliacea in the Mediterranean Sea. *Deep Sea Research Part I: Oceanographic Research Papers* **87**, 1-13 (2014).

626 Papaconstantinou, C. & Kapiris, K. The biology of the giant red shrimp (Aristaeomorpha foliacea) at an unexploited fishing ground in the Greek Ionian Sea. *Fisheries Research* **62**, 37-51 (2003).

627 Bayhan, K., Cartes, J. E. & Fanelli, E. Biological condition and trophic ecology of the deep-water shrimp Aristaeomorpha foliacea in the Levantine Sea (SW Turkey). *Mediterranean Marine Science* **16**, 103-116 (2015).

628 Politou, C.-Y., Kapiris, K., Maiorano, P., Capezzuto, F. & Dokos, J. Deep-sea mediterranean biology: the case of Aristaeomorpha foliacea (Risso, 1827)(Crustacea: Decapoda: Aristeidae). *Scientia Marina* **68**, 129-139 (2004).

629 Karani, I., Kitsos, M.-S., Chartosia, N. & Koukouras, A. Diet composition of the penaeid shrimp, Melicertus kerathurus (Forskål, 1775)(Decapoda, Penaeidae) in the Aegean Sea. *Crustaceana*, 385-396 (2005).

630 Cartes, J. E. & Maynou, F. Food consumption by bathyal decapod crustacean assemblages in the western Mediterranean: predatory impact of megafauna and the food consumption-food supply balance in a deep-water food web. *Marine Ecology Progress Series* **171**, 233-246 (1998).

631 Cartes, J. E. Feeding strategies and partition of food resources in deep-water decapod crustaceans (400–2300 m). *Journal of the Marine Biological Association of the United Kingdom* **78**, 509-524 (1998).

632 Cartes, J. *Análisis de las comunidades y estructura trófica de los crustáceos decápodos batiales del Mar Catalán. Universidad Politécnica de Catalunya*, Tesis doctoral. 627 pp, (1991).

633 Cartes, J. Diets of deep-water pandalid shrimps on the Western Mediterranean slope. *Marine Ecology Progress Series* **96**, 49-61 (1993).

634 Fanelli, E. & Cartes, J. E. Feeding habits of pandalid shrimps in the Alboran Sea (SW Mediterranean): influence of biological and environmental factors. *Marine Ecology Progress Series* **280**, 227-238 (2004).

635 Guerao, G. Feeding habits of the prawns Procesa edulis and Palaemon adspersus (Crustacea, Decapoda, Caridea) in Alfacs Bay, Ebro Delta (NW Mediterranean). (1993).

636 Atkinson, R., Gramitto, M. & Froglia, C. Aspects of the biology of the burrowing shrimp Alpheus glaber (Olivi)(Decapoda: Caridea: Alpheidae) from the central Adriatic. *Ophelia* **57**, 27-42 (2003).

637 Kitsos, M.-S., Tzomos, T., Anagnostopoulou, L. & Koukouras, A. Diet composition of the pandalid shrimp, Plesionika narval (Fabricius, 1787)(Decapoda, Pandalidae) in the Aegean Sea. *Crustaceana*, 23-33 (2008).

638 Kalogirou, S. *et al.* Spatial and temporal distribution of narwal shrimp Plesionika narval (Decapoda, Pandalidae) in the Aegean Sea (eastern Mediterranean Sea). *Regional Studies in Marine Science* **16**, 240-248 (2017).

639 Bordbar, L. *et al.* Diet composition and temporal changes in the trophic patterns of Plesionika narval (Crustacea-Decapoda) in the Aegean Sea (Eastern Mediterranean Sea). *Regional Studies in Marine Science* **30**, 100739 (2019).

640 Cristo, M. & Cartes, J. E. A comparative study of the feeding ecology of Nephrops norvegicus (L.),(Decapoda: Nephropidae) in the bathyal Mediterranean and the adjacent Atlantic. *Scientia Marina* **62**, 81-90 (1998).

641 Johnson, M. P., Lordan, C. & Power, A. M. Habitat and ecology of Nephrops norvegicus. *Advances in marine biology* **64**, 27-63 (2013).

642 Sardà, F. Nephrops norvegicus (L.): Comparative biology and fishery in the Mediterranean Sea. Introduction, conclusions and recommendations. (1998).

643 Bernárdez, C., Freire, J. & González-Gurriarán, E. Feeding of the spider crab Maja squinado in rocky subtidal areas of the Ría de Arousa (north-west Spain). *Journal of the Marine Biological Association of the United Kingdom* **80**, 95-102 (2000).

644 Goñi, R., Quetglas, A. & Reñones, O. Diet of the spiny lobster Palinurus elephas (Decapoda: Palinuridea) from the Columbretes Islands Marine Reserve (north-western Mediterranean). *Journal of the Marine Biological Association of the United Kingdom* **81**, 347-348 (2001).

645 Froglia, C. Field observations on diel rhythms in catchability and feeding of Squilla mantis (L.)(Crustacea, Stomatopoda) in the Adriatic Sea. *Biology of stomatopods* (1989).

646 Goñi, R. & Latrouite, D. Review of the biology, ecology and fisheries of Palinurus spp. species of European waters: Palinurus elephas (Fabricius, 1787) and Palinurus mauritanicus (Gruvel, 1911). *Cahiers de Biologie Marine* **46**, 127-142 (2005).

647 Haddadi, M. L. & Hemida, F. Growth and assessment parameters of Calappa granulata (Crustacea; Decapoda; Brachyura) in the eastern Algerian coast (Southern Mediterranean Sea).

648 Cartes, J. Diets of deep-sea brachyuran crabs in the Western Mediterranean Sea. *Marine Biology* **117**, 449-457 (1993).

649 Huguet, C., Maynou, F. & Abelló, P. Small-scale distribution characteristics of Munida spp. populations (Decapoda: Anomura) off the Catalan coasts (western Mediterranean). *Journal of Sea Research* **53**, 283-296 (2005).

650 Chartosia, N., Kitsos, M.-S., Tzomos, T., Mavromati, E. & Koukouras, A. Diet composition of five species of crabs (Decapoda, Brachyura) that show a gradual transition from marine to terrestrial life. *Crustaceana*, 1181-1197 (2010).

651 Abelló, P. Feeding habits of Macropipus tuberculatus (Brachyura, Portunidae) off the Catalan coast (NW Mediterranean). *Miscellània Zoològica*, 45-50 (1989).

652 Cartes, J. E. *et al.* Feeding guilds of western Mediterranean demersal fish and crustaceans: an analysis based in a spring survey. *Scientia Marina* **66**, 209-220 (2002).

653 Cebrian, E., Ballesteros, E., Linares, C. & Tomas, F. Do native herbivores provide resistance to Mediterranean marine bioinvasions? A seaweed example. *Biological Invasions* **13**, 1397-1408 (2011).

654 Sierra, A., García, L. & Lloris, D. Trofismo y competencia alimentaria en asteroideos de la Bahía de Almería. (1978).

655 Rodríguez, A. Alimentación y comportamiento alimentario de los equinodermos. *Lagena* **29**, 23-31 (1972).

656 Coulon, P. & Jangoux, M. Feeding rate and sediment reworking by the holothuroid Holothuria tubulosa (Echinodermata) in a Mediterranean seagrass bed off Ischia Island, Italy. *Marine Ecology Progress Series*, 201-204 (1993).

657 Murillo-Navarro, R. & Jiménez-Guirado, D. Relationships between algal food and gut and gonad conditions in the Mediterranean sea urchin Paracentrotus lividus (Lam.). *Mediterranean Marine Science* **13**, 227-238 (2012).

658 Ribes, M., Coma, R. & Gili, J.-M. Seasonal variation of in situ feeding rates by the temperate ascidian Halocynthia papillosa. *Marine Ecology Progress Series* **175**, 201-213 (1998).

659 Coppari, M., Gori, A. & Rossi, S. Size, spatial, and bathymetrical distribution of the ascidian Halocynthia papillosa in Mediterranean coastal bottoms: benthic–pelagic coupling implications. *Marine biology* **161**, 2079-2095 (2014).

660 Coppari, M. *et al.* The role of Mediterranean sponges in benthic–pelagic coupling processes: Aplysina aerophoba and Axinella polypoides case studies. *Journal of Experimental Marine Biology and Ecology* **477**, 57-68 (2016).

661 Lucas, C. H. *et al.* Gelatinous zooplankton biomass in the global oceans: geographic variation and environmental drivers. *Global Ecology and Biogeography* **23**, 701-714 (2014).

662 Palomares, M. & Pauly, D. in *Jellyfish Blooms: Causes, Consequences, and Recent Advances* 11-21 (Springer, 2009).

663 Sabatés, A. *et al.* in *Jellyfish Blooms: New Problems and Solutions* 153-165 (Springer, 2010).

664 Pascual, M. Spatio-temporal processes explaining salp aggregations and their role in the Catalan Sea, Northwestern Mediterranean Sea. (2016).

665 Tilves, U. *et al.* Trophic interactions of the jellyfish Pelagia noctiluca in the NW Mediterranean: evidence from stable isotope signatures and fatty acid composition. *Marine Ecology Progress Series* **591**, 101-116 (2018).

666 Dadon‐Pilosof, A., Lombard, F., Genin, A., Sutherland, K. R. & Yahel, G. Prey taxonomy rather than size determines salp diets. *Limnology and Oceanography* **64**, 1996-2010 (2019).

667 Marques, R., Bonnet, D., Carré, C., Roques, C. & Darnaude, A. M. Trophic ecology of a blooming jellyfish (Aurelia coerulea) in a Mediterranean coastal lagoon. *Limnology and Oceanography* **66**, 141-157 (2021).

668 Tilves, U. *et al.* Natural diet and predation impacts of Pelagia noctiluca on fish eggs and larvae in the NW Mediterranean. *Journal of Plankton Research* **38**, 1243-1254 (2016).

669 Pitt, K., Connolly, R. & Meziane, T. Stable isotope and fatty acid tracers in energy and nutrient studies of jellyfish: a review. *Jellyfish Blooms: Causes, Consequences, and Recent Advances*, 119-132 (2008).

670 Turan, C. *et al.* in *First National Workshop on Jellyfish and Other Gelatinous Species in Turkish Marine Waters.* (eds C. Turan & B. Öztürk) 90 (Turkish Marine Research Foundation

).

671 Palmieri, M. G., Barausse, A., Luisetti, T. & Turner, K. Jellyfish blooms in the Northern Adriatic Sea: Fishermen's perceptions and economic impacts on fisheries. *Fisheries Research* **155**, 51-58 (2014).

672 Coma, R., Ribes, M., Zabala, M. & Gili, J.-M. Growth in a modular colonial marine invertebrate. *Estuarine, Coastal and Shelf Science* **47**, 459-470 (1998).

673 Grinyó, J. *et al.* Diversity, distribution and population size structure of deep Mediterranean gorgonian assemblages (Menorca Channel, Western Mediterranean Sea). *Progress in Oceanography* **145**, 42-56 (2016).

674 Linares, C., Coma, R., Garrabou, J., Díaz, D. & Zabala, M. Size distribution, density and disturbance in two Mediterranean gorgonians: Paramuricea clavata and Eunicella singularis. *Journal of Applied Ecology* **45**, 688-699 (2008).

675 Peirano, A., Morri, C., Bianchi, C. N. & Rodolfo-Metalpa, R. Biomass, carbonate standing stock and production of the mediterranean coralCladocora caespitosa (L.). *Facies* **44**, 75-80 (2001).

676 Angiolillo, M. *et al.* Distribution and population structure of deep‐dwelling red coral in the Northwest Mediterranean. *Marine Ecology* **37**, 294-310 (2016).

677 Bavestrello, G., Bo, M., Canese, S., Sandulli, R. & Cattaneo-Vietti, R. The red coral populations of the gulfs of Naples and Salerno: human impact and deep mass mortalities. *Italian Journal of Zoology* **81**, 552-563 (2014).

678 Bavestrello, G., Bo, M., Bertolino, M., Betti, F. & Cattaneo‐Vietti, R. Long‐term comparison of structure and dynamics of the red coral metapopulation of the Portofino Promontory (Ligurian Sea): a case‐study for a Marine Protected Area in the Mediterranean Sea. *Marine ecology* **36**, 1354-1363 (2015).

679 Cau, A. *et al.* Habitat constraints and self-thinning shape Mediterranean red coral deep population structure: implications for conservation practice. *Scientific reports* **6**, 23322 (2016).

680 Garrabou, J. *et al.* Re-shifting the ecological baseline for the overexploited Mediterranean red coral. *Scientific reports* **7**, 42404 (2017).

681 Bo, M. *et al.* Deep coral oases in the South Tyrrhenian Sea. *PloS one* **7**, e49870 (2012).

682 Linares, C. *et al.* Marine Protected Areas and the conservation of long-lived marine invertebrates: the Mediterranean red coral. *Marine Ecology Progress Series* **402**, 69-79 (2010).

683 Sini, M., Garrabou, J., Trygonis, V. & Koutsoubas, D. Coralligenous formations dominated by Eunicella cavolini (Koch, 1887) in the NE Mediterranean: biodiversity and structure. *Mediterranean Marine Science* **20**, 174-188 (2019).

684 Sini, M., Kipson, S., Linares, C., Koutsoubas, D. & Garrabou, J. The yellow gorgonian Eunicella cavolini: demography and disturbance levels across the Mediterranean Sea. *PloS one* **10**, e0126253 (2015).

685 Mistri, M. & Ceccherelli, V. U. Growth and secondary production of the Mediterranean gorgonian Paramuricea clavata. *Marine Ecology-Progress Series* **103**, 291-291 (1994).

686 Weinbauer, M. G. & Velimirov, B. Biomass and secondary production of the temperate gorgonian coral Eunicella cavolini (Coelenterata: Octocorallia). *Marine Ecology Progress Series* **121**, 211-216 (1995).

687 Martin, C. *et al.* Coralligenous and maërl habitats: predictive modelling to identify their spatial distributions across the Mediterranean Sea. *Scientific Reports* (2014).

688 Agnesi, S. *et al.* in *Proceedings of the 1st Mediterranean symposium on the conservation of the coralligenous and other calcareous bio-concretions.* (UNEP-MAP RAC/SPA (Tabarka, 15-16 January 2009). Tunis, RAC/SPA publication).

689 Molina, A. C. *et al.* Combining literature review, acoustic mapping and in situ observations: an overview of coralligenous assemblages in Liguria (NW Mediterranean Sea). *Sci. Mar* **80**, 7-16 (2016).

690 Bramanti, L. *et al.* Demography of Animal Forests: The Example of Mediterranean Gorgonians. *Marine Animal Forests: The Ecology of Benthic Biodiversity Hotspots*, 1-20 (2015).

691 Tsounis, G. *Demography, Reproductive Biology and Trophic Ecology of Red Coral (Corallium rubrum L.) at the Costa Brava (NW Mediterranean): Ecological Data as a Tool for Management. 104 S.{*[*http://elib*](http://elib)*. suub. uni-bremen. de/publications/dissertations/E-Diss1246_TsounisG. pdf}*, University Bremen, (2005).

692 Cocito, S. *et al.* Nutrient acquisition in four Mediterranean gorgonian species. *Marine Ecology Progress Series* **473**, 179-188 (2013).

693 Ribes, M., Coma, R. & Gili, J.-M. Heterogeneous feeding in benthic suspension feeders: the natural diet and grazing rate of the temperate gorgonian Paramuricea clavata (Cnidaria: Octocorallia) over a year cycle. *Marine Ecology Progress Series* **183**, 125-137 (1999).

694 Tsounis, G. *et al.* Diet and seasonal prey capture rates in the Mediterranean red coral (Corallium rubrum L.). *Marine Biology* **149**, 313-325 (2006).

695 Rossi, S. *Environmental factors affecting the trophic ecology of benthic suspension feeders*, University of Barcelona, (2001).

696 Picciano, M. & Ferrier-Pagès, C. Ingestion of pico-and nanoplankton by the Mediterranean red coral Corallium rubrum. *Marine Biology* **150**, 773-782 (2007).

697 Gori, A. *et al.* Effects of food availability on the sexual reproduction and biochemical composition of the Mediterranean gorgonian Paramuricea clavata. *Journal of experimental marine biology and ecology* **444**, 38-45 (2013).

698 Enrichetti, F. *et al.* Artisanal fishing impact on deep coralligenous animal forests: a Mediterranean case study of marine vulnerability. *Ocean & Coastal Management* **177**, 112-126 (2019).

699 Macias, D., Huertas, I. E., Garcia-Gorriz, E. & Stips, A. Non-Redfieldian dynamics driven by phytoplankton phosphate frugality explain nutrient and chlorophyll patterns in model simulations for the Mediterranean Sea. *Progress in oceanography* **173**, 37-50 (2019).

700 Holmer, M., Duarte, C. M., Boschker, H. & Barrón, C. Carbon cycling and bacterial carbon sources in pristine and impacted Mediterranean seagrass sediments. *Aquatic Microbial Ecology* **36**, 227-237 (2004).

701 Buia, M. & Mazzella, L. Diversity in seagrass ecosystems: biological descriptors at different temporal scale. *Biologia marina mediterranea* **7**, 203-206 (2000).

702 Buia, M., Gambi, M. & Zupo, V. Structure and functioning of Mediterranean seagrass ecosystems: an overview. *Biologia Marina Mediterranea* **7**, 167-190 (2000).

703 Giannoulaki M. *et al.* *Mediterranean Sensitive Habitats DG MARE Specific Contract SI2.600741, Final Report, 557 p.*, (2013).

704 Marbá, N. *et al.* Growth and population dynamics of Posidonia oceanica on the Spanish Mediterranean coast: elucidating seagrass decline. *Marine Ecology Progress Series* **137**, 203-213 (1996).

705 Lepoint, G., Havelange, S., Gobert, S. & Bouquegneau, J.-M. Fauna vs flora contribution to the leaf epiphytes biomass in a Posidonia oceanica seagrass bed (Revellata Bay, Corsica). *Hydrobiologia* **394**, 63-67 (1999).

706 Gambi, M. C. *et al.* Proceedings of the Mediterranean Seagrass Workshop 2006. *Biologia marina mediterranea* **13**, 293 pp. (2006).

707 Bay, D. A field study of the growth dynamics and productivity of *Posidonia oceanica* (L.) delile in Calvi Bay, Corsica. *Aquatic Botany* **20**, 43-64 (1984).

708 Guidetti, P. Leaf primary production in Posidonia oceanica: two reconstructive aging techniques give similar results. *Aquatic Botany* **68**, 337-343 (2000).

709 Duarte, C. M. *et al.* Root production and belowground seagrass biomass. *Marine Ecology Progress Series* **171**, 97-108 (1998).

710 Pergent, G., Romero, J., Pergent-Martini, C., Mateo, M.-A. & Boudouresque, C.-F. Primary production, stocks and fluxes in the Mediterranean seagrass Posidonia oceanica. *Marine Ecology-Progress Series* **106**, 139-139 (1994).

711 Sghaier, Y. R., Zakhama-Sraieb, R. & Charfi-Cheikhrouha, F. Patterns of shallow seagrass (*Posidonia oceanica*) growth and flowering along the Tunisian coast. *Aquatic Botany* **104**, 185-192 (2013).

712 Terrados, J. & Pons, F. J. M. Epiphyte load on the seagrass Posidonia oceanica (L.) Delile does not indicate anthropogenic nutrient loading in Cabrera Archipelago National Park (Balearic Islands, Western Mediterranean). *Scientia Marina* **72**, 503-510 (2008).

713 Mediterranean Seagrass Association. in *Mediterranean Seagrass Workshop (MSW12).* 43 pp.

714 Ott, J. A. Growth and production in Posidonia oceanica (L.) Delile. *Marine Ecology* **1**, 47-64 (1980).

715 Mustapha, K. B., Afli, A., Hattour, A. & El Abed, A. Sessile megabenthic species from Tunisian littoral sites. *MedSudMed Technical Documents* **2**, 1-16 (2004).

716 Pedersen, M. F., Duarte, C. M. & Cebrián, J. Rates of changes in organic matter and nutrient stocks during seagrass Cymodoceanodosa colonization and stand development. *Marine Ecology Progress Series* **159**, 29-36 (1997).

717 Barrón, C., Marbé, N., Terrados, J., Kennedy, H. & Duarte, C. M. Community metabolism and carbon budget along a gradient of seagrass (Cymodocea nodosa) colonization. *Limnology and Oceanography* **49**, 1642-1651 (2004).

718 Cebrián, J., Duarte, C., Marbà, N. & Enriquez, S. Magnitude and fate of the production of four cooccurring western Mediterranean seagrass species. *Marine Ecology Progress Series* **155**, 29-44 (1997).

719 Cancemi, G., Buia, M. C. & Mazzella, L. Structure and growth dynamics of Cymodocea nodosa meadows. *Scientia Marina* **66**, 365-373 (2002).

720 Pérez, M. & Romero, J. Growth dynamics, production, and nutrient status of the seagrass Cymodocea nodosa in a Mediterranean semi‐estuarine environment. *Marine Ecology* **15**, 51-64 (1994).

721 Marbà, N. & Duarte, C. M. Growth and sediment space occupation by seagrass Cymodocea nodosa roots. *Marine Ecology-Progress Series* **224**, 291-298 (2001).

722 Zavodnik, N., Travizi, A. & De Rosa, S. Seasonal variations in the rate of photosynthetic activity and chemical composition of the seagrass Cymodocea nodosa (Ucr.) Asch. *Scientia Marina* **62**, 301-309 (1998).

723 Githaiga, M. N., Gilpin, L., Kairo, J. G. & Huxham, M. Biomass and productivity of seagrasses in Africa. *Botanica Marina* **59**, 173-186 (2016).

724 Amoutzopoulou-Schina, H. & Haritonidis, S. Distribution and phenology of the marine phanerogam Posidonia oceanica in the Pagassitikos Gulf, Greece. *Journal of Biological Research* **4**, 203-211 (2005).

725 Badalamenti, F., Di Carlo, G., D’Anna, G., Gristina, M. & Toccaceli, M. Effects of dredging activities on population dynamics of Posidonia oceanica (L.) Delile in the Mediterranean sea: the case study of Capo Feto (SW Sicily, Italy). *Hydrobiologia* **555**, 253-261 (2006).

726 Beqiraj, S. *et al.* Benthic macrofauna of Posidonia oceanica meadows in the Albanian coast. *Natura montenegrina* **7**, 55-69 (2008).

727 Borg, J., Rowden, A., Attrill, M., Schembri, P. & Jones, M. Occurrence and distribution of different bed types of seagrass Posidonia oceanica around the Maltese Islands. *Mediterranean Marine Science* **10**, 45-62 (2009).

728 Costantino, G. *et al.* Distribution and bio-ecological features of Posidonia oceanica meadows along the coasts of the southern Adriatic and northern Ionian Seas. *Chemistry and Ecology* **26**, 91-104 (2010).

729 Drew, E. A. Factors affecting photosynthesis and its seasonal variation in the seagrasses Cymodocea nodosa (Ucria) Aschers, and Posidonia oceanica (L.) Delile in the Mediterranean. *Journal of Experimental Marine Biology and Ecology* **31**, 173-194 (1978).

730 Dural, B., Aysel, V., Demir, N. & Erduğan, H. The status of sensitive ecosystems along the Aegean coast of Turkey: Posidonia oceanica (L.) Delile meadows. *Journal of the Black Sea/Mediterranean Environment* **18** (2012).

731 Gerakaris, V., Panayotidis, P., Vizzini, S., Nicolaidou, A. & Economou-Amilli, A. Effectiveness of Posidonia oceanica biotic indices for assessing the ecological status of coastal waters in Saronikos Gulf (Aegean Sea, Eastern Mediterranean). *Mediterranean Marine Science* **18**, 161-178 (2017).

732 Giardina, F. & De Rubeis, P. Analisi della prateria a Posidonia oceanica (L.) Delile (Najadales, Potamogetonaceae) dell’isola di Lampedusa (AMP “Isole Pelagie”, Canale di Sicilia). *Bollettino Accademia Gioenia di Scienze Naturali di Catania* **45**, 651-664 (2012).

733 Lardi, P., Gerakaris, V., Panayotidis, P., Danielidis, D. & Economou-Amilli, A. Study of the morphological and structural features of the seagrass Cymodocea nodosa (Ucria) Ascherson in an embayment of the Aegean Sea (Saronikos Gulf, Greece). (2016).

734 Holmer, M. & Frederiksen, M. S. Stimulation of sulfate reduction rates in Mediterranean fish farm sediments inhabited by the seagrass Posidonia oceanica. *Biogeochemistry* **85**, 169-184 (2007).

735 Mabrouk, L., Ben Brahim, M., Hamza, A., Mahfoudhi, M. & Bradai, M. N. A comparison of abundance and diversity of epiphytic microalgal assemblages on the leaves of the seagrasses Posidonia oceanica (L.) and Cymodocea nodosa (Ucria) Asch in Eastern Tunisia. *Journal of Marine Biology* **2014** (2014).

736 Malea, P. & Zikidou, C. Temporal variation in biomass partitioning of the seagrass Cymodocea nodosa at the Gulf of Thessaloniki, Greece. *Journal of Biological Research* **15**, 75 (2011).

737 Mezali, K., Zupo, V., Francour, P. & Societa Italiana di Biologia Marina, L. Vol. 13 (Erredi Grafiche Editoriali, Genova(Italy), 2006).

738 Brodersen, M. M. *et al.* Cumulative impacts from multiple human activities on seagrass meadows in eastern Mediterranean waters: the case of Saronikos Gulf (Aegean Sea, Greece). *Environmental Science and Pollution Research* **25**, 26809-26822 (2018).

739 Peduzzi, P. & Herndl, G. J. Decomposition and significance of seagrass leaf litter(Cymodocea nodosa) for the microbial food web in coastal waters(Gulf of Trieste, northern Adriatic Sea). *Marine ecology progress series. Oldendorf* **71**, 163-174 (1991).

740 Piazzi, L., Acunto, S. & Cinelli, F. Mapping of Posidonia oceanica beds around Elba Island (western Mediterranean) with integration of direct and indirect methods. *Oceanologica acta* **23**, 339-346 (2000).

741 Tsirika, A., Skoufas, G. & Haritonidis, S. Seasonal and bathymetric variations of epiphytic macroflora on Posidonia oceanica (L.) Delile leaves in the National Marine Park of Zakynthos (Greece). *Marine Ecology* **28**, 146-153 (2007).

742 La Loggia, G. *et al.* Influence of hydrodynamic conditions on the production and fate of Posidonia oceanica in a semi-enclosed shallow basin (Stagnone di Marsala, Western Sicily). *Chemistry and Ecology* **20**, 183-201 (2004).

743 Apostolaki, E. T., Holmer, M., Marbà, N. & Karakassis, I. Reduced carbon sequestration in a Mediterranean seagrass (Posidonia oceanica) ecosystem impacted by fish farming. *Aquaculture Environment Interactions* **2**, 49-59 (2011).

744 Lepoint, G., Defawe, O., Gobert, S., Dauby, P. & Bouquegneau, J.-M. Experimental evidence for N recycling in the leaves of the seagrass Posidonia oceanica. *Journal of Sea Research* **48**, 173-179 (2002).

745 Wittmann, K. J. Temporal and morphological variations of growth in a natural stand of Posidonia oceanica (L.) Delile. *Marine ecology* **5**, 301-316 (1984).

746 Duarte, C. M. Allometric scaling of seagrass form and productivity. *Marine ecology progress series. Oldendorf* **77**, 289-300 (1991).

747 Capiomont, A., Breugnot, E., den Haan, M. & Meinesz, A. Phenology of a deep-water population of Caulerpa racemosa var. cylindracea in the northwestern Mediterranean Sea. *Botanica marina* **48**, 80-83 (2005).

748 Marbà, N., Duarte, C.M. Spanish document aiming at the identification of Important ecosystem properties and assessment of ecological status and pressures to Mediterranean marine and coastal biodiversity. 56 pp. (Institut Mediterrani d’Estudis Avançats (CSIC-UIB), Esporles (Spain), 2010).

749 Box, A. *et al.* Seasonality of caulerpenyne content in native Caulerpa prolifera and invasive C. taxifolia and C. racemosa var. cylindracea in the western Mediterranean Sea. *Botanica Marina* **53**, 367-375 (2010).

750 Joher Sais, S. Macroalgal-dominated coastal detritic bottoms of the Mediterranean Sea and the Northeastern Atlantic: description, distribution and sampling methodologies. (2016).

751 Linares, C. *et al.* Persistent natural acidification drives major distribution shifts in marine benthic ecosystems. *Proceedings of the Royal Society B: Biological Sciences* **282**, 20150587 (2015).

752 Ballesteros, E. Structure and dynamics of the community of Cystoseira zosteroides (Turner) C. Agardh (Fucales, Phaeophyceae) in the Northwestern Mediterranean. (1990).

753 Sales, M. & Ballesteros, E. Seasonal dynamics and annual production of Cystoseira crinita (Fucales: Ochrophyta)-dominated assemblages from the northwestern Mediterranean. *Scientia marina* **76**, 391-401 (2011).

754 Ballesteros, E. Estructura y dinámica de la comunidad de Cystoseira mediterranea Sauvageau en el Mediterráneo Noroccidental. (1988).

755 Ballesteros, E. Structure and dynamics of the Cystoseira caespitosa Sauvageau (Fucales, Phaeophyceae) community in the North-Western Mediterranean. (1990).

756 Sala, E. *et al.* The structure of Mediterranean rocky reef ecosystems across environmental and human gradients, and conservation implications. *PloS one* **7**, e32742 (2012).

757 Sala, E. & Boudouresque, C. F. The role of fishes in the organization of a Mediterranean sublittoral community.: I: Algal communities. *Journal of Experimental Marine Biology and Ecology* **212**, 25-44 (1997).

**Modelling the Mediterranean Sea ecosystem at high spatial resolution to inform the ecosystem-based management in the region**

**Chiara Piroddi, Marta Coll, Diego Macias Moy, Jeroen Steenbeek, Elisa Garcia-Gorriz, Alessandro Mannini, Daniel Vilas, Villy Christensen**

**S4.**

**
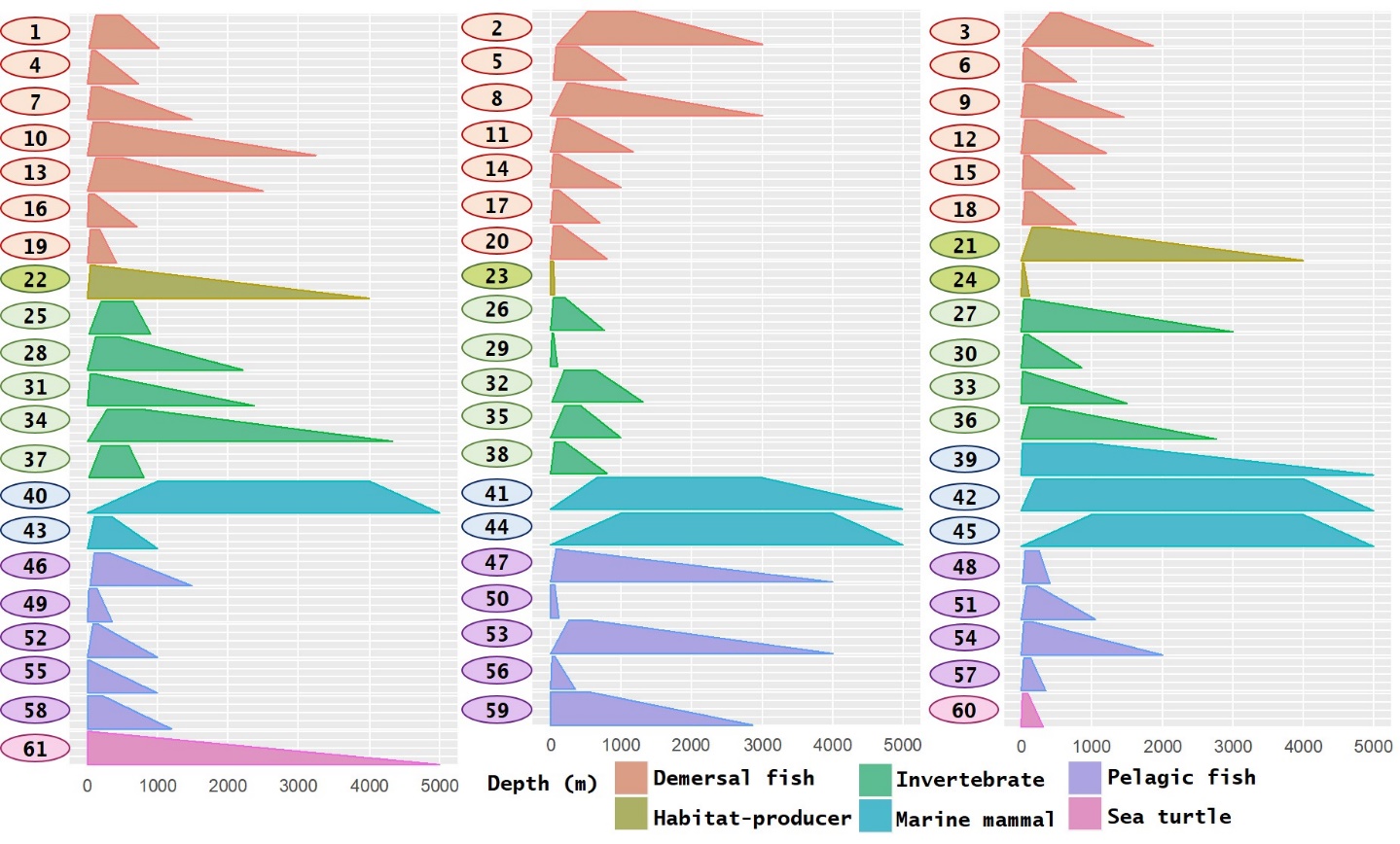
Fig S2.** Depth response curves used for selected species/functional groups of the Mediterranean Sea ecosystem. **Demersal fish:** 1: Anglerfish; 2: Bathydemersal fish; 3: Blackmouth catshark; 4: Other commercial small demersal fish; 5: European hake (Adult); 6: European hake (Recruit); 7: Flatfish; 8: Gadidae; 9: Non-commercial medium demersal fish; 10: Non-commercial small demersal fish; 11: Other commercial large demersal fish; 12: Other commercial medium demersal fish; 13: Other small demersal shark; 14: Ray&Skate; 15: Red mullet (Adult); 16: Red mullet (Recruit); 17: Sparidae; 18: Small-spotted catshark; 19: Surmullet; 20: Torpedo; **Habitat-producer:** 21: Coral&Gorgonian; 22: Sessile benthos; 23: Seagrass; 24: Seaweed; **Invertebrates:** 25: Blue and red shrimp; 26: Benthic cephalopod; 27: Bivalve; 28: Benthopelagic cephalopod; 29: Commercial prawn; ; 30: Deep-water rose shrimp; 31: Gastropod; 32: Giant red shrimp; 33: Mobile benthos; 34: Mesopelagic cephalopod; 35: Non-commercial shrimp/prawn; 36: Non-commercial decapod; 37: Norway lobster; 38: Other commercial decapod; **Marine mammal:** 39: Bottlenose dolphin; 40: Common dolphin; 41: Deep Sea cetacean; 42: Fin whale; 43: Monk seal; 44: Striped dolphin; 45: Sperm whale; **Pelagic fish:** 46: Benthopelagic fish; 47: Bluefin tuna; 48: European anchovy (Adult); 49: European anchovy (Recruit); 50: European pilchard (Adult) and European pilchard (Recruit); 51: Horse mackerel; 52: Mackerel; 53: Mesopelagic fish; 54: Non-commercial large pelagic fish; 55: Other commercial large pelagic fish; 56: Other medium pelagic fish; 57: Other small pelagic fish; 58: Pelagic shark; 59: Swordfish; **Sea turtle:** 60: Green turtle; 61: Loggerhead turtle.

**
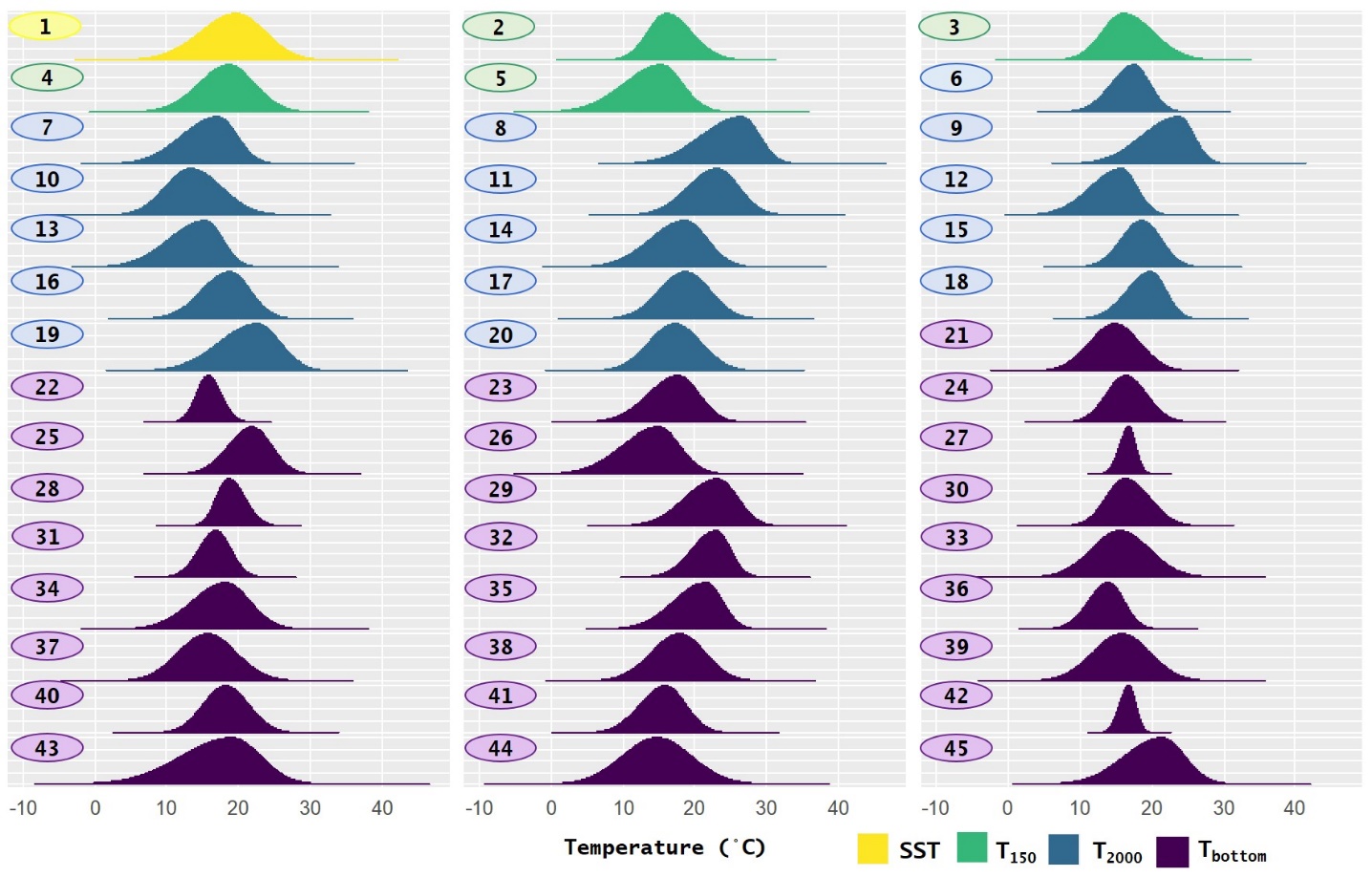
**

**Fig S3.** Temperature response curves used for selected species/functional groups of the Mediterranean Sea ecosystem. **SST:** 1: European anchovy (Adult) and European anchovy (Recruit); **T_150_**: 2: Horse mackerel; 3: Mackerel; 4: Other medium pelagic fish; 5: European pilchard (Adult) and European pilchard (Recruit); **T_2000_:** 6: Benthopelagic fish; 7: Benthopelagic cephalopod; 8: Loggerhead turtle; 9: Other commercial large pelagic fish; 10: Gadidae; 11: Green turtle; 12: European hake (Adult); 13: Mesopelagic fish; 14: Mesopelagic cephalopod; 15: Non-commercial medium demersal fish; 16: Other commercial medium demersal fish; 17: Pelagic shark; 18: Sparidae; 19: Swordfish; 20: Bluefin tuna; **T_bottom_:** 21: Anglerfish; 22: Bathydemersal fish; 23: Benthic cephalopod; 24: Bivalve; 25: Blue and red shrimp; 26: Blackmouth catshark; 27: Coral&Gorgonian; 28: Other commercial small demersal fish; 29: Deep-water rose shrimp; 30: Flatfish; 31: Gastropod; 32: Giant red shrimp; 33: Non-commercial decapod; 34: Non-commercial small demersal fish; 35: Non-commercial shrimp/prawn; 36: Norway lobster; 37: Other commercial decapod; 38: Other commercial large demersal fish; 39: Other small demersal shark; 40: Ray&Skate; 41: Red mullet (Adult) and Red mullet (Recruit); 42: Sessile benthos; 43: Small-spotted catshark; 44: Surmullet; 45: Torpedo.


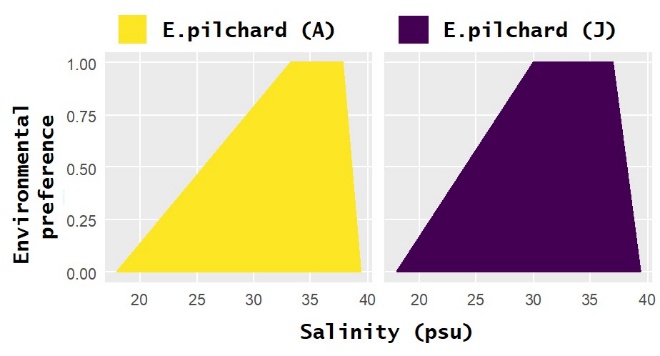


**Figure S4:** Salinity response curves used for European pilchard (Adult) and European pilchard (Recruit).

**Other Ecospace input parameters**

In Ecospace, the parameters that regulate spatial distribution of functional groups are: Base dispersal rate, Relative dispersal in bad habitat, and Relative vulnerability to predation in bad habitat (Table S4). Base Dispersal rate corresponds to swimming speed (km/year) and can be set between 0 and infinite; the default value is 300 km/year. The Relative dispersal in bad habitat increases dispersal rate in non-favourable habitats to simulate greater active attempt to move elsewhere with better conditions. It can be set from 1 (which inactivates the mechanism) to 10; the default is set at 5. Relative vulnerability to predation in bad habitat is a weight factor for the vulnerability parameter in Ecosim. It regulates the increased vulnerability to predation (or decreased sheltering capacity) in a less-than-optimal habitat. Its default value is 2 (twice more vulnerable in bad habitat), and it can be set between 1 and 100.

**Table S4**

| **#** | **Group name** | **Base dispersal rate** | **Relative dispersal in bad habitat (proportion)** | **Rel. vul to pred. in bad hab.** |
| --- | --- | --- | --- | --- |
| 1 | Bottlenose dolphin | 300 | 5 | 2 |
| 2 | Striped dolphin | 300 | 5 | 2 |
| 3 | Common dolphin | 300 | 5 | 2 |
| 4 | Fin whale | 300 | 5 | 2 |
| 5 | Sperm whale | 300 | 5 | 2 |
| 6 | Deep Sea cetaceans | 300 | 5 | 2 |
| 7 | Monk seal | 300 | 5 | 2 |
| 8 | Pelagic Seabird | 300 | 5 | 2 |
| 9 | Gull&Cormorant | 300 | 5 | 2 |
| 10 | Tern | 200 | 5 | 2 |
| 11 | Loggerhead turtle | 300 | 5 | 2 |
| 12 | Green turtle | 300 | 5 | 2 |
| 13 | Pelagic shark | 300 | 5 | 2 |
| 14 | NC large pelagic | 300 | 5 | 2 |
| 15 | Bluefin tuna | 1000 | 5 | 2 |
| 16 | Swordfish | 300 | 5 | 2 |
| 17 | Other large pelagic | 300 | 5 | 2 |
| 18 | Mackerel | 300 | 5 | 2 |
| 19 | Horse mackerel | 200 | 5 | 10 |
| 20 | Other medium pelagic | 30 | 5 | 2 |
| 21 | E. sardine Adult | 30 | 5 | 2 |
| 22 | E. sardine Recruit | 30 | 5 | 2 |
| 23 | E. anchovy Adult | 30 | 5 | 2 |
| 24 | E. Anchovy Recruit | 30 | 5 | 2 |
| 25 | Other small pelagic | 30 | 5 | 2 |
| 26 | Benthopelagic fish | 30 | 5 | 2 |
| 27 | Mesopelagic fish | 30 | 5 | 2 |
| 28 | Anglerfish | 30 | 5 | 2 |
| 29 | E. Hake Adult | 30 | 5 | 2 |
| 30 | E. Hake Recruit | 30 | 5 | 2 |
| 31 | OC large demersal | 30 | 5 | 2 |
| 32 | Gadidae | 30 | 5 | 2 |
| 33 | Sparidae | 500 | 5 | 2 |
| 34 | OC medium demersal f. | 100 | 5 | 2 |
| 35 | Flatfish | 250 | 5 | 2 |
| 36 | NC medium demersal f. | 30 | 5 | 2 |
| 37 | Red mullet Adult | 80 | 5 | 2 |
| 38 | Red mullet Recruit | 80 | 5 | 2 |
| 39 | Surmullet | 80 | 5 | 2 |
| 40 | OC small demersal f. | 80 | 5 | 2 |
| 41 | NC small demersal f. | 60 | 5 | 10 |
| 42 | Bathydemersal f. | 30 | 5 | 2 |
| 43 | Small-spotted catshark | 30 | 5 | 2 |
| 44 | Blackmouth catshark | 300 | 5 | 10 |
| 45 | Other small demersal shark | 30 | 5 | 2 |
| 46 | Ray&Skate | 30 | 5 | 2 |
| 47 | Torpedo | 30 | 5 | 2 |
| 48 | Benthic cephalopod | 3 | 5 | 2 |
| 49 | Benthopelagic cephalopod | 300 | 5 | 2 |
| 50 | Mesopelagic cephalopod | 300 | 5 | 2 |
| 51 | Bivalve | 3 | 5 | 2 |
| 52 | Gastropod | 3 | 5 | 2 |
| 53 | Deep-water rose shrimp | 3 | 5 | 2 |
| 54 | Blue and red shrimp | 3 | 5 | 2 |
| 55 | Giant red shrimp | 3 | 5 | 2 |
| 56 | Commercial prawn | 3 | 5 | 2 |
| 57 | NC shrimp/prawn | 3 | 5 | 2 |
| 58 | Norway lobster | 3 | 5 | 2 |
| 59 | OC decapod | 3 | 5 | 2 |
| 60 | NC decapod | 3 | 5 | 2 |
| 61 | Mobile benthos | 3 | 5 | 2 |
| 62 | Sessile benthos | 1 | 5 | 2 |
| 63 | Jellyfish&Salp | 300 | 5 | 2 |
| 64 | Coral&Gorgonian | 1 | 5 | 2 |
| 65 | Zooplankton | 300 | 5 | 2 |
| 66 | Seagrass | 1 | 5 | 2 |
| 67 | Seaweed | 1 | 5 | 2 |
| 68 | Small Phytoplankton | 300 | 5 | 2 |
| 69 | Large Phytoplankton | 300 | 5 | 2 |
| 70 | Discards | 3 | 5 | 2 |
| 71 | Detritus | 1 | 5 | 2 |

**Modelling the Mediterranean Sea ecosystem at high spatial resolution to inform the ecosystem-based management in the region**

**Chiara Piroddi, Marta Coll, Diego Macias Moy, Jeroen Steenbeek, Elisa Garcia-Gorriz, Alessandro Mannini, Daniel Vilas, Villy Christensen**

**S5.**

**Table S5. Detailed description of modelled derived indicators with acronyms, definitions and references.**

| **Ecological Indicator** | **Acronym** | **Definition and references** |
| --- | --- | --- |
| **Total biomass** | *TB* | Index calculated as the sum of the biomass of all the modelled functional groups (Fg; unit: t/km^2^) ^1^. |
| **Commercial biomass** | *CB* | Index calculated as the sum of the biomass only for those Fg having a commercial value (unit: t/km^2^) ^2^. |
| **Invertebrates/Fish Biomass** | *I/F* | Sum of the biomass of all invertebrate (commercial and non commercial) Fg / sum of the biomass of all (commercial and non commercial) fish Fg ^3,4^. Explores processes benefiting the invertebrates or fish communities. |
| **Demersal/Pelagic Biomass** | *D/P* | Sum of the biomass of all demersal (commercial and non commercial) fish Fg/ sum of the biomass of all pelagic (commercial and non commercial) fish Fg ^3,4^. Explores processes benefiting the demersal or pelagic compartments of the ecosystem. |
| **Kempton species diversity index** | *Qi* | The slope of the cumulative species abundance curve between the 10^th^ and 90^th^ percentiles. It expresses biomass species diversity and evenness by considering those Fg with trophic levels 3 or higher ^5,6^.  The Kempton index is calculated as follow ^6^:  $Q_{i}= \frac{0.8Fg}{log(\frac{R_{2}}{R_{1}})}$  where Fg is the total number of functional groups, R_1_ and R_2_ are the representative biomass values of the 10^th^ and 90^th^ percentiles in the cumulative abundance distribution. |
| **Shannon entropy index** | *H* | Shannon’s entropy index ^7^ is commonly used in ecological studies as a composite metric representing both species evenness and species richness. Applied to EwE however, where functional groups substitute for individual species and the number of functional groups is generally fixed, the index is useful for describing functional group evenness. Here, evenness represents the distribution of biomass across functional groups, where maximum evenness is achieved when all functional groups have equal biomass.  The Shannon index is calculated as follow: $H=-\sum_{i=1}^{n} p_{i}\ln p_{i}$   where n represents the total number of functional groups and pi represents the proportion of total ecosystem biomass present in the ith functional group. |
| **Mean trophic level of community** | *TLco* | TL of the modelled community spans the whole ecosystem (living groups)^8^ including all functional groups. TLs are calculated as follow ^9^:  ^^  where j is the predator of prey i, DC_ji_ is the fraction of prey i in the diet of each predator j, and TL_i_ is the TL of prey i.  The mean trophic level of community is calculated as follow ^10^:  ^^  where B_MT_ is total biomass of the modeled ecosystem, B_Mi_ is the biomass of each species/Fg i in the model, and TL_i_ is the trophic level of species i as an output of the model (note: B_Mi_, DC_ji_ and TL_i_ vary in time). |
| **Mean trophic level of groups with TL >2** | TL_2_ | Calculated as the TLco, as the weighted mean (biomass) of the trophic level of species/Fg in the ecosystem with a trophic level ≥ 2 ^10^ |
| **Mean trophic level of groups with TL >3.25** | TL_3.25_ | Calculated as the TLco, as the weighted mean (biomass) of the trophic level of species/Fg in the ecosystem with a trophic level ≥ 3.25 ^10^ |
| **Total Catch** | TC | Sum of all catches (landings plus discards) of all Fg in the ecosystem (unit: t/km^2^/year) ^11^. |
| **Discards** | Di | Sum of all catches of all Fg that are discarded (unit: t/km^2^/year) ^2^ |
| **Trophic level of the catch** | TLC | TL of the catch for all retained species/Fg. Retained species/Fg are caught in fishing operations, although not necessarily targeted by a fishery and which are retained because they are of commercial interest (i.e. not discarded) ^8^.  Trophic level of the catch is calculated as follow ^10^:  ${TL}_{c}=\sum_{i=1}^{n} {Y_{i}\cdot{TL}_{i}}/{Y_{L}}$  where Y_L_ is total landings, Y_i_ is the landing of species i and TL_i_ is the trophic level of species i (note: Y_L_, Y_i_ and TL_i_ vary in time). |

**Table S6. List of species/functional groups defined included in the community indicators Demersal/Pelagic fish (D/P) and Invertebrates/Fish (I/F)**

| **FG/Species** | **Organism** | **Ecology** |
| --- | --- | --- |
| 13: Pelagic shark | Fish | Pelagic |
| 14: Non commercial large pelagic | Fish | Pelagic |
| 15: Bluefin tuna | Fish | Pelagic |
| 16: Swordfish | Fish | Pelagic |
| 17: Other large pelagic f. | Fish | Pelagic |
| 18: Mackerel | Fish | Pelagic |
| 19: Horse mackerel | Fish | Pelagic |
| 20: Other medium pelagic f. | Fish | Pelagic |
| 21 and 22: Sardine | Fish | Pelagic |
| 23 and 24: Anchovy | Fish | Pelagic |
| 25: Other small pelagic f. | Fish | Pelagic |
| 26: Benthopelagic f. | Fish | Pelagic |
| 27: Mesopelagic f. | Fish | Pelagic |
| 28: Anglerfish | Fish | Demersal |
| 29 and 30: Hake | Fish | Demersal |
| 31: Other commercial large demersal f. | Fish | Demersal |
| 32: Gadidae | Fish | Demersal |
| 33: Sparidae | Fish | Demersal |
| 34: Other commercial medium demersal f. | Fish | Demersal |
| 35: Flatfish | Fish | Demersal |
| 36: Non commercial medium demersal f. | Fish | Demersal |
| 37 and 38: Mullet | Fish | Demersal |
| 39: Surmullet | Fish | Demersal |
| 40: Other commercial small demersal f. | Fish | Demersal |
| 41: Non commercial small demersal f. | Fish | Demersal |
| 42: Bathydemersal f. | Fish | Demersal |
| 43: Small-spotted catshark | Fish | Demersal |
| 44: Blackmouth catshark | Fish | Demersal |
| 45: Other small demersal shark | Fish | Demersal |
| 46: Ray&Skate | Fish | Demersal |
| 47: Torpedo | Fish | Demersal |
| 48: Benthic cephalopod | Invertebrates | Benthic |
| 49: Benthopelagic cephalopod | Invertebrates | Benthopelagic |
| 50: Mesopelagic cephalopod | Invertebrates | Mesopelagic |
| 51: Bivalve | Invertebrates | Benthic |
| 52: Gastropod | Invertebrates | Benthic |
| 53: Deep-water rose shrimp | Invertebrates | Benthic |
| 54: Blue and red shrimp | Invertebrates | Benthic |
| 55: Giant red shrimp | Invertebrates | Benthic |
| 56: Commercial prawn | Invertebrates | Benthic |
| 57: Non commercial shrimp/prawn | Invertebrates | Benthic |
| 58: Norway lobster | Invertebrates | Benthic |
| 59: Other commercial decapod | Invertebrates | Benthic |
| 60: Non commercial decapod | Invertebrates | Benthic |
| 61: Mobile benthos | Invertebrates | Benthic |
| 62: Sessile benthos | Invertebrates | Benthic |
| 63: Jellyfish&Salp | Invertebrates | Pelagic |
| 64: Coral&Gorgonian | Invertebrates | Benthic |

**References**

1 Heymans, J. J., Coll, M., Libralato, S., Morissette, L. & Christensen, V. Global patterns in ecological indicators of marine food webs: a modelling approach. *PloS one* **9**, e95845 (2014).

2 Coll, M. & Steenbeek, J. Standardized ecological indicators to assess aquatic food webs: The ECOIND software plug-in for Ecopath with Ecosim models. *Environmental Modelling & Software* **89**, 120-130 (2017).

3 Hilborn, R. & Walters, C. J. Quantitative fisheries stock assessment: choice, dynamics and uncertainty. *Reviews in Fish Biology and Fisheries* **2**, 177-178 (1992).

4 Rochet, M.-J. & Trenkel, V. M. Which community indicators can measure the impact of fishing? A review and proposals. *Canadian Journal of Fisheries and Aquatic Sciences* **60**, 86-99 (2003).

5 Kempton, R. & Taylor, L. Models and statistics for species diversity. *Nature* **262**, 818-820 (1976).

6 Ainsworth, C. H. & Pitcher, T. J. Modifying Kempton's species diversity index for use with ecosystem simulation models. *Ecological Indicators* **6**, 623-630 (2006).

7 Shannon, C. & Weaver, W. (Univ. Illinois Press, ll, 1949).

8 Shin, Y.-J. *et al.* Using indicators for evaluating, comparing, and communicating the ecological status of exploited marine ecosystems. 2. Setting the scene. *ICES Journal of Marine Science: Journal du Conseil* **67**, 692-716 (2010).

9 Shin, Y.-J. *et al.* Can simple be useful and reliable? Using ecological indicators to represent and compare the states of marine ecosystems. *ICES Journal of Marine Science: Journal du Conseil* **67**, 717-731 (2010).

10 Shannon, L. *et al.* Trophic level-based indicators to track fishing impacts across marine ecosystems. *Marine Ecology Progress Series* **512**, 115-140 (2014).

11 Bundy, A. *et al.* The good (ish), the bad, and the ugly: a tripartite classification of ecosystem trends. *ICES Journal of Marine Science: Journal du Conseil* **67**, 745-768 (2010).

**Modelling the Mediterranean Sea ecosystem at high spatial resolution to inform the ecosystem-based management in the region**

**Chiara Piroddi, Marta Coll, Diego Macias Moy, Jeroen Steenbeek, Elisa Garcia-Gorriz, Alessandro Mannini, Daniel Vilas, Villy Christensen**

**S6.**


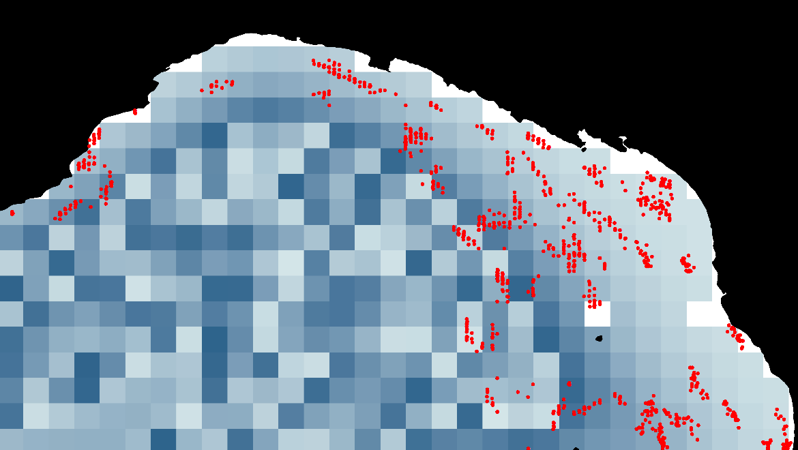


**Fig S5.** Example of data resolution of predicted model outputs (blue cells) vs Medits trawl survey (red dots).

**Ecospace outputs:**

**Marine mammals, sea turtles and marine birds**

**
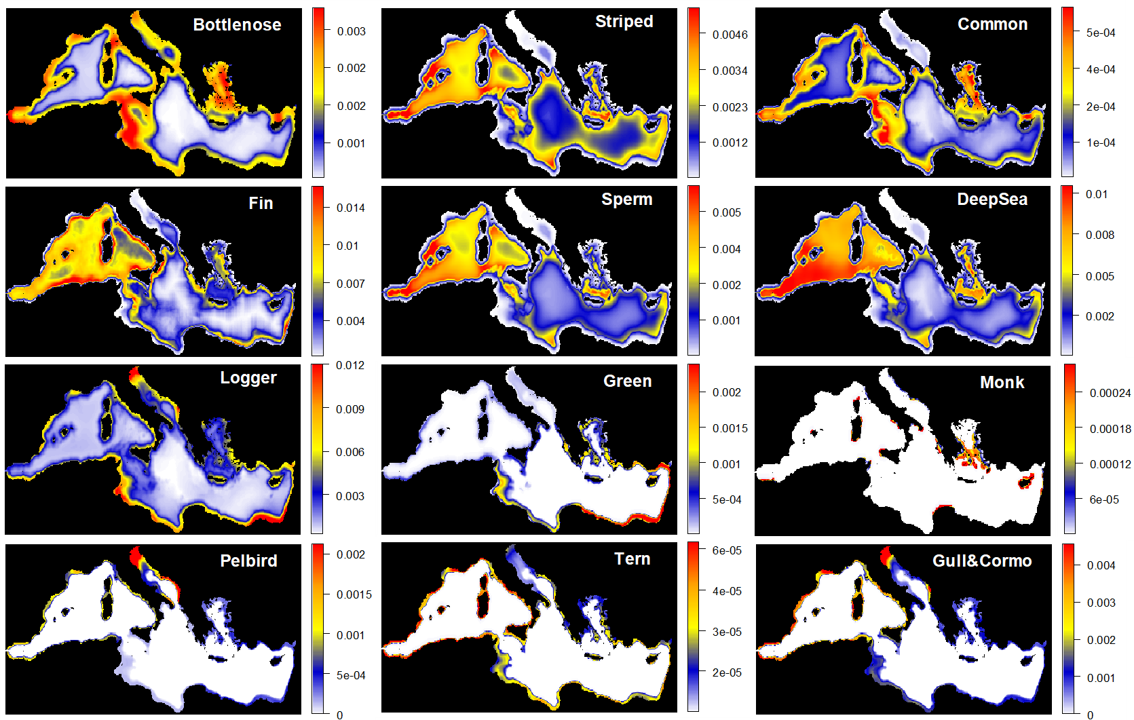
**

**Fig S6.** Mean (1995-2016) predicted biomass (t/km^2^) for marine mammals, sea turtles and marine birds. Bottlenose: Bottlenose dolphim; Striped: Striped dolphin; Common: Common dolphin; Fin: Fin whale; Sperm: Sperm whale; DeepSea: Deep sea cetaceans; Logger: Loggerhead turtle; Green: Green turtle; Monk: Monk seal; Pelbird: Pelagic bird; Gull&Cormo: Gull and Cormorant.

**Pelagic fish**

**
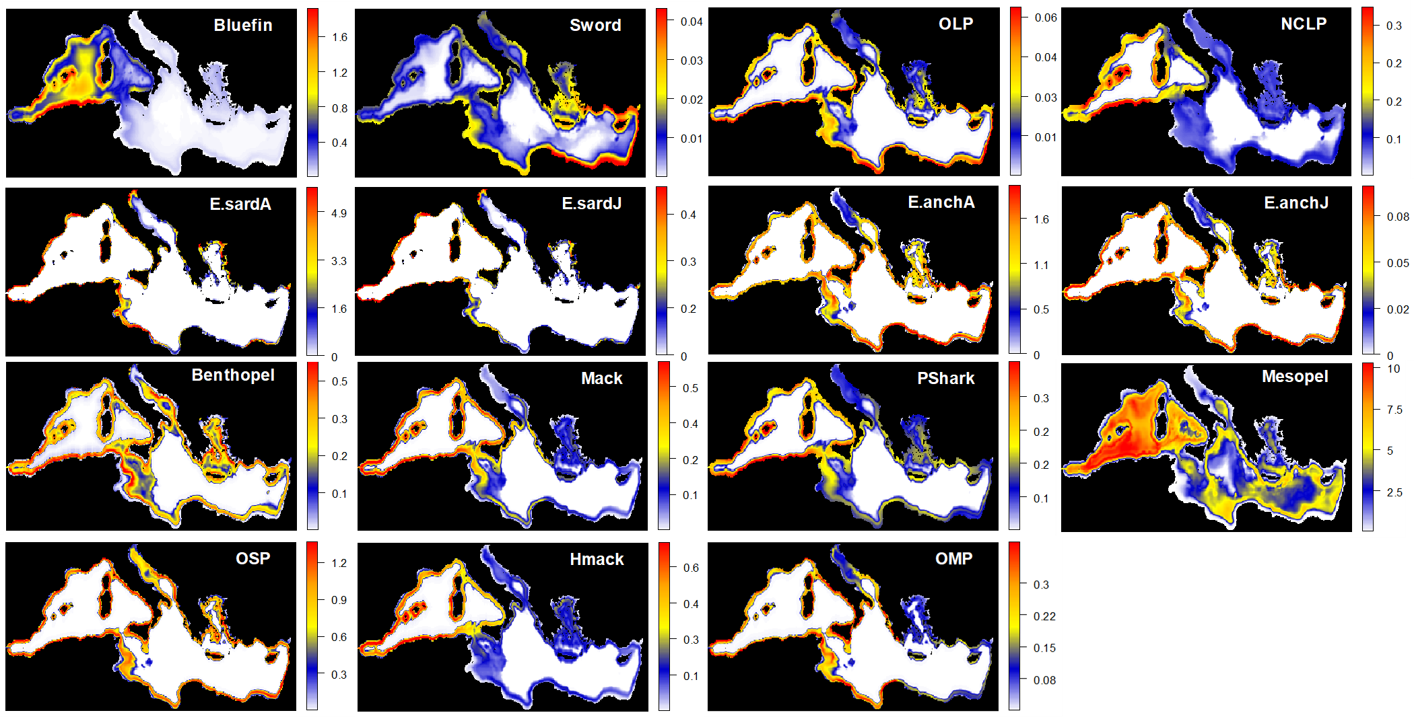
**

**Fig S7.** Mean (1995-2016) predicted biomass (t/km^2^) for pelagic fish groups. Bluefin: Bluefin tuna; Sword: Swordfish; OLP: Other large pelagic; NCLP: Non commercial large pelagic; E.sard: European pilchard; E.anch: European anchovy; Benthopel: Benthopelagic fish; Mack: Mackerel; PShark: Pelagic shark; Mesopel: Mesopelagic fish; OSP: Other small pelagic; Hmack: Horse mackerel; OMP: Other medium pelagic.

**Demersal fish**

**
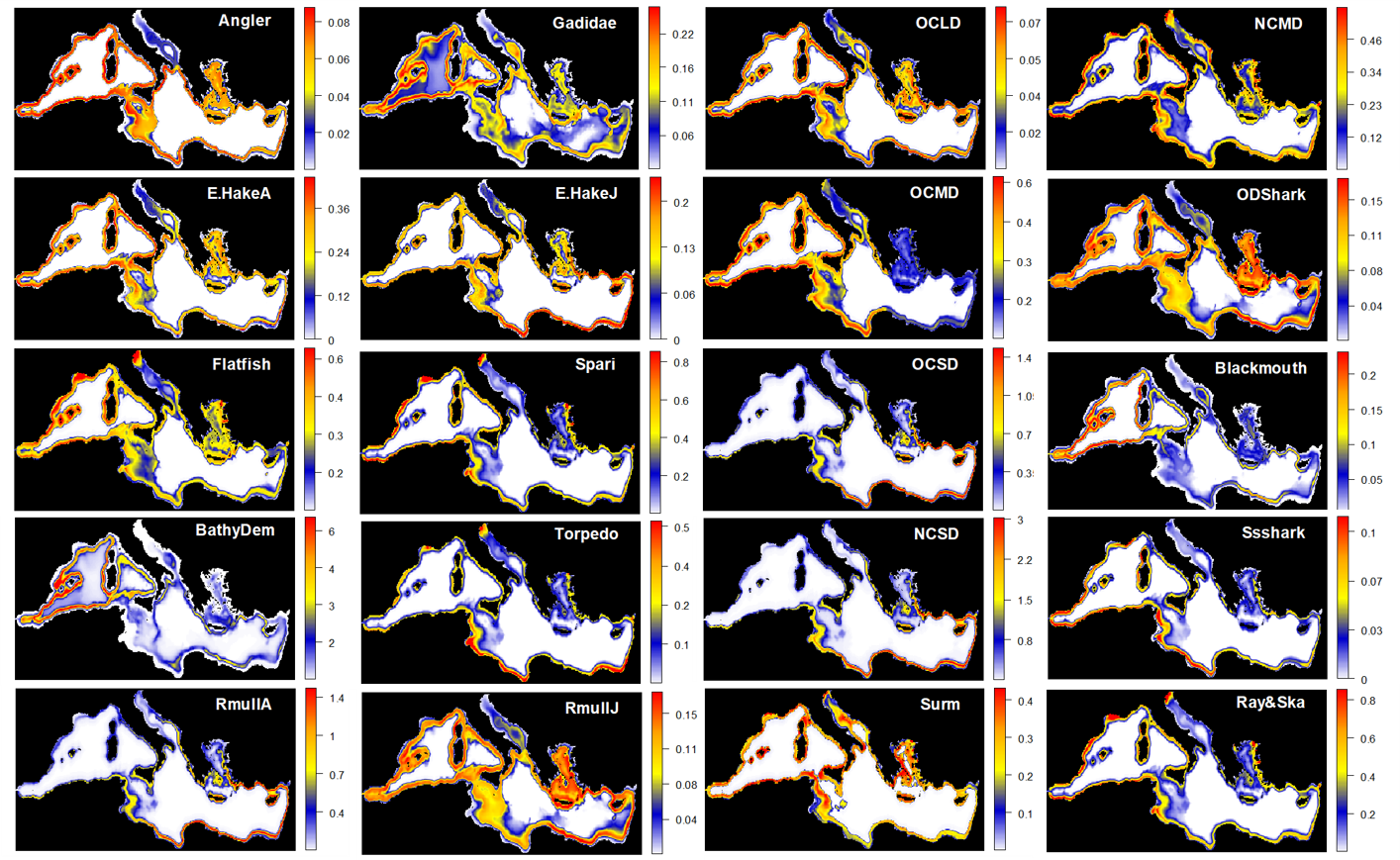
**

**Fig S8.** Mean (1995-2016) predicted biomass (t/km^2^) for demersal fish groups. OCLD: Other commercial large demersal; NCMD: Non-commercial medium demersal; OCMD: Other commercial medium demersal; ODshark: Other demersal sharks; Spari: Sparidae; OCSD: Other commercial small demersal; Blackmouth: Blackmouth catshark; BathyDem: Bathydemersal fish; NCSD: Non-commercial small demersal; Ssshark: Small-spotted catshark; Rmull: Red mullet; Surm: Surmullet; Ray&Ska: Ray&Skate.

**Invertebrates**


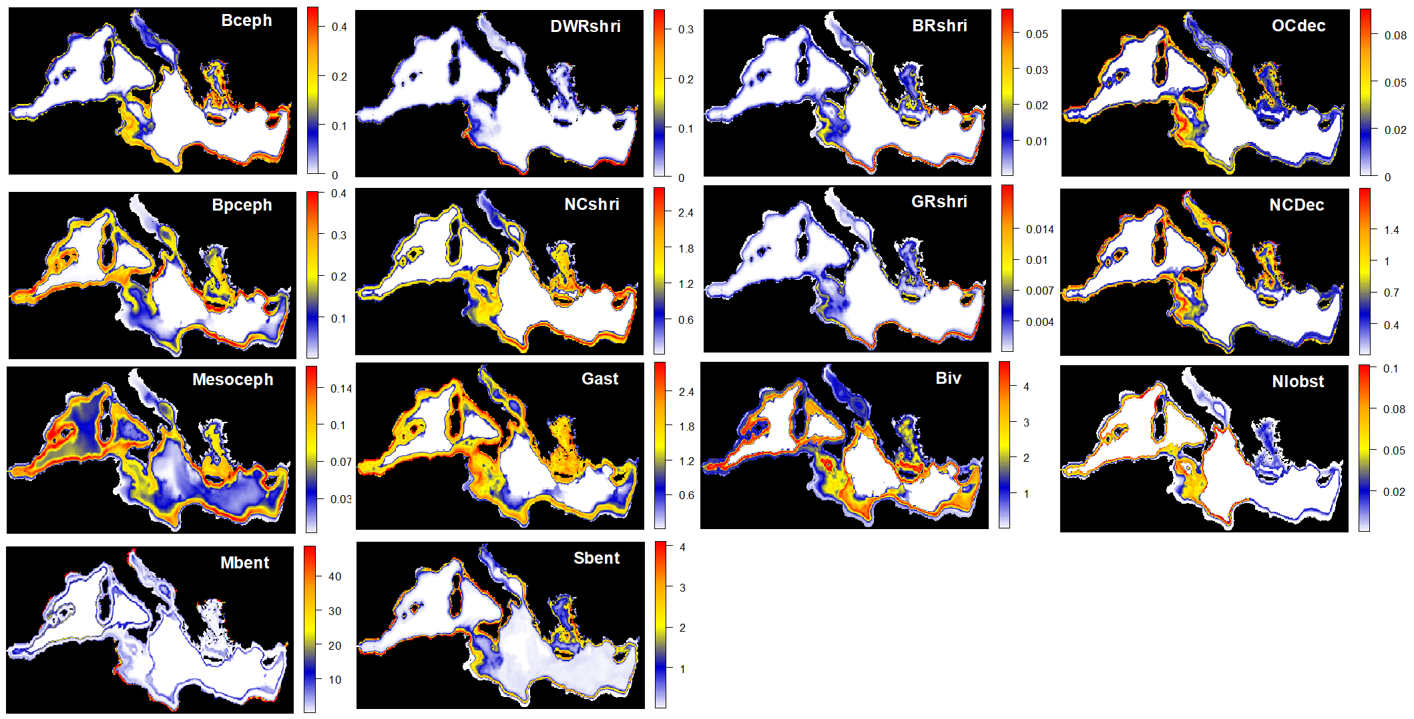


**Fig S9.** Mean (1995-2016) predicted biomass (t/km^2^) for invertebrates groups. Bceph: Benthic cephalopod; DWRshri: Deep water rose shrimp; BRshri: Blue and red shrimp; OCdec: Other commercial decapod; Bpceph: Benthopelagic cephalopod; NCshri: Non commercial shrimp; GRshri: Giant red shrimp; NCDec: Non commercial decapod; Mesoceph: Mesopelagic cephalopod; Gast: Gastropod; Biv: Bivalve; Nlobst: Norway lobster; Mbent: Mobile benthos; Sbent: Sessile benthos.

**Planktonic-Habitat**

**
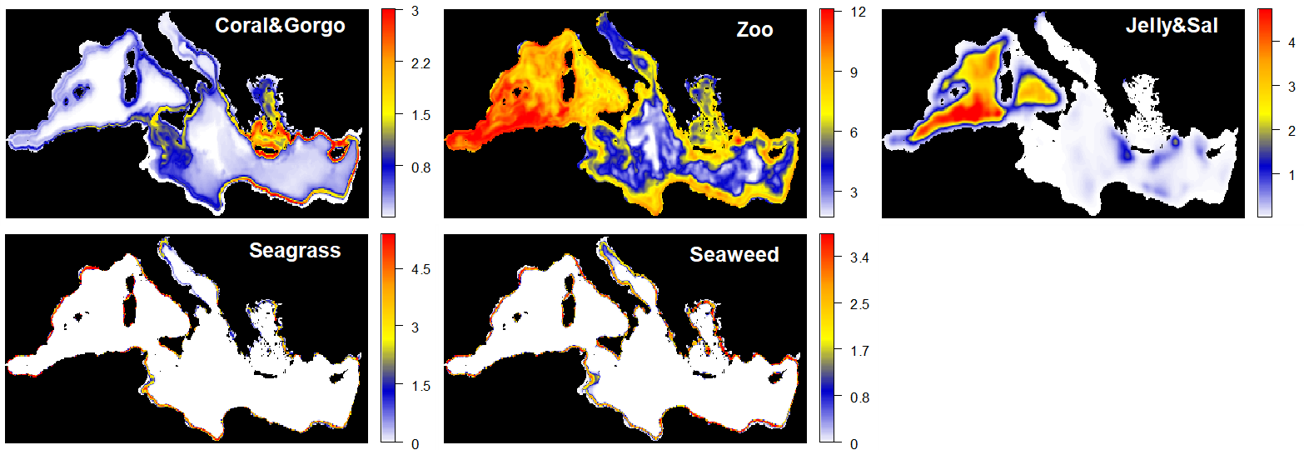
**

**Fig S10.** Mean (1995-2016) predicted biomass (t/km^2^) for planktonic-habitat groups. Coral&Gorgo: Coral and gorgonian; Zoo: Zooplankton; Jelly&Sal: Jelly and Salp.

**
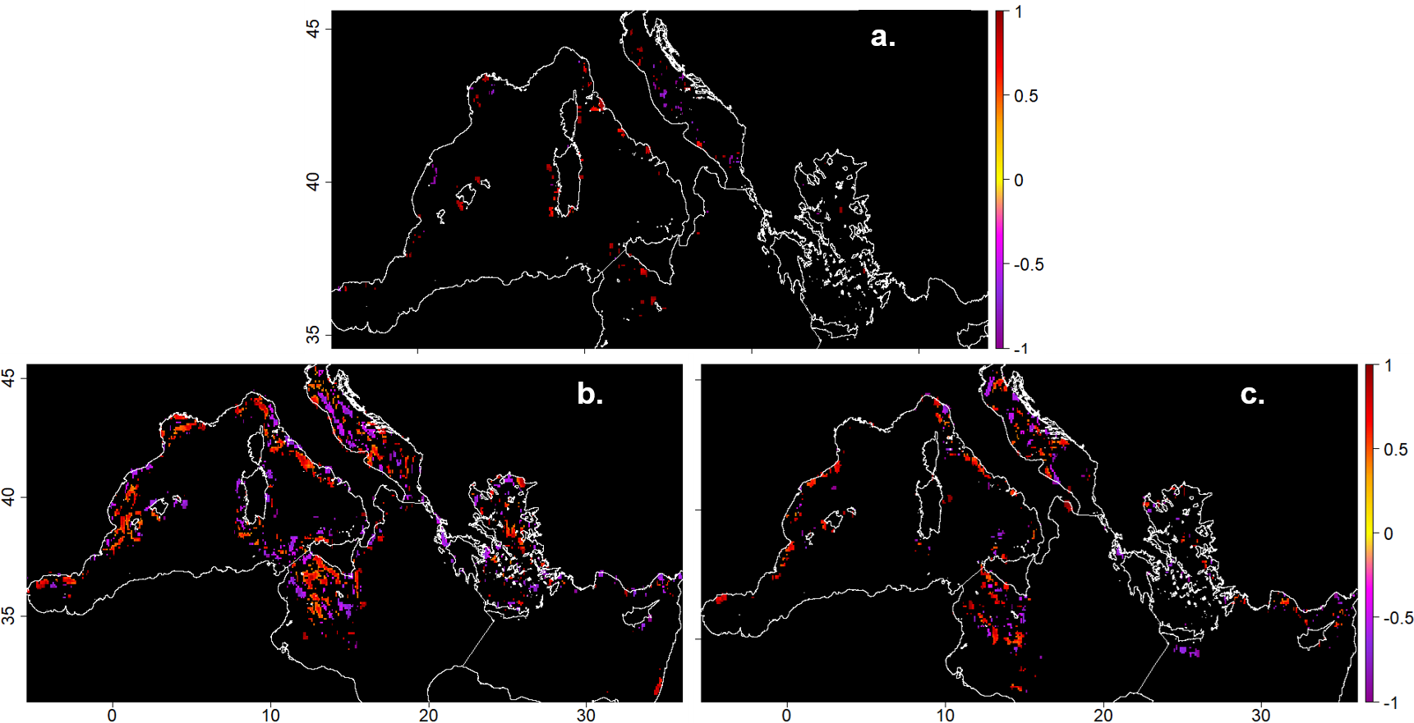
**

**Fig S11.** Mean (1995-2016) correlation between observed and predicted European hake (a.) and between AIS and predicted bottom trawlers (b) and purse seiners (c.) effort. Only correlations with p-value <0.05 are shown.

**
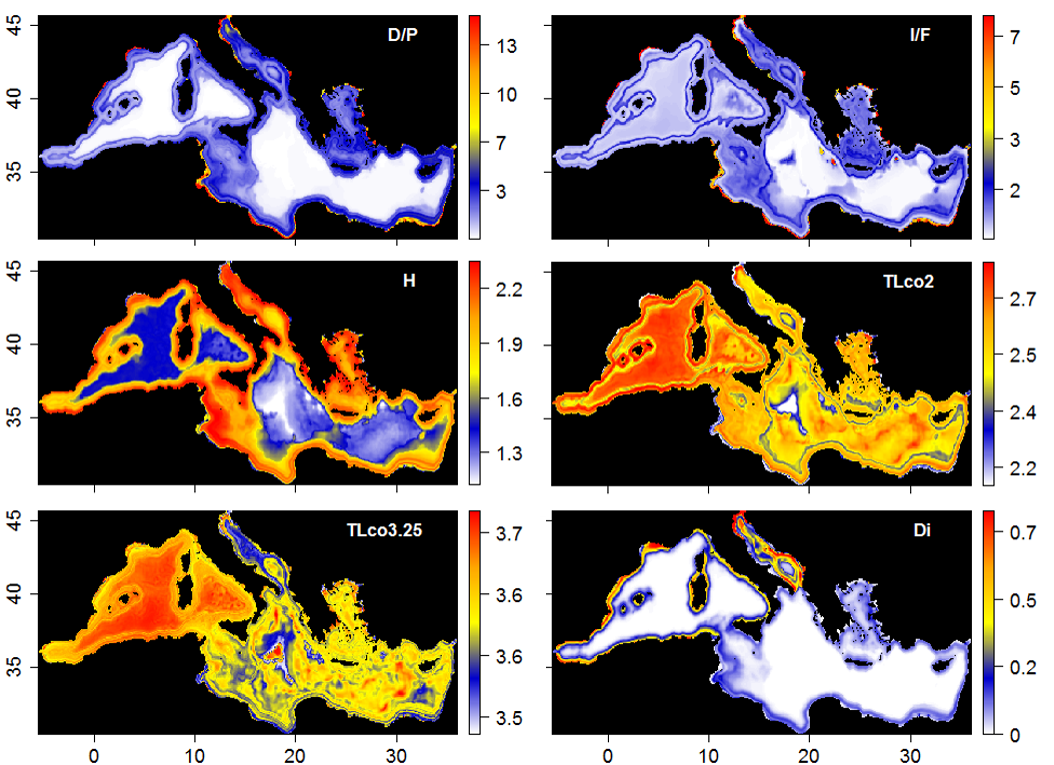
**

**Fig S12.** Mean (1995-2016) of selected modelled indicators. D/P: Demersal/Pelagic fish (t/km^2^); I/F: Invertebrates/Fish (t/km^2^); H: Shannon index; TLco2: Trophic level (TL) of species/Fg in the community with TL≥ 2; Trophic level (TL) of species/Fg in the community with TL≥ 3.25; Di: Discards (t/km^2^/year).


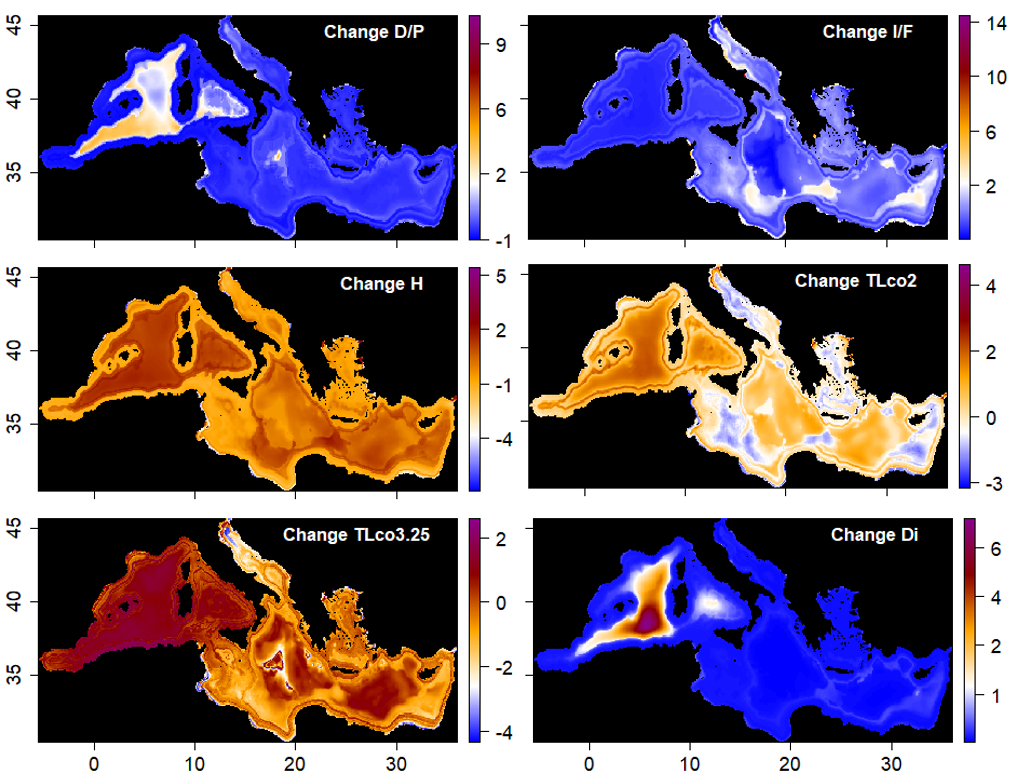


**Fig S13.** Mean (1995-2016) change (%) for selected modelled indicators. D/P: Demersal/Pelagic fish; I/F: Invertebrates/Fish; H: Shannon index; TLco2: Trophic level (TL) of species/Fg in the community with TL≥ 2; Trophic level (TL) of species/Fg in the community with TL≥ 3.25; Di: Discards. To facilitate the comparison, indicators were standardized (difference from the mean divided by the corresponding standard deviation).
